# Microscopic picture of water-ethylene glycol interaction near a model DNA by computer simulation: Concentration dependence, structure, and localized thermodynamics

**DOI:** 10.1371/journal.pone.0206359

**Published:** 2018-11-14

**Authors:** Atul Kumar Jaiswal, Rakesh Srivastava, Preeti Pandey, Pradipta Bandyopadhyay

**Affiliations:** School of Computational and Integrative Sciences, Jawaharlal Nehru University, New Delhi, India; Weizmann Institute of Science, ISRAEL

## Abstract

It is known that crowded molecular environment affects the structure, thermodynamics, and dynamics of macromolecules. Most of the previous works on molecular crowding have majorly focused on the behavior of the macromolecule with less emphasis on the behavior of the crowder and water molecules. In the current study, we have precisely focused on the behavior of the crowder, (ethylene glycol (EG)), salt ions, and water in the presence of a DNA with the increase of the EG concentration. We have probed the behavior of water and crowder using molecular dynamics (MD) simulation and by calculating localized thermodynamic properties. Our results show an interesting competition between EG and water molecules to make hydrogen bonds (H-bond) with DNA. Although the total number of H-bonds involving DNA with both EG and water remains essentially same irrespective of the increase in EG concentration, there is a proportional change in the H-bonding pattern between water-water, EG-EG, and EG-water near DNA and in bulk. At low concentrations of EG, the displacement of water molecules near DNA is relatively easy. However, the displacement of water becomes more difficult as the concentration of EG increases. The density of Na^+^ (Cl^-^) near DNA increases (decreases) as the concentration of EG is increased. The density of Cl^-^ near Na^+^ increases with the increase in EG concentration. It was also found that the average free energy per water in the first solvation shell increases with the increase in EG concentration. Putting all these together, a microscopic picture of EG, water, salt interaction in the presence of DNA, as a function of EG concentration, has emerged.

## Introduction

Living cells can be thought as a heterogeneous concentrated medium due to the presence of different types of molecules. For instance, the concentration of molecules inside a cell can vary from 80–400 mg/ml [1,2], corresponding to about 5–40% volume occupancy of a cell [3]. This intracellular environment consists of different molecular species like protein, DNA, RNA, polysaccharide, small messenger molecules, lipids, *etc*. A considerable amount of *in vitro* experiments has been done to understand the behavior of the crowded intracellular environment. However, several articles and reviews have suggested that the *in vitro* experiments done in dilute aqueous solution may not always represent the crowded physiological condition [4–7]. Molecular crowding has a substantial effect on the behavior and confinement of various molecules inside the cell. One of the major effects of molecular crowding is that due to the presence of other molecules, a molecule cannot occupy a considerable region of space which is accessible in a dilute solution. Due to this volume exclusion, the effective concentration of molecules changes which may alter the equilibrium of various biological processes [8]. Crowding can have a major influence on the kinetics of biological processes [4,9–16]. Other effects such as intermolecular interactions along with volume exclusion also play a role in molecular crowding [10].

A substantial amount of work has been done for proteins in crowded environments as can be seen from the references given in the previous paragraph. For nucleic acids, there are relatively less number of investigations under crowded condition [17]. The work done on nucleic acids have mainly investigated the effect of different solvent environments on DNA structure and dynamics. In an interesting work, Wales group have investigated DNA structure in apolar solvents such as CCl_4_ [18] and have shown that the DNA molecule gets stiffer and opts for an alternative conformation which is not very far from its aqueous counterpart. Kang *et al*. probed the swelling of a model DNA in crowded polydisperse environment [19]. Cheatham *et al*. examined the A to B transition of a short DNA in mixed water/ethanol mixture in an earlier work [20]. Noy *et al*. also investigated the same problem in a more recent work [21]. Role of external agents such as pyridine (which unfold DNA structure by replacing base stacking interactions) on DNA structure has also been investigated [22] which suggests that it can actually stabilize the DNA under acidic environment. However, in most of the works, the emphasis was more on DNA, microscopic features of mixed solvents in the presence of DNA has not been examined extensively.

Among the works looking into the details of solvent, one prominent experimental work came from the Spink *et al*. [23]. In that work, it was found that small neutral co-solutes such as ethylene glycol (EG) destabilize DNA by changing the thermodynamic activity of the water molecules and direct interaction with DNA. On the other hand, high molecular-weight co-solutes such as poly-ethylene-glycol (PEG) mostly work by the large excluded volume it creates. It was found that EG reduces the activity of water, and structurally changes the hydrogen bond network of water. Hence, both direct and indirect interactions are prevalent with EG on a DNA plus water system. As the EG plus water system has lower dielectric constant than water only, the electrostatic interactions are expected to be less screened in the presence of EG.

Among other representative works on the effect of crowding on DNA, Tateishi-Karimta and Sugimoto [24] have studied three small duplex and a quadruplex DNA in different crowding concentrations of PEG 200 and PEG 8000 and shown that by mimicking intracellular crowding a noticeable change can be observed in the stability, structure and function of these nucleic acids which could be very useful for nanotechnology purposes. Liu *et al*. [25] studied the effect of crowders on DNA melting and shown that for DNA with the sequence of A_20_ (20 base pair long homopolymer of adenine), melting temperature increases by 1 K in the presence of ficoll70 while in the ficoll70-polyvinyl pyrrolidone360 mixture the melting temperature increases by 7.5 K. They further concluded that a large-sized crowder imposes greater crowding effect and thus results in higher melting temperature for DNA. Furthermore, they observed that with increasing crowding concentration in the system, DNA melting temperature increases. Harve *et al*. [26] studied the effect of crowding and confinement on the DNA/DNA and DNA/RNA hybrid and have shown that crowding and confinement increase the hydrogen bond formation between nucleotides. Thus crowding may facilitate nucleotide hybrid structure irrespective of their length, sequence or type. In one of the computational works, Yildirim *et al*. [27] used a reduced dielectric continuum medium to understand the conformational preference of DNA in the cellular-like environment and suggested that in the reduced dielectric medium, a canonical B form of DNA structure shifts towards non-canonical A form of the DNA structure.

In the current work, we have investigated the structure and thermodynamics of both DNA and the mixed solvent, water and EG. This work differs from most of the previous works in the sense that both DNA and solvents are treated on equal footing. The current work also differs from the most recent work done by Nakano *et al*.[28], where they have looked at the localized water properties for G-quadruplex and DNA hairpin. However, our work focuses predominantly on the structure of the first solvation shell of a small DNA segment, containing both water and crowder molecules, along with the competition among crowder, water molecules, and ions to bind to DNA from a thermodynamic point of view at different crowding concentrations. We have taken a small DNA segment (sequence 5’-GACCGAGCAGCCCGTACTCAGTC-3') as our model system. This DNA was put in a solvent containing water, EG (whose concentration was varied) and 0.1 M NaCl. From a statistical mechanical point of view, this is an extremely complex system with five components, having long-range interaction. The many-body interactions present in the system have been modeled using classical forcefields. We were interested in understanding both the structural features and thermodynamics of this system, as the concentration of EG increases. A particular aim was to explore the microscopic picture of EG and water near DNA to understand their binding to DNA and binding among themselves. Structural fluctuation of DNA and the role of ions *vis-à-vis* the solvent molecules have also been looked into. One intriguing aspect of this study was competition between water and EG in the presence of highly charged DNA in solvating DNA. Various pair-correlation functions (PCFs) between different components of the system were calculated to get a consistent picture of DNA solvation and solvation thermodynamics. As PCF is connected to the potential of mean force, each PCF indicates the average effect of the rest of the system on the degree of freedom considered for PCF calculation. Along with this standard PCF, which has contribution from all parts of the system, localized thermodynamic properties based on the inhomogeneous solvation theory of Lazaridis [29] near DNA were also calculated. Three-dimensional Reference Interaction Site Model (3D-RISM) was employed to calculate the distribution of water [30]. Moreover, geometrical parameters such as hydrogen bonding patterns among different species were also calculated. Both global and local thermodynamics, structural properties were used to get a comprehensive view of the microscopic picture of this five-component system.

It was found that the EG molecules intrude into the first hydration shell of DNA with the increase in the concentration of EG. The total number of hydrogen bonds (H-bonds) remains almost same irrespective of crowder concentration. Crowder molecules assemble closer to DNA replacing water. However, the replacement of water is much less after the first solvation shell. The population of Na^+^ ion near DNA increases with the increase in EG concentration. However, the population of Cl^-^ decreases suggesting that the region close to DNA acquires more negative charge density as crowder molecules are added. Average free energy per water was found to proceed from negative to positive values as the crowder concentration was increased, but the system was made stable with the compensatory effect of EG in the first solvation shell. Subtle effects of thermodynamics, mostly enthalpic, are modulating the complex behavior of this system.

## Methods

In this section, a brief description of molecular dynamics simulation and the methods used, *i*.*e*., Grid inhomogeneous solvation theory (GIST) and Reference Interaction Site Model (RISM) to dissect the role of water, EG and salt on a small DNA duplex are given. For details of these methods, readers are referred to the original literature.

### Grid inhomogeneous solvation theory (GIST)

GIST was developed by Nguyen *et al*. [31], which is based on the pioneering inhomogeneous solvation theory (IST) proposed by Lazaridis *et al*. [29,32]. In IST, solute introduces a field to the solvent causing the density fluctuation of the solvent. The thermodynamic properties in IST can be thought as coming from two sources. The first is the local interaction between solute-solvent (local effect), and the other is ‘dilution’ of solvent due to the presence of solute (the liberation term—global effect). In this method, the total effect of solute on solvent has been approximately divided into local and non-local effects, to calculate properties of a region of solvent (*e*.*g*., the first solvation shell) on insertion of a solute.

In the GIST method, all the terms given in IST are not considered for the evaluation of entropy and enthalpy. For entropy, only the solute-solvent term is considered (water reorganization entropy is not considered). For solvation enthalpy calculation, both solute-solvent and solvent-solvent interactions (the liberation part of solvent-solvent interaction is not considered) are taken into account. The numerical evaluations of the three dimensional (3D) integrals appearing in the entropy and energy expression in inhomogeneous solvation theory of Lazaridis are performed by using discrete sums over 3D grid voxels. A 3D region R of interest which may contain both solute and solvent is divided into small grids or voxels. The thermodynamic quantities are calculated for each of the voxels from the Molecular dynamics (MD) trajectory.

Pair correlation function or radial distribution function *g*(*r*_*k*_) of water for a voxel centered at the position r_k_ is defined as:

$$g\left(r_{k}\right)=\frac{N_{k}}{\rho^{0}V_{k}N_{f}}$$

where, *N*_*k*_ is the total number of water molecules summed over all *N*_*f*_ frames of a trajectory whose oxygen atoms are in voxel *k*, *ρ*^*0*^ is bulk water number density, and *V*_*k*_ is the volume of voxel *k*.

GIST uses *g*(*r*_*k*_) values and forcefield parameters defined for MD simulation to compute thermodynamic quantities for each voxel of the defined region R. The values of thermodynamic quantities for all the voxel of the region R are added to get the total. Finally, dividing the values of these thermodynamic quantities for region R by the average number of water molecules present in region R, we get normalized values of corresponding thermodynamic quantities per water.

The normalized average free energy (which is used in the current work) per water molecule with respect to bulk water in region R is defined as

$$\Delta G^{R,norm}=\Delta E_{sw}^{R,norm}+\Delta E_{ww}^{R,norm}-T\Delta S_{trans}^{R,norm}-T\Delta S_{orient}^{R,norm}$$

where, $\Delta E_{sw}^{R,norm}$ and $\Delta E_{ww}^{R,norm}$ represent the difference in the solute-water and water-water interaction potential in region R with respect to the bulk water system and $T\Delta S_{trans}^{R,norm}$ and $T\Delta S_{orient}^{R,norm}$ represent the change in translation and orientational entropy per water in region R with respect to the bulk water system.

It is to be noted that there are some limitations in the GIST method like the solute is kept restrained and the crowders and salt ions are stripped out from trajectory because everything else except water is considered as the solute in GIST. So, GIST does not consider the direct presence of crowders and salt ions in trajectory frames. However, the volume excluded by the crowders is included in the calculation. The results obtained from GIST should be taken with these approximations.

### 3D-RISM: Three-dimensional Reference Interaction Site Model

Given below is a brief introduction to the 3D-RISM method. To know about further details, the reader may go through the original literature [33–35]. 3D-RISM calculation uses the Ornstein-Zernike (OZ) equation, which is given below for a one component molecular liquid;

h(1,2)=c(1,2)+ρ∫c(1,3)h(3,2)d(3)

where, *h(1*,*2)* and *c(1*,*2)* represent the total and direct correlation function, respectively. *ρ* is the density of the liquid. The numeric inside the parenthesis represent the position and orientational coordinates. In the 3D-RISM method, the density distribution of solvent (which may include salt) is calculated around a solute with a fixed geometry. *g(1*,*2)* is related to the total correlation function *h(1*,*2)* as *g(1*,*2) = h(1*,*2) − 1*.

Prior to the 3D-RISM calculation, solvent properties are calculated using one-dimensional (1D) RISM which gives the pair correlation function among different sites of the molecular liquid (these sites are usually atom centered but need not be). The OZ equation needs another equation relating *h* and *c* (closure relation) to make its solution possible. Some of the choices are Percus-Yevick (PY), Hypernetted chain equation (HNC) and Kovalenka-Hirata (KH). In this work, KH closure was used with the dielectrically consistent (DRISM) version of RISM. The standard protocol of performing RISM calculation was followed in the current work. At first, 1D-RISM calculation of water with 0.1 M NaCl concentration was performed. This gives the pair correlation function and susceptibility function which are needed for the 3D-RISM calculation.

### Molecular dynamics (MD) simulation details

**Fig 1:**
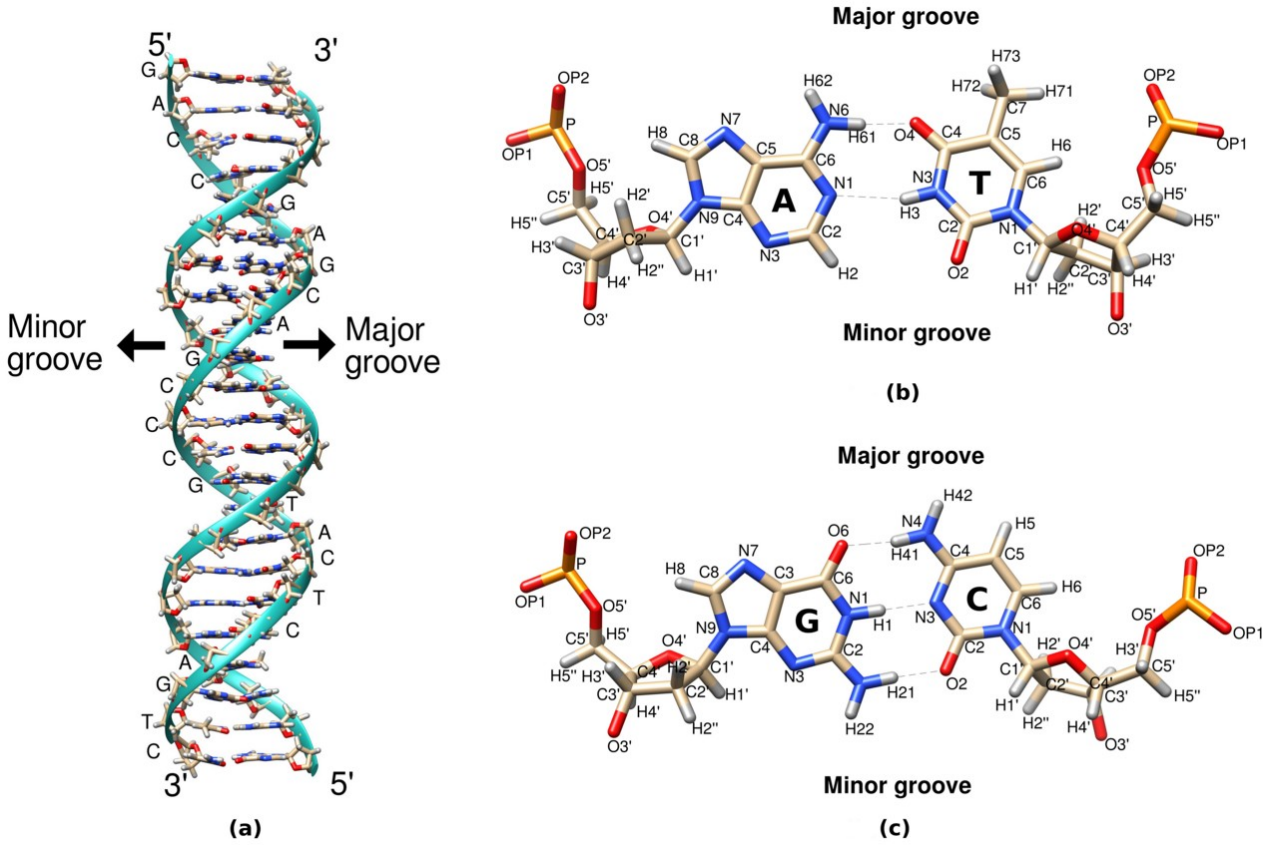
Schematic representation of model DNA. (a) DNA model depicted in ribbon diagram (b) AT and (c) GC base pair drawn in stick model.

To investigate the effect of crowding on DNA structure, solvation, thermodynamics of solvation and the complex interplay between various molecular species in solvating the DNA, a DNA of 23 base pairs (5'-GACCGAGCAGCCCGTACTCAGTC-3') (a schematic representation of the modeled DNA and atoms defining the major and minor groove is shown in Fig 1) was used. In this study, ethylene glycol (EG) was used as the crowder. The structure of the DNA was generated *via* nab program available in AMBER14 [36]. The atomic charge of EG was calculated by RHF/6-31G* level of theory using RESP procedure [37], as implemented in the RED server [37–40] (the atomic charges are given in the Table A in S1 File). The forcefield parameters, other than the charges, were generated using antechamber [41] and gaff [42]. For molecular packing of water (TIP3P), EG and DNA, the Packmol package [43] was used. For molecular dynamics simulation, the initial structures were prepared using the tleap module of AMBER14. DNA was simulated in four different environments with varying concentration of EG (0%, 10%, 20% and 30% EG by volume of the simulation box) and salt (NaCl) concentration of 0.1 M was used. Joung and Cheatham ion parameters were used for Na^+^ and Cl^-^ ions [44]. The compositions of the four systems are given in Table 1. In the rest of manuscript, the different systems will be denoted by the initial volume occupancy of the crowders (*i*.*e*., 0%, 10%, 20% and 30%).

**Table 1:** List of systems and their composition.

Crowder Percentage		Number of DNA (23bp) molecule	Number of water molecule	Number of EG molecule	Number of Na^+^	Number of Cl^-^
Initial	Final					
0%	0%	1	35636	0	157	113
10%	10.22%	1	32068	2011	157	113
20%	18.95%	1	28500	4022	157	113
30%	27.00%	1	24932	6066	157	113

**Note**: Since, MD simulations have been performed in NPT ensemble, the simulation box size has changed from its initial size (100 X 100 X 120 Å^3^) and therefore, the final crowder percentage has changed from the initial percentage.

All MD simulations were performed using AMBER14 package [36]. The ff12sb (ff99bsc0) forcefield [45–48] was used for DNA. Particle-mesh Ewald (PME) method [49] was used for calculating electrostatic interactions, and SHAKE algorithm [50] was used for restraining bonds involving hydrogen atoms. The temperature was controlled using Langevin dynamics [51], and constant pressure was maintained using Berendsen’s barostat [52].

Energy minimization of all the four systems was performed in three stages: firstly, by restraining the DNA and EG, the solvent (water) and ions were minimized. In the second stage, water molecules, ions, and EG were minimized by restraining the DNA, and in the third stage, the entire system was minimized. This was followed by gradually heating the system in six stages, with an increment of 50 K at each stage in the NVT ensemble. During the course of heating, DNA and EG molecules were restrained. After heating, the systems were equilibrated for 60 ps in 5 stages, in NPT ensemble. During equilibration, the restrains from DNA was sequentially decreased from 10 to 0 kcal/mol (10, 5, 1, 0.1, 0.0). After equilibration, all systems were simulated (production run) for 100 ns with 2 fs time step. Three independent trajectories were carried out for all four systems. To check convergence of our simulation one trajectory out of the three was extended to 300 ns. Hence, for each concentration, a total of 500 ns MD simulation was run. Unless otherwise mentioned, all plots shown in this manuscript are from the 500 ns MD run.

There are two issues in the accuracy of our results coming from the MD simulation. First is the choice of forcefield and the second issue is the convergence of trajectories. For the forcefield issue it is to be noted that in this work, we have used ff99bsc0 forcefield for the DNA in the simulations. As there is a newer forcefield ff99parmbsc1 [53] is available for DNA in AMBER, we compared both the structural and thermodynamic properties of DNA plus water system using both the forcefields. We examined the difference in various DNA structure, and solvent properties like root mean square deviation (RMSD), root mean square fluctuation (RMSF), end to end distance, bending angle of DNA and pair correlation function (PCF) of water around DNA backbone (discussed in section 1 of S1 File and in Figures A, B, C and D in S1 File). We observed that except for RMSD none of the calculated properties showed significant difference using either of the two forcefields. As the main emphasis of this paper was the water (and crowder)-DNA interaction in terms of their PCF (which is related to the potential of mean force), we believe our results will be essentially insensitive to the choice of different (recent) AMBER forcefields.

**Fig 2:**
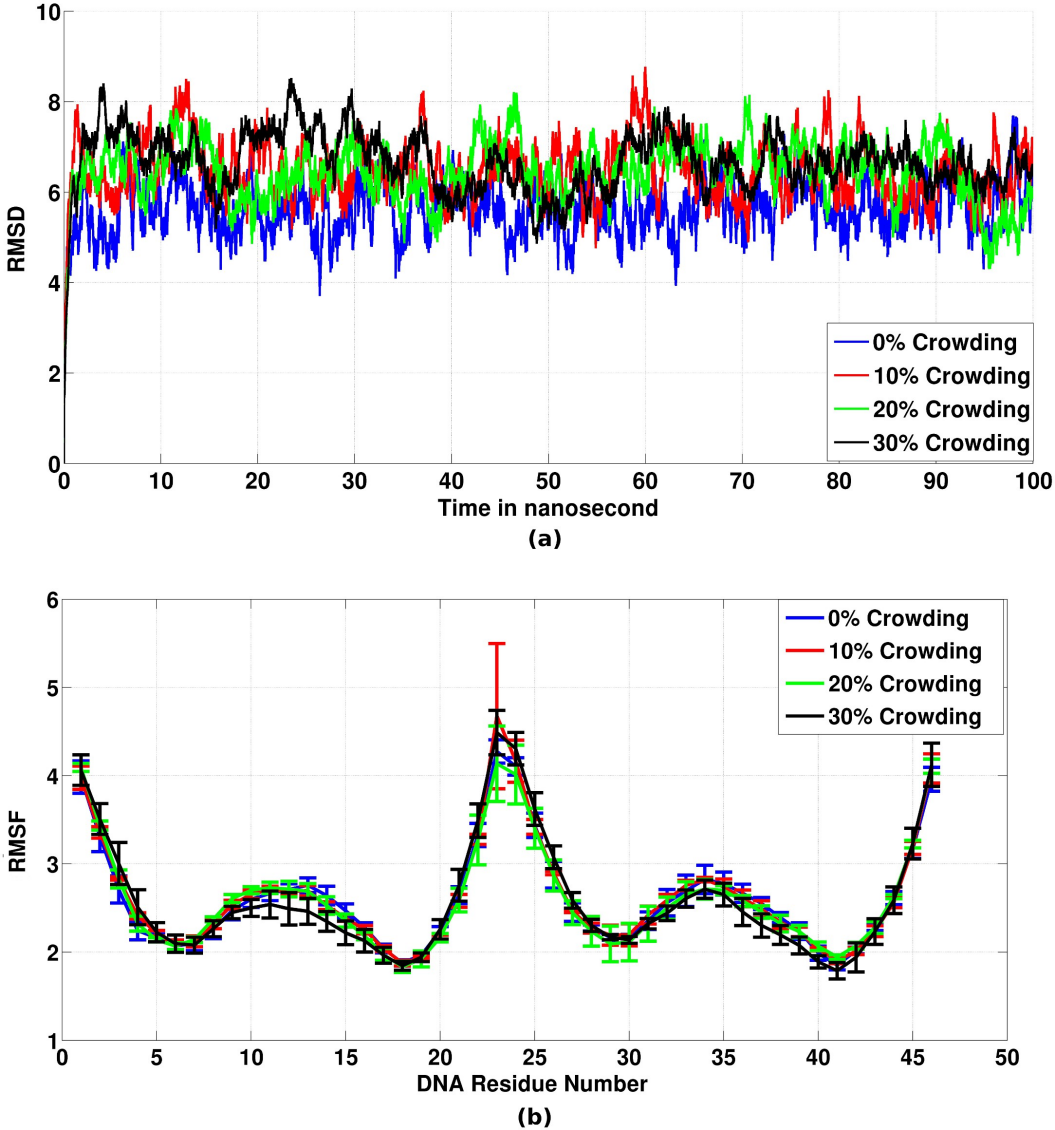
(a) Average root mean square deviation (RMSD) in Å with reference to the initial structure used in the simulation. (b) Average root mean square fluctuation (RMSF) in Å for each base of DNA.

The convergence of simulation trajectories is a critical point as far as distribution of different species (water, EG and ions) is concerned. It is known that convergence of the distribution of ions is most time consuming among the species concerned in the current work. We have checked the convergence of ions by calculating the average Na^+^ population around DNA. We calculated average Na^+^ population in the major and minor groove of DNA at different crowding concentration using CURVES+ and CANION software, which uses a curvilinear helicoidal coordinate (CHC) system to calculate the distribution [54]. In this representation, the fluctuation of DNA is much less as compared to the cartesian representation. Three parameters D, R and A are used to define the position or coordinates of the ions, where, D, R and A represents the base pair step, the distance between ion and helical axis corresponding to the base pair step and angle between vector R and vector representing the long axis of the base pair step, respectively. The values of R was taken as 10.25 Å, the value of A was taken as 33 to 147 degrees for minor groove and rest for major groove and the value of D was taken from 2 to 22 (for detailed description please follow the supplementary material of [54]). It is evident from Figure E in S1 File that for major groove convergence at each concentration follows a similar pattern and convergence is achieved within 300 ns. For the minor groove the variation is larger among different concentrations of EG. Except for the 30% EG system, other systems converge within 300 ns. For the 30% EG system, the convergence is achieved around 400 ns. For water and EG, it is known that the direct calculation of their density around DNA will be blurred as a result of the fluctuation of DNA. For this, we have identified two stretches of 4 base pairs (GAGC and TACT from the DNA sequence 5’-GACCGAGCAGCCCGTACTCAGTC-3'), which show least fluctuation as seen from the RMSF plot in Fig 2(b). For these two stretches, we have calculated both water and EG distribution using the GRID module (described in section 2 in S1 File) of CPPTRAJ around these two stretches. Figures F-L in S1 File show that both water and EG concentrations have converged nicely for all four systems. Moreover, our calculated PCFs (described in the next sections) remain unchanged as we increase the length of one trajectory by 200 ns. Hence, we can conclude that all simulations have converged for the properties we are interested in.

### Details of GIST and RISM calculations

To understand the solvation thermodynamics of DNA, GIST program [29,55] of AMBER14 was used. For GIST calculations, separate simulations were performed by restraining the DNA for all the four systems. Two trajectories of 100 ns each were run for all four systems. The bulk number density of water *ρ*^0^ and mean water-water interaction energy $E_{ww}^{0}$ were calculated from a separate 100 ns simulation of the TIP3P water box.

RISM calculations were performed in two steps. At first, standard RISM calculations with KH closure were performed at 300 K with water density of 55.5 M and NaCl concentration of 0.1 M. This gives the solvent susceptibility function in one dimension which was used in the next step for 3D-RISM calculation. 3D-RISM calculations were performed with KH [35] closure for 50 snapshots of each system with a solvent buffer distance of 30 Å. All PCFs in this work has been calculated with 0.1 Å bin size except for DNA-Cl^-^ PCF, where bin size of 0.5 Å was taken.

## Results

### 1. Variation in DNA structure at different crowding concentrations

To probe the structural variation of the DNA considered in this study, we calculated the various structural properties like root mean square deviation (RMSD), root mean square fluctuation (RMSF), major groove width and minor groove width of DNA with the increase in crowder concentration. The RMSD of the DNA with reference to the initial structure for each system is shown in Fig 2(a) after averaging over three trajectories. The DNA structure at 0% crowding concentration has average RMSD of 5.6 ± 0.58 Å (the number after 5.6 denotes standard deviation) with reference to the initial DNA structure. At 10%, 20% and 30% crowding concentration, the average RMSD are 6.5 ± 0.64 Å, 6.4 ± 0.67 Å, and 6.7 ± 0.65 Å at 10%, 20% and 30% EG concentrations, respectively. To understand more about the fluctuation in DNA structure, we further calculated the root mean square fluctuation (RMSF) of each base in DNA which is shown in Fig 2(b). There was no significant difference in RMSF of DNA bases with the increase in the concentration of EG. Further, on comparing the average structure of the DNA at different crowding concentrations (Figure M in S1 File), we observed that the overall structure of the DNA has not changed with the increase in crowding in the medium (RMSD of average structure at 10%, 20% and 30% crowding with reference to 0% crowding are 0.268 Å, 0.274 Å and 0.457 Å, respectively). In addition, we also calculated the total number of hydrogen bonds formed between the DNA bases for the corresponding four crowding systems. We observed that the total number of hydrogen bonds essentially does not change with the increase in the crowding concentration (Figure N in S1 File) (average number of hydrogen bonds for 0%, 10%, 20% and 30% crowding are 66.53 ± 2.87, 66.26 ± 2.96, 65.86 ± 3.23 and 66.73 ± 2.89, respectively). From above results, it can be concluded that there is no major difference in the DNA structure as a result of an increase in crowder concentration, apart from the increase in fluctuation of the structure in crowded condition.

**Fig 3:**
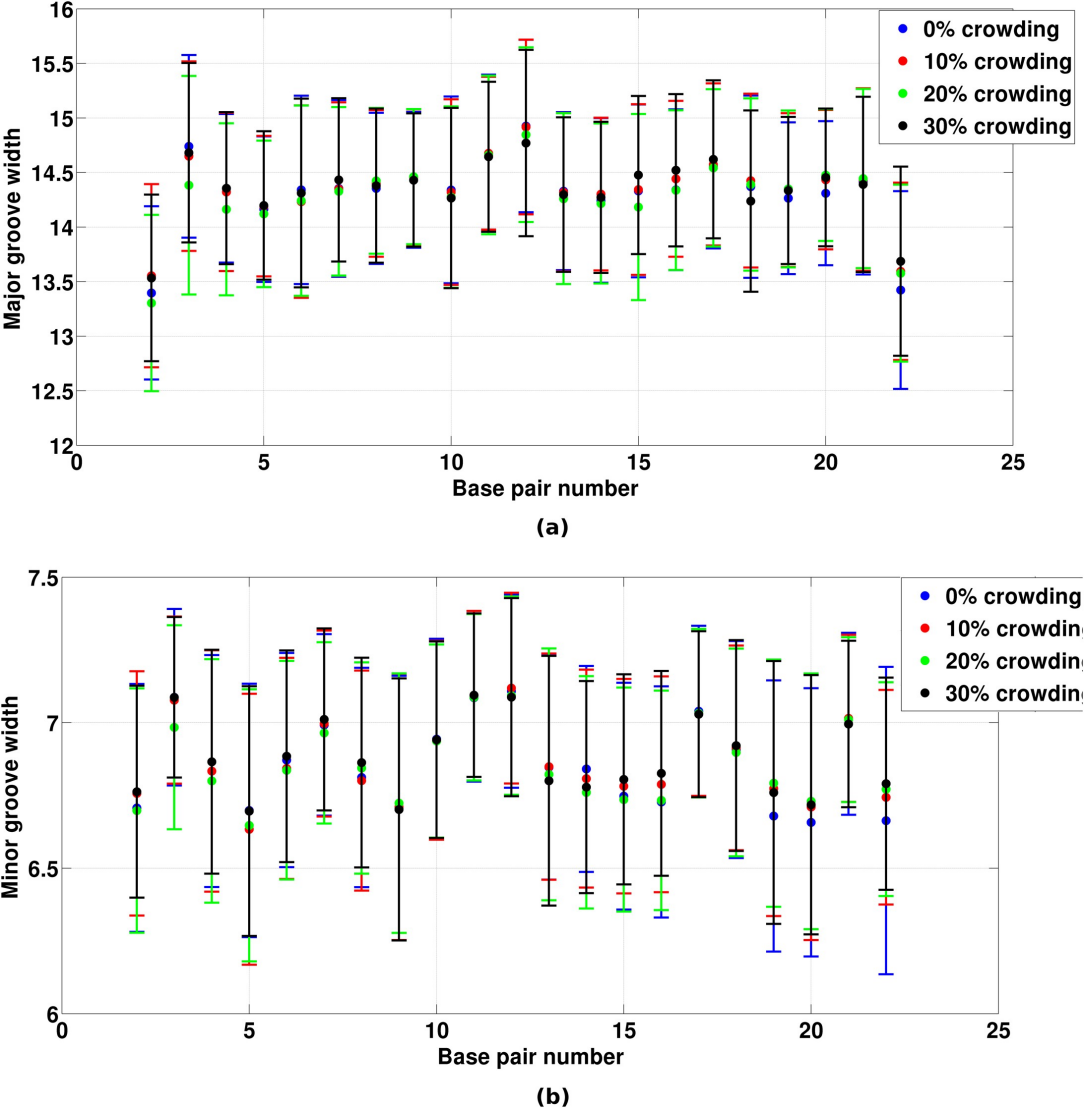
Groove widths of DNA. (a) Major groove width in Å of DNA base pairs (2–22), (b) Minor groove width in Å of DNA base pairs (2–22).

Further to understand the effect of crowding on DNA structure, we calculated DNA major and minor groove widths. Major and minor groove widths were calculated using the method developed by Hassan & Calladine [56]. This method calculates cross-strand P-P distances considering the direction of the sugar-phosphate backbone. We further corrected the major and minor groove widths by subtracting 5.8 Å from obtained widths which correspond to *van der Waals* radii of two phosphates. Major and minor groove widths of free end residues in DNA were not calculated because of their large fluctuations. Hence, in Fig 3(a) and 3(b), major and minor groove widths of 21 base pairs of DNA is shown. As can be seen from the Fig 3(a) and 3(b), no significant differences were observed in DNA major and minor groove widths at different crowding concentrations.

We analyzed other parameters of DNA structure using 3DNA tool [57]. DNA parameters like shift, slide, rise, tilt, roll, twist, x-displacement, y-displacement, inclination, and tip were calculated and are shown in the figures O and P in S1 File. In addition to DNA structural parameters, we also analyzed DNA end to end distance along with DNA bending angle (Figure Q in S1 File). The average end to end distance (in Å) was found to be 70.33 ± 1.94, 70.67 ± 2.37, 69.87 ± 2.18, and 70.44 ± 2.55 for 0%, 10%, 20% and 30% crowded systems, respectively. The average bending angle (in degree) of DNA was found to be 164.09 ± 8.64, 164.04 ± 8.28, 161.18 ± 10.00 and 163.38 ± 8.45 for 0%, 10%, 20% and 30% crowded systems, respectively. Essentially, no notable change was observed in the structural parameters of DNA, DNA end to end distance and DNA bending angle at different crowding concentrations.

#### Essential dynamics analysis of DNA

**Fig 4:**
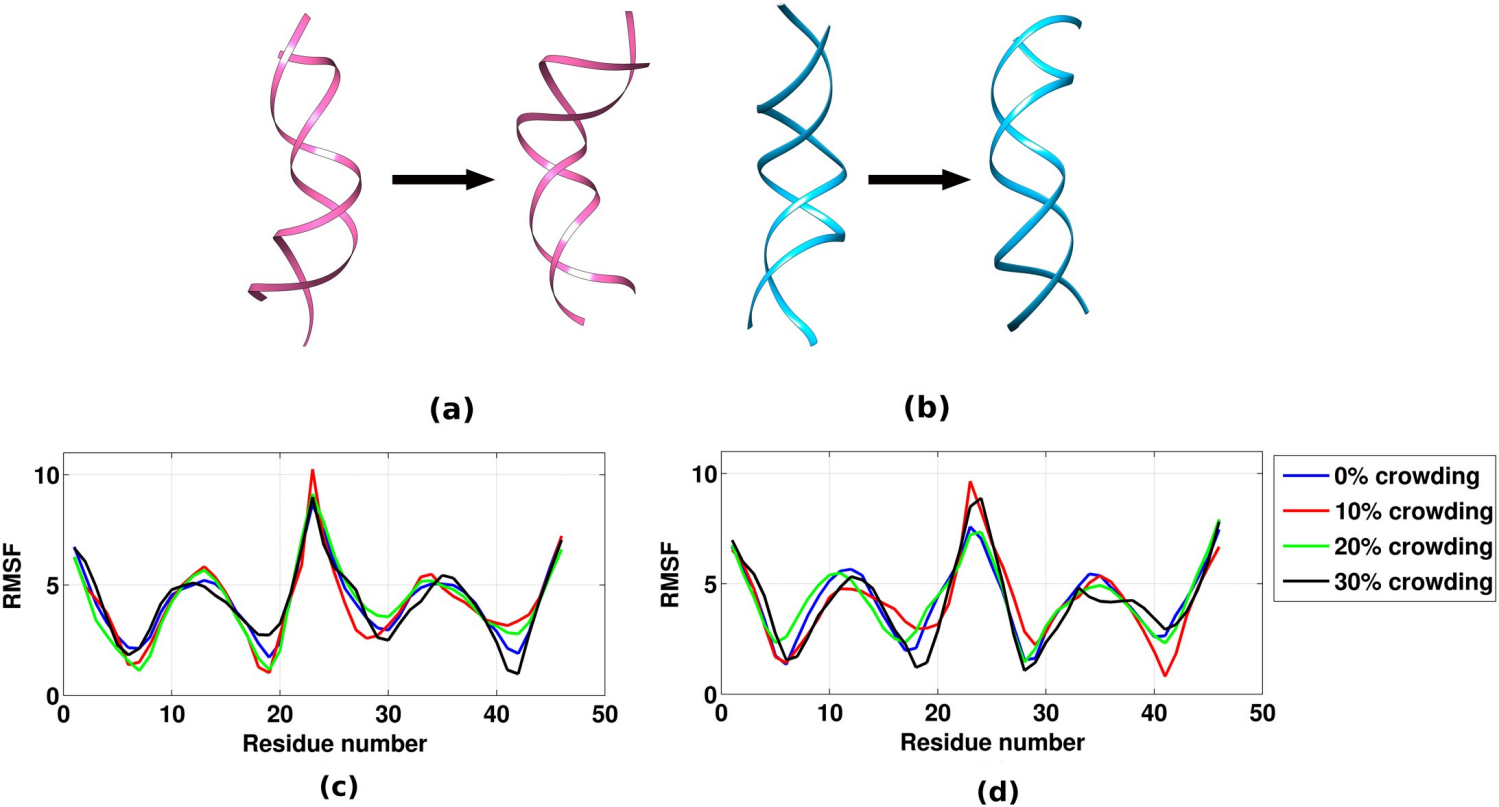
Major DNA motions captured by first two PCs and the contribution of relative motion of various bases to the PCs. (a) First mode showing bending motion of DNA. (b) Second mode showing bending motion of DNA in an orthogonal direction to the first PC. (c) RMSF in Å calculated along the first PC. (d) RMSF in Å calculated along the second PC.

Essential dynamics analysis [58] was carried out to investigate the effect of an increase in crowder concentration on the conformational dynamics and major motions of the DNA molecule as the dynamic behavior of any biological molecule is the key to their function. Essential dynamics analysis was carried out by performing principal component (PC) analysis using the cpptraj module of AMBER14. As it has been shown that approximately 100 eigenvectors can explain nearly all the variance of the DNA regardless of its sequence [59], we calculated the first 100 PCs for all the four systems. We observed that the first two PCs essentially captured 70% of the major motions associated with the DNA molecule. Both the PCs, *i*.*e*., PC1 and PC2 show the DNA bending motion, but in orthogonal directions (Fig 4), which essentially remains same in all the systems, *i*.*e*., the order of these PCs remains same in all the four systems (0% to 30% of EG). Further, the contributions of the relative motion of various bases to the first two components were also observed to be comparable in all the four systems (as depicted in Fig 4(c) and 4(d)). The above observations show that the PCs do not flip, *i*.*e*., the major motions of DNA remain essentially same with an increase in crowder concentration. This states that the DNA dynamics does not change upon changing the crowding environment which is in coherence with the above section.

PCA provides an important insight into the nature of essential movements of biomolecules. The eigenvectors projected on the cartesian coordinates gives information about the movement of biomolecule associated with each eigenvector. Unfortunately, considering the high dimensionality, comparison of motions associated with each eigenvector quantitatively for several systems is very arduous. Considering this, we further calculated two similarity indices γ and ζ [60,61] (described in the section 3 of in S1 File) between the first ten eigenvectors of the four systems to capture the change in the dynamics of the DNA as an effect of an increase in crowding concentration. Both the similarity measures capture how well the essential dynamics of given biomolecules follows a conformational transition. Their values range from 0 (no similarity) to 1 (identical movements). The absolute similarity index, γ, gives equal weight to all eigenvectors of the important space (defined by the “important set” of the eigenvectors) and thus is insensitive to the relative importance of eigenvectors and also the permutations made in the eigenvectors in the essential space. In contrary, ζ takes in account the relative importance of eigenvectors and can also detect the interchanges made in the eigenvectors. In general, the behavior of both the similarity indices are similar. However, ζ has been shown to be more powerful in differentiating anomalous conditions [60]. Figure R in S1 File shows that there is a close similarity between the nature of the motions sampled by the DNA in all the four systems. The absolute similarity index, γ, range from 0.84 to 0.94 and ζ values also range from 0.84 to 0.94. This states that the DNA dynamics does not change upon changing the crowding environment.

### 2. Structural properties of water and crowder

#### 2.1 Pair correlation function (PCF) of water and crowder around DNA

PCF (g(r)) of water and crowder molecules around DNA backbone were calculated to understand the solvation structure of DNA at different concentration of crowder molecules. The g(r) of an atom of a species (water or crowder) around an atom of DNA is defined as:

$$g\left(r\right)=\frac{\rho \left(r\right)}{\rho^{o}}$$

where, *ρ*(*r*) is the density of the atom of that species at distance *r* from the atom of DNA and *ρ*^*°*^ is the bulk density of that species.

**Fig 5:**
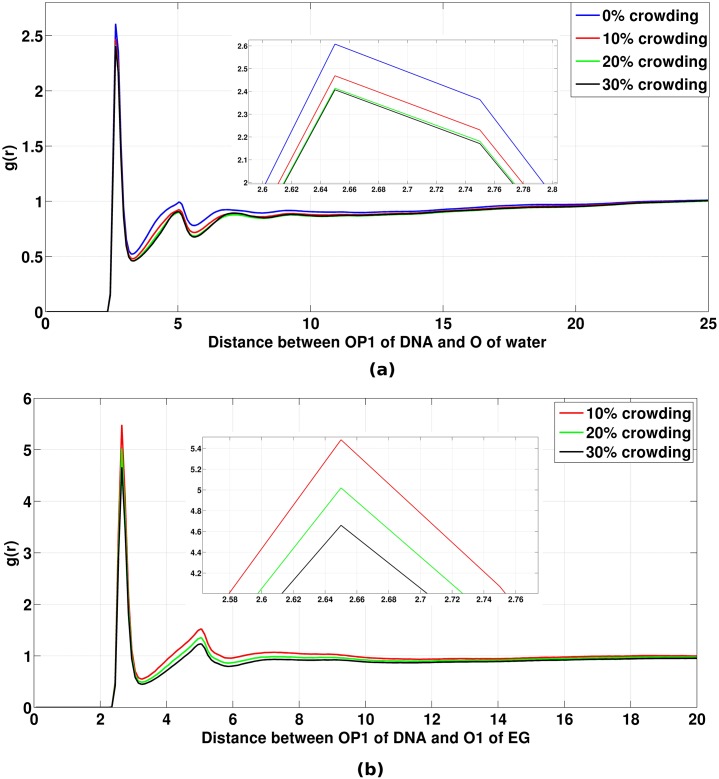
Radial distribution functions of oxygen of water and crowder around OP1 atom of DNA. (a) g(r) of oxygen of water around OP1 atom of DNA backbone. X-axis represents the distance between the oxygen of water, and OP1 atom of DNA and Y-axis represents g(r). The g(r) has been normalized with the bulk number density of water for the corresponding systems. (b) g(r) of O1 of crowder (EG) around OP1 atom of DNA backbone. The g(r) has been normalized with the bulk number density of crowder for the corresponding systems. Satellite view has also been shown in (a) and (b) which represents the zoomed view of the first g(r) peak.

The bulk density of water and crowder molecules for different systems is given in Table 2. These were obtained by dividing the total number of species of each type with the average box size in the simulation. All radial distribution functions of 0%, 10%, 20% and 30% crowded system were normalized with the corresponding bulk density of the system at 300 K. The pair correlation function of oxygen of water and oxygen (O1) of crowder around OP1 atom (averaged over all OP1 atoms) of DNA is shown in Fig 5(a) and 5(b), respectively. The pair correlation function of O1 and O4 of crowder around OP1 atom of DNA were found to be same. When DNA is kept at 0% crowding concentration the height of the first peak (*i*.*e*., the probability of finding water at that distance) is 2.60 while at 10% crowding concentration the first peak height is 2.46 (Fig 5(a)). For 20% and 30% crowded systems the first peak heights are 2.41 and 2.40, respectively. The first peak height for the crowder-DNA g(r) plot is 5.44 at 10% concentration of crowder (Fig 5(b)). This indicates that the probability of finding the crowder near DNA is much more than to find it in bulk as compared to finding water in these two regions. At 20% and 30% crowding concentrations, the first peak heights are 4.50 and 4.63. This shows that upon increasing the crowding concentration, the density of crowder around OP1 atom of DNA decreases with respect to the bulk density in their corresponding systems. This implies that the intrusion of crowder molecules into the first solvation shell of DNA decreases with respect to their bulk crowder densities with increasing crowding concentrations.

**Table 2:** The average bulk number density of water and crowder molecules.

System	Water (molecules/Å^3^)	Crowder (molecules/Å^3^)
0%	3.27 x 10^−2^	0
10%	2.73 x 10^−2^	1.72 x 10^−3^
20%	2.27 x 10^−2^	3.20 x 10^−3^
30%	1.86 x 10^−2^	4.53 x 10^−3^

**Note**: Average bulk number density has been calculated using the average volume of the box throughout the simulation.

We have further calculated coordination number of water and crowder molecules in the first solvation shell of OP1 of DNA by the following integration using Trapezoid’s rule:

$$CN=\int_{0}^{rm}\rho_{0}4\pi r^{2}g(r)dr$$

where, r_m_ is the position of the first minimum of g(r), and *ρ*_*o*_ is the bulk density of water or crowder in the corresponding systems.

The coordination number of water and crowder molecules in the first solvation shell of OP1 of DNA is given in Table 3. It can be seen that with increasing crowding concentration, the coordination number of O of water in the first solvation shell around OP1 of DNA continuously decreases. But the sum of coordination numbers of O of water and O of crowder almost remains constant. This shows that the loss of the coordination number of O of water around OP1 of DNA is mostly compensated by the oxygen atoms present in the crowder molecules. This implies that the crowder molecules are arranging themselves in such a way that the interaction lost with the change in water distribution is mostly compensated.

**Table 3:** The coordination number of water and crowder around OP1 atom of DNA.

System	Coordination number of Water	Coordination number of Crowder
0%	3.15	-
10%	2.47	0.60
20%	2.14	0.98
30%	1.75	1.28

**Note**: The coordination number has been calculated by integrating g(r) till the first minimum after the first peak.

#### 2.2 Distribution of ions around DNA and solvent

In this work, DNA, solvent, and ions were treated with equal footing. Hence, it becomes an important aspect to understand the behavior of ions around the DNA and solvent (water and EG) molecules with the increase in crowder concentration. We first tried to understand the behavior of ions around the DNA as an effect of increase in crowding concentration. For this, two types of analysis were performed, ion density analysis and PCF (g(r)) calculation.

Ion density analysis was performed using curvilinear helicoidal coordinates (CHC) formalism using CURVES+ and CANION software [54,62,63]. The position of each ion was determined from each simulation trajectory with reference to the helical axis of DNA. The curvilinear helicoidal space was defined with a bin size of 0.5 Å in R, ⅙ in D (base pair step) and 5 degrees in A.

**Fig 6:**
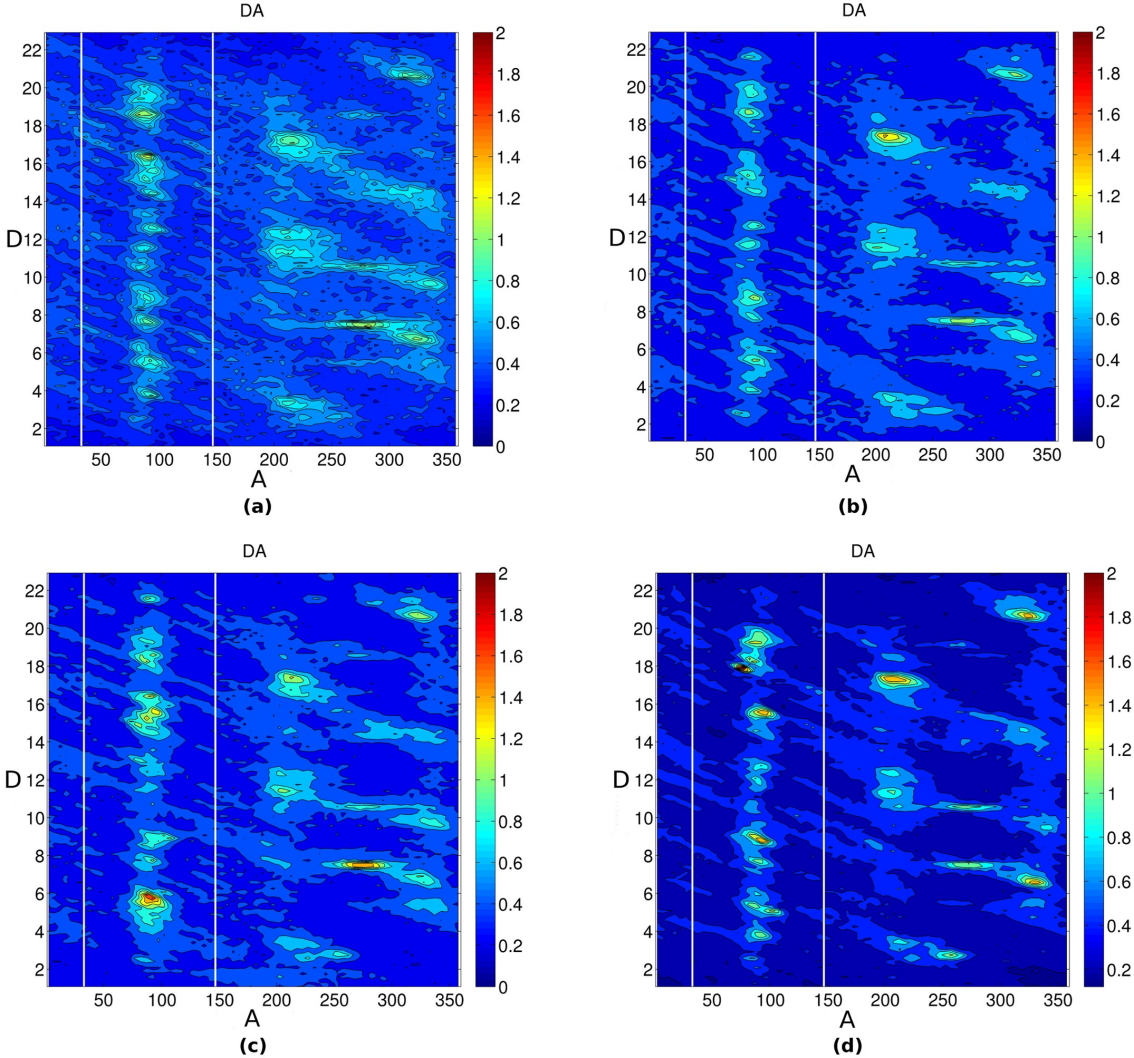
Average Na^+^ distribution calculated using CHC in DA plane (A in degrees and D is base pair step). (a) 0% crowding (b) 10% crowding (c) 20% crowding (d) 30% crowding. The color scale blue to red represents increasing molarity.

**Fig 7:**
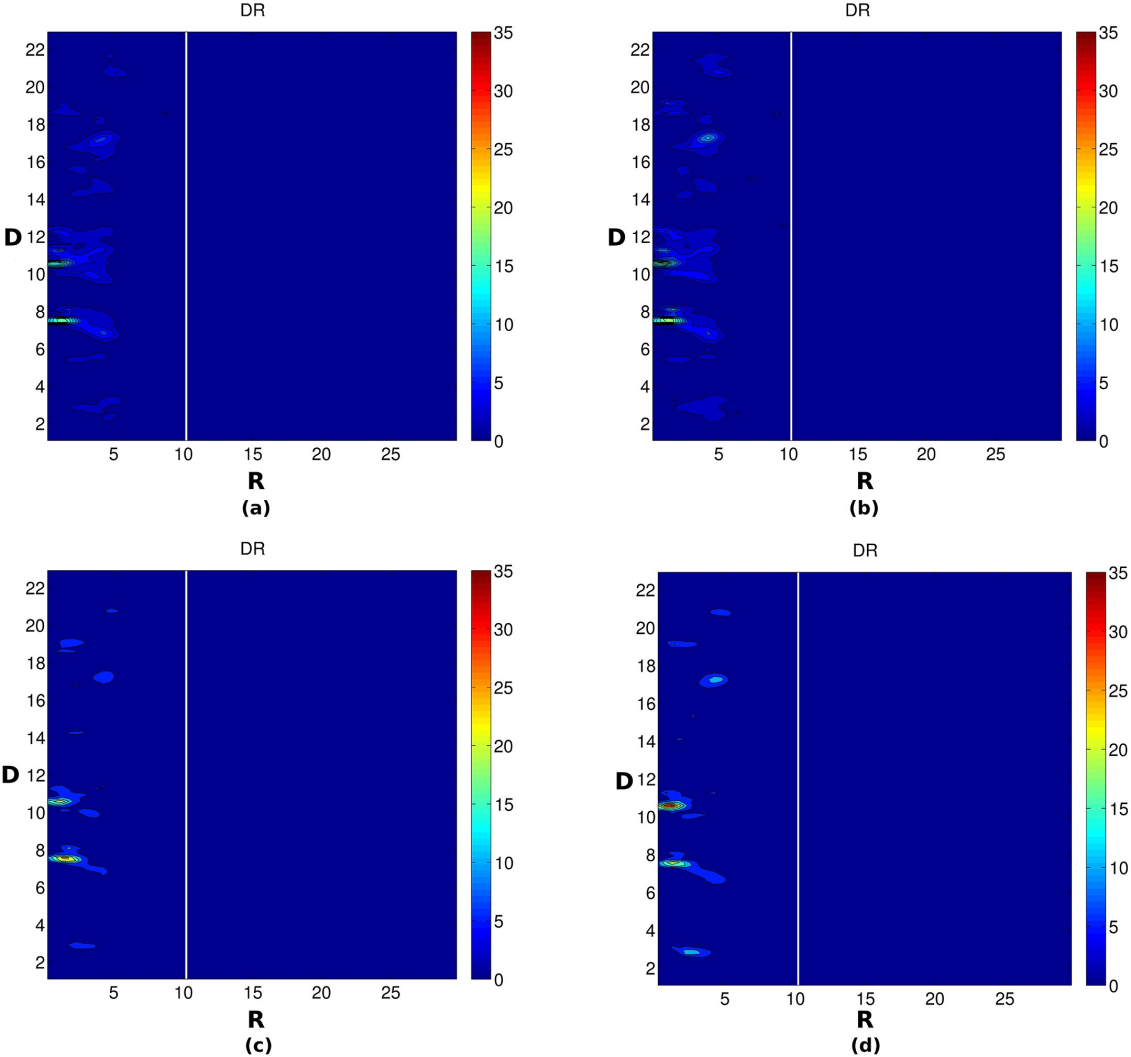
Average Na^+^ distribution calculated using CHC in DR plane (R in Å and D is base pair step). (a) 0% crowding (b) 10% crowding (c) 20% crowding (d) 30% crowding. The color scale blue to red represents increasing molarity.

**Fig 8:**
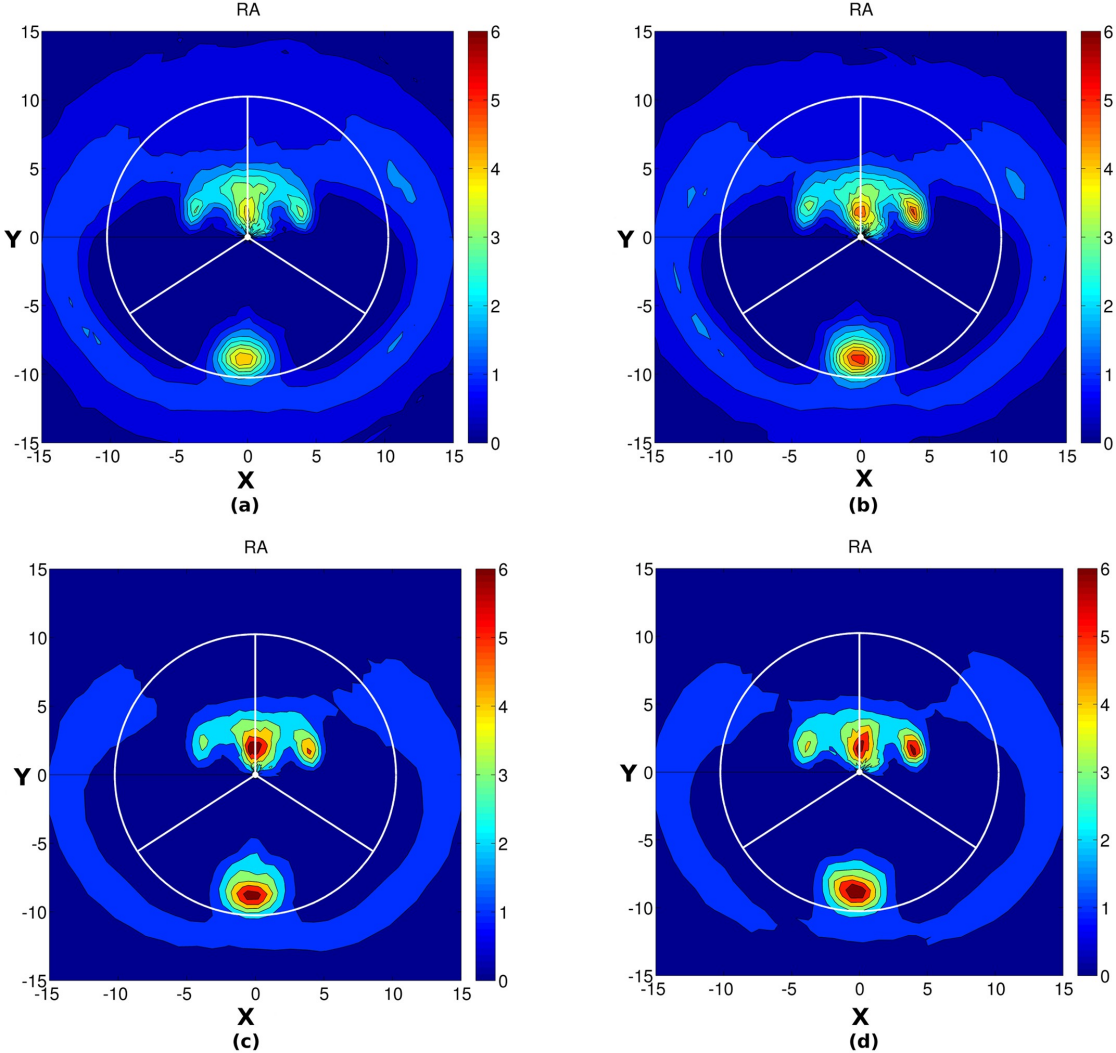
Average Na^+^ distribution calculated using CHC after transforming the R and A to cartesian coordinates (both X and Y are in Å). (a) 0% crowding (b) 10% crowding (c) 20% crowding (d) 30% crowding. The color scale blue to red represents increasing molarity. The upper semicircle represents the major groove and lower represents the minor groove of DNA. The vertical radial vector indicates the center of the major groove.

We analyzed the Na^+^ distribution in the major and minor groove of DNA by plotting the Na^+^ ion distribution in the helicoidal space using all the three possible 2D parameters (DA, DR and RA) for all four systems. Fig 6 shows the molarity of Na^+^ ions in the minor groove (A range from 33° to 147°) and major groove (A range from 147° to 33°) of DNA. As can be seen from the DA plot (Fig 6), the Na^+^ ions follow a column-like distribution in the minor groove of DNA at a constant angle A ~ 85° and the ion density increases with the increase in crowding concentration. The Na^+^ density also increases in some patches in the major groove of DNA with the increase in crowding concentration. A similar picture also arises from the DR plot (Fig 7). It can be seen that the Na^+^ ions are distributed within a constant distance R = 5 Å from the helical axis and its density increases with the increase in crowding concentration. Further, Fig 8 shows the 2D RA density (in the form of molarity) distribution of Na^+^ in the major groove (the upper semi-circle) and minor groove (lower semi-circle). Consistent with the DA and DR plot, from the RA (R and A are transformed back to cartesian coordinate for simple visualization purpose) plot it is apparent that the Na^+^ distribution in some regions, both in the major and minor groove of DNA increases with the increase in crowding concentration. From all these figures, it can be concluded that with the increase in crowding concentration, the density of Na^+^ ion increases in the major and minor groove of DNA. Upon examination of the distribution of Cl^-^ ion, it was seen that the density of Cl^-^ decreases with the increase in crowding concentration (Figure S in S1 File).

We further calculated the g(r) of Na^+^ and Cl^-^ atoms around OP1 atom of DNA. Consistent with the ion density analysis, upon examination of the g(r) of Na^+^ and Cl^-^ ions around OP1 atom of DNA (Figures T(a) and T(b) in S1 File), we observed that the local density of Na^+^ increases and Cl^-^ decreases (both with respect to the density of these ions in the bulk) upon increasing the crowding concentration. Both the analysis suggests that the addition of EG has sufficiently affected the electrostatics of the system. As a result, DNA can accommodate more Na^+^ ions in its vicinity. However, DNA repels Cl^-^ ions more with the increase in EG concentration. Next, we examined the distribution of Na^+^ and Cl^-^ around the water molecules. As can be seen from Figure U(a) and U(b) in S1 File, the density of Na^+^ increases by a small fraction; however, the density of Cl^-^ increases significantly with the increase in EG concentration. Similar behavior of ion distribution (Na^+^ and Cl^-^) was observed around EG (Figures V(a) and V(b) in S1 File), except that the density of Cl^-^ around water is more compared to EG, at all concentrations of EG.

The complex electrostatics involving five components is non-trivial to explain quantitatively. However, one model could be the following. Addition of EG increases the negative charge density around DNA, which is the reason why more Na^+^ accumulates near DNA with the increase in EG concentration. A larger number of Na^+^ near water and EG (as DNA is covered by water and EG) makes the electric field around water and EG more positive, which attracts more Cl^-^. This model is supported by the Na^+^—Cl^-^ PCF (Figure V(c) in S1 File), which shows an increase of the Cl^-^ density around Na^+^ as EG concentration is increased. As expected, the addition of EG not only results in volume exclusion but also redefines the electrostatics of the system. Thus, it cannot be simply explained only with a reduced dielectric constant.

#### 2.3 Hydrogen bond between DNA-water and DNA-crowder

**Fig 9:**
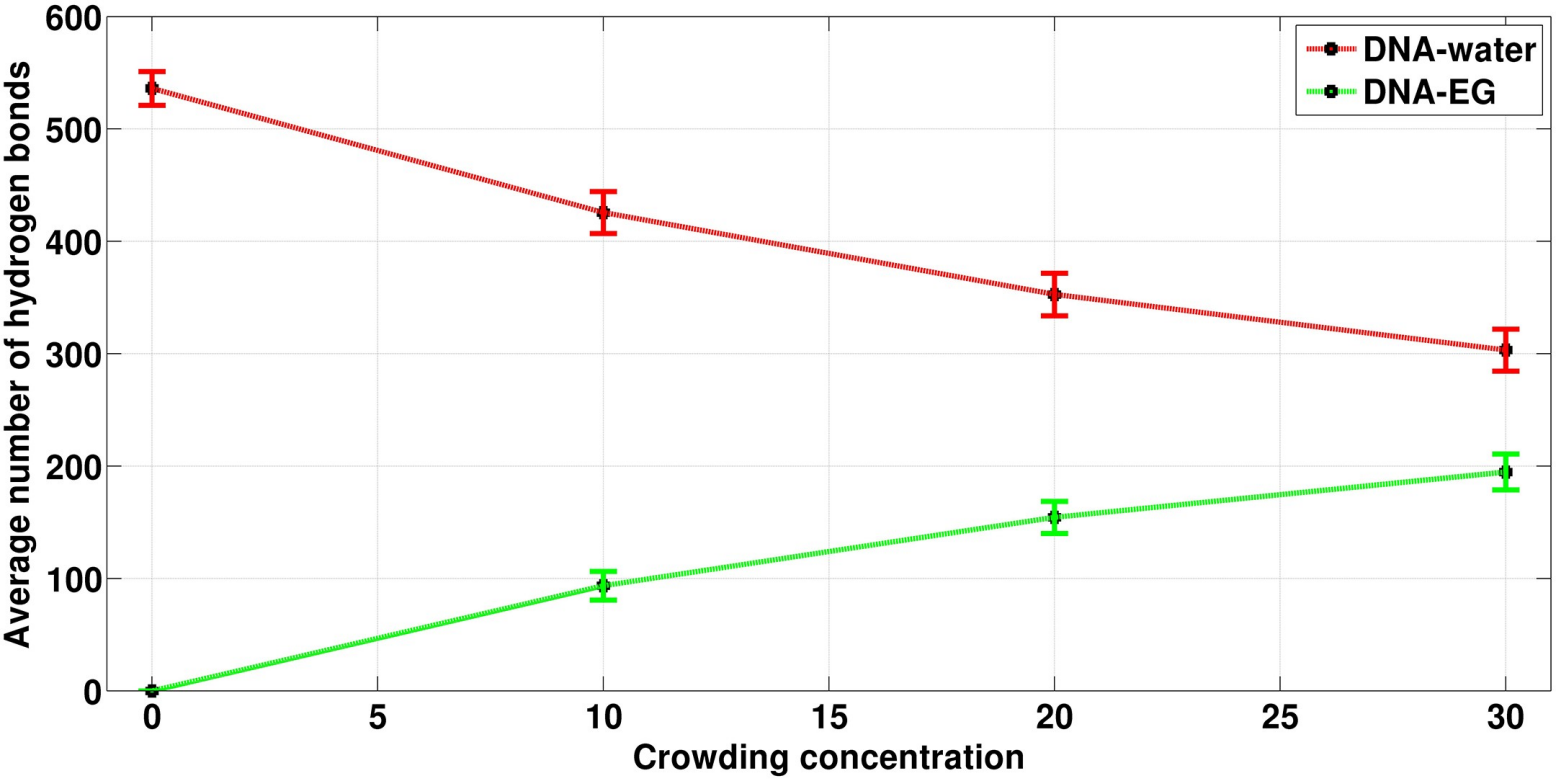
The average number of hydrogen bonds between DNA-EG and DNA-water. Red line corresponds to the number of hydrogen bonds between DNA-Water and green line corresponds to the number of hydrogen bonds between DNA-EG molecules. Crowding concentrations are given as percentage.

**Fig 10:**
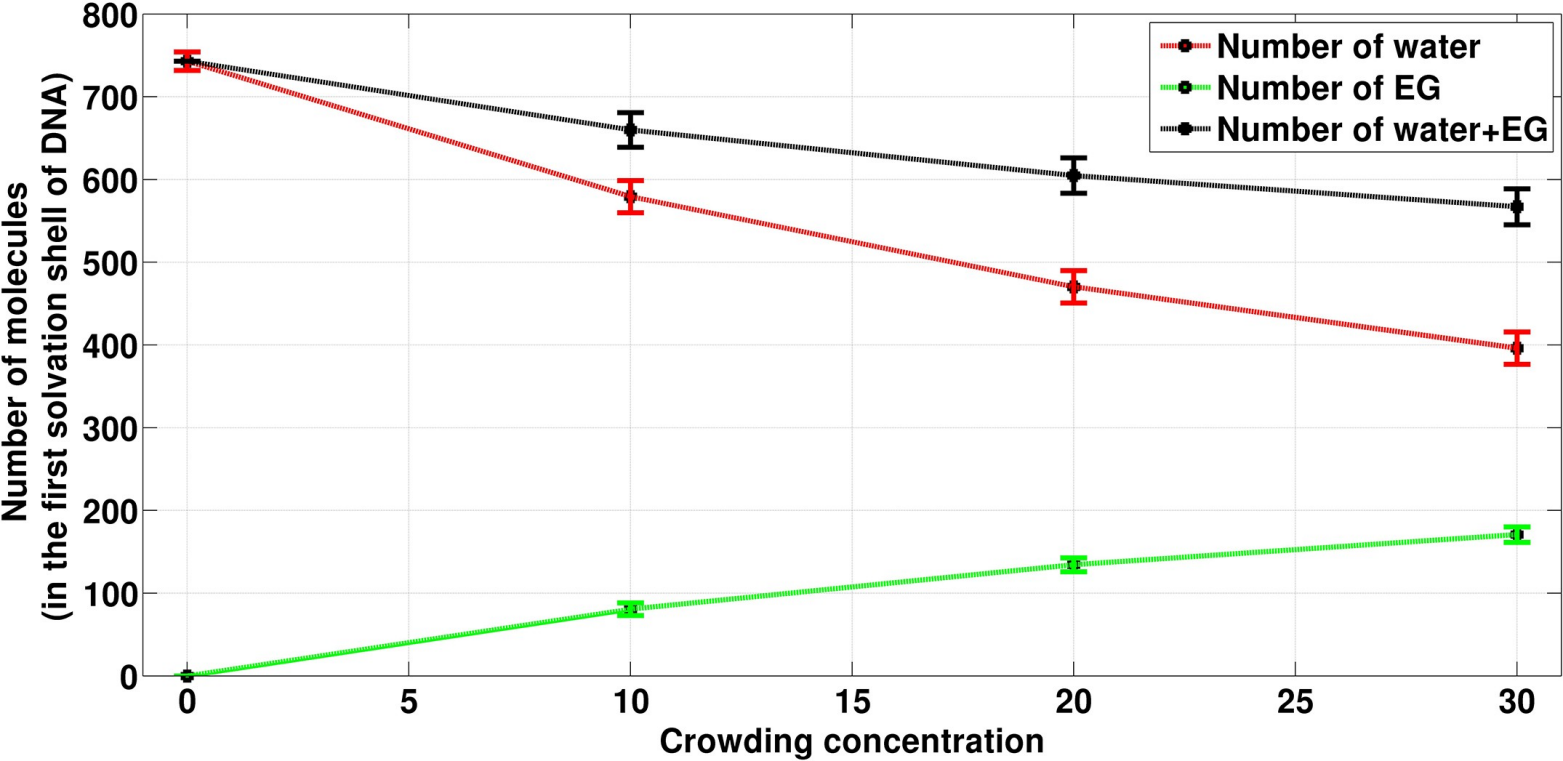
The average number of molecular species present in the first solvation shell of DNA (within 3.5 Å from DNA surface). Green, red and black lines correspond to the number of EG, number of water and number of EG + water present in first solvation shell of DNA. Crowding concentrations are given as percentage.

It is known that hydrogen bonds between water and DNA are one of the important reasons for DNA stability. Hence, we examined the variation of hydrogen bonds between water-DNA and EG-DNA as a function of EG concentration. All the hydrogen bonds between DNA-water and DNA-EG were calculated with a cutoff of donor-acceptor distance (D-A distance) of 3.5 Å and at a hydrogen bond angle (D-H-A angle) cutoff of 135°, respectively. From Fig 9, it can be seen that the number of hydrogen bonds between DNA and water is decreasing with the increase in crowder concentration (the average number of H-bonds is 553, 425, 352 and 303 corresponding to 0%, 10%, 20% and 30% crowding concentrations). On the other hand, the average number of hydrogen bonds between DNA and crowder was found to be 0, 93, 154 and 194 corresponding to 0%, 10%, 20% and 30% crowding concentrations, respectively. From this data, we can say (i) number of hydrogen bonds between DNA and water decreases, but increases for DNA and crowders as we increase the crowding concentration and (ii) the total number of hydrogen bonds involving DNA remain essentially same, *i*.*e*., the hydrogen bonds involving both water and crowder with DNA. However, it is clear that H-bonds coming from the crowders is not proportional to their increase in concentration (from 10% to 20% number of H-bonds increase is about 40%, while going from 20% to 30% it is around 20%), which implies that the displacement of waters from DNA is initially easy but becomes difficult as the crowder concentration increases. This was further verified by calculating the total number of water and crowder molecules in the first solvation shell as shown in Fig 10.

Fig 10 and Table 4 show that with the increase in concentration of crowder molecules, a continuous decrease in the average number of water molecules and a continuous increase in the average number of crowder molecules were observed in the first solvation shell of DNA (which is taken as 3.5 Å from the surface of the DNA). As crowding increases in the system from 0 to 10%, the decrease in the number of water molecules was found to be 169 while the increase of crowder molecules was 80. The number of water molecules decreased to 109 when crowding increased from 10% to 20%, which is smaller than the decrease in the number of water molecules when crowding increased from 0% to 10%. On further increasing the crowding concentration from 20% to 30%, the decrease in the number of water molecules (which is 74) was found to be further smaller as compared to the decrease in the number of water molecule decreased when crowding was increased from 10% to 20%. From Fig 10, it is also clear that the total species (water + crowder) count in the first solvation shell decreases with the increase in crowder concentration. This implies that one crowder molecule displaces more than one water molecules in the first solvation shell of DNA. As it can be seen from Table 4, a single EG molecule displaces approximately two water molecules from the first solvation shell of the DNA.

**Table 4:** The average number of water molecules replaced per EG molecule from the first solvation shell of DNA.

System	Average number of Water in first Solvation Shell	Average number of EG in first solvation Shell	Number of waters replaced per EG molecule in first solvation shell
0%	743.09 ± 11.19	-	-
10%	579.30 ± 19.43	80.65 ± 7.65	2.05
20%	470.32 ± 19.65	134.44 ± 8.40	2.03
30%	396.20 ± 19.64	170.87 ± 9.36	2.04

#### 2.4 Dynamics of water in the first solvation shell of DNA

**Fig 11:**
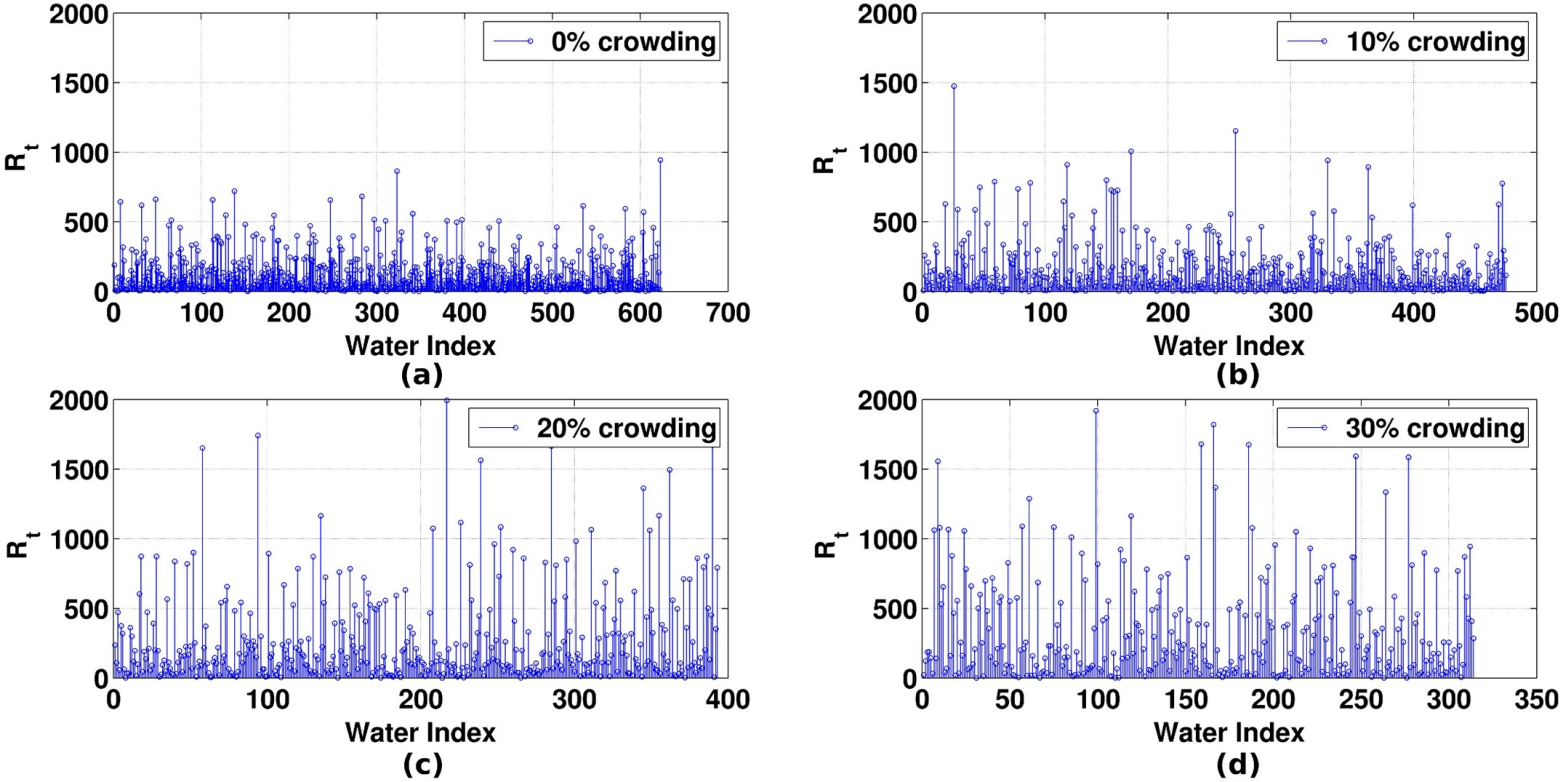
Residence time (R_t_ in picosecond) of water in the first hydration shell of DNA. (a) 0% crowding (b) 10% crowding (c) 20% crowding (d) 30% crowding.

Water molecules are dynamic in nature and the dynamics of the water molecules in close vicinity of the biomolecules can play an imperative role in the function and stability of the biomolecules [28,64,65]. In the earlier section, we have quantified the number of water molecules present in the first hydration shell of DNA for the corresponding four concentrations of EG and observed that the number of water molecules in the first hydration shell of DNA decreases with increasing crowding concentration. Further, we also showed that the number of hydrogen bonds formed between the DNA and water decreases with the increase in crowding concentration. Both these calculations provided more or less the overall picture of the solvent structure around DNA. However, the routes of dynamical relaxations of the water molecules were not captured. One of the quantities to capture the dynamics of water molecules is through the calculation of mean residence time (MRT), which estimates, on average, how long a water molecule stays around the biomolecule. It has been reported that the behavior of water molecules and water structure around proteins is quite regular. However, there is heterogeneity in the behavior of water molecules around DNA [66]. Different groups and studies have reported different residence time for the water molecules in the grooves of DNA which varies from 200 ps to 1 ns [67,68]. In case of DNA, which is a highly charged system, several factors govern the behavior of water molecules like topography (groove width and groove depth), the sequential arrangement of the bases and most importantly the chemistry of the nucleobases, water molecules and the surrounding environment. In our case, we are interested in capturing the change in residence time of water molecules as an effect of an increase in the crowding concentration in the system. MRT was calculated by taking the average of the residence time of each water molecule in the first hydration shell (3.5 Å from the DNA surface). For calculation of residence time, each water molecule in the first hydration shell was tracked for their presence and absence in the subsequent snapshots (for this calculation, the snapshots were saved at a gap of every 0.1 picosecond from a 5ns stretch of a MD trajectory). Fig 11 shows the residence time (in picosecond) of all the water molecules present in the first hydration shell of DNA. It can be clearly seen from the figure that the residence time of water molecules increases in the first hydration shell of DNA with increasing crowding concentration. For 0% crowding, the MRT of water molecule was found to be 140.94 ps. From the MRT plot (Figure W in S1 File), it can be seen that the MRT increases from 140.94 ps (0% crowding) to 330.10 ps (30% crowding) with the increase in the concentration of EG. Taken together, we found that the residence time of around 10.27% of water molecules for 0% crowding, 12.84% for 10% crowding, 25.44% for 20% crowding and 36.30% for 30% crowding is greater than the MRT of 30% crowded system (330.10 ps). This states that the water molecules in the first hydration shell are residing close to the DNA for longer timescales, *i*.*e*., the water molecules are getting strongly bounded to the DNA with the increase in crowding in the system. Overall, it can be concluded that with the increase in crowding concentration, the weakly bounded water molecules get easily displaced by EG molecules in the first hydration shell of DNA; however, the strongly bonded water molecules reside in the close vicinity of DNA, thus giving rise to a longer residence time.

To get a feeling for bulk water movement, diffusion coefficient of water for all four systems are calculated (Figure X in S1 File). The results clearly show the decrease of diffusion coefficient with the increase in EG concentration which implies the slow movement of water molecules in the presence of EG.

#### 2.5 Solvation shell around AT and GC base pairs

**Fig 12:**
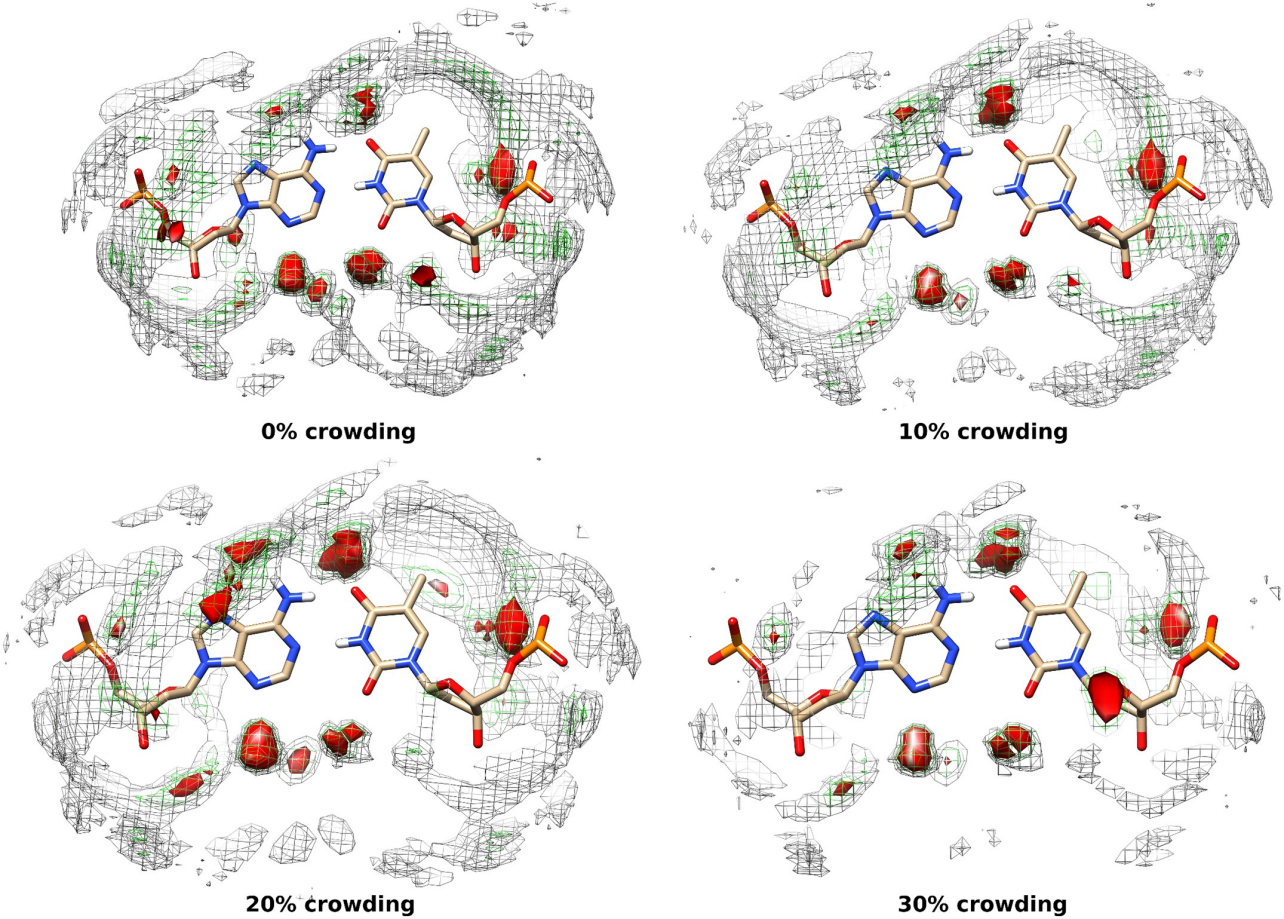
Water density around AT base pair at 0%, 10%, 20% and 30% crowding concentration. The isosurface densities (local number density) of water greater than 3.8 (gray mesh), 5.5 (green mesh) and 7.0 (red solid)are shown. The average structure of AT base pair is shown here.

**Fig 13:**
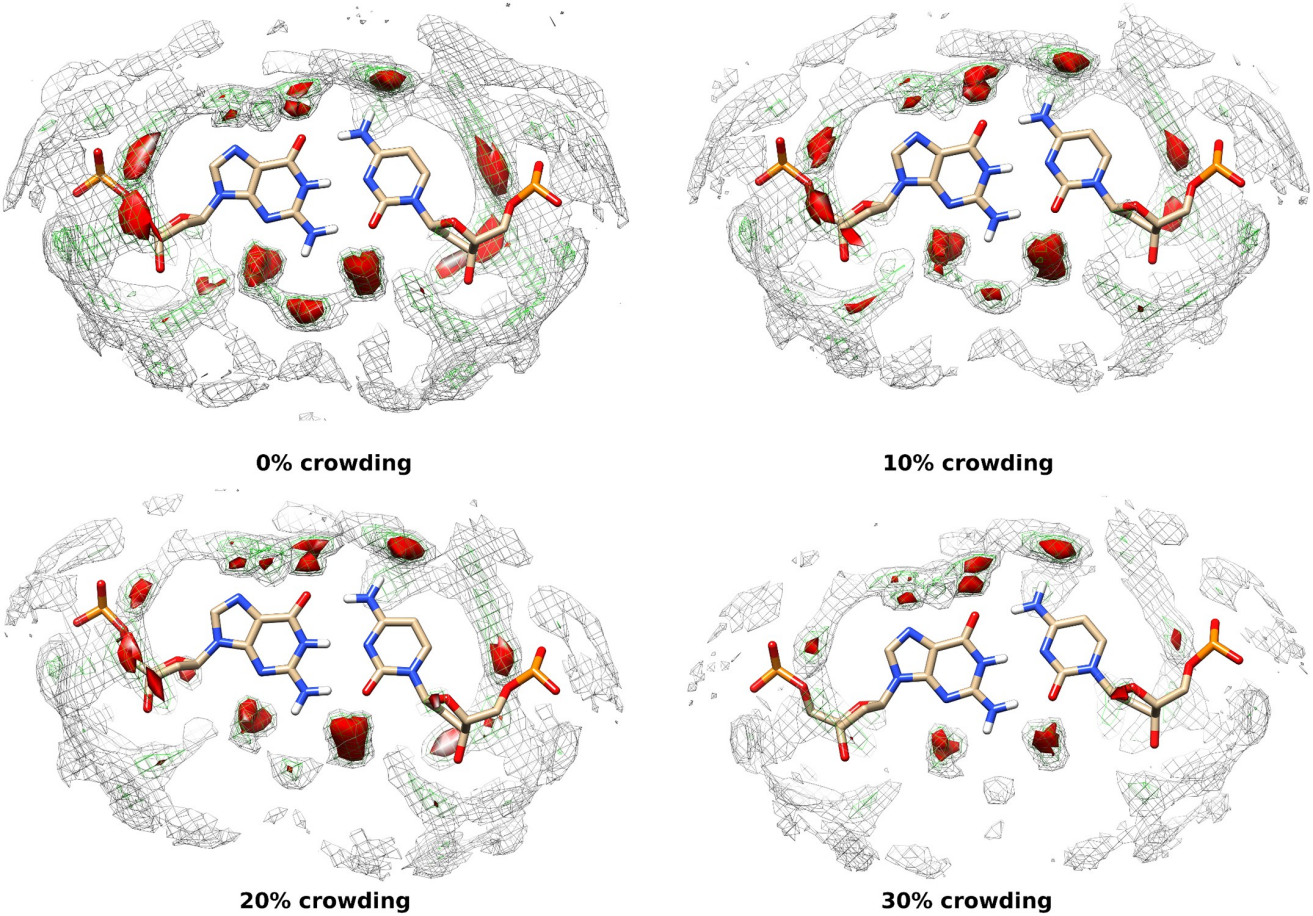
Water density around GC base pair at 0%, 10%, 20% and 30% crowding concentration. The isosurface densities (local number density) of water greater than 3.8 (gray mesh), 5.5 (green mesh) and 7.0 (red solid)are shown. The average structure of GC base pair is shown here.

We calculated the local density of water molecules around AT and GC base pairs (averages over MD trajectories) to understand the solvation structure around them. The local density of water molecules around the base pairs are shown in Figs 12 and 13. The isosurface water densities of 3.8 (gray colour), 5.5 (green colour) and, 7 (red colour) or more are shown in both the figures. As observed from the figures, the local density of water molecules decreases with increasing crowding concentration around the backbone atoms (OP1, OP2, P) of DNA which is consistent with the g(r) of water around DNA. The densities of water around the minor groove and major grooves of DNA are not much affected by increasing crowding concentrations. This suggest that crowder molecules are able to displace the water molecules around DNA backbone but replacement of water from major and minor groove of DNA is comparatively difficult.

#### 2.6 Effect of crowding on minor groove water spine

One of the most important factors in DNA solvation is minor groove water spine. It has been shown in the previously published works that water molecules bind strongly with minor groove atoms (N3, O2) and backbone phosphate group of DNA and hence have longer residence time around these atoms [69,70]. This strong interaction of water molecules with O2 of pyrimidine and N3 of purine in the minor groove of DNA helps in the formation of minor groove water spine. In a new study [71], with the help of nonlinear vibrational spectroscopy of DNA, it has been shown that water molecules in the minor groove of DNA form a chiral superstructure and hence form a chiral spine of solvation in the minor groove. The biological importance of the chiral spine of water is still not known, but it indicates that the minor groove spine waters are much different from other water molecules present in the system.

DNA minor groove spine structure has been extensively studied but how crowding affects this minor groove spine is still to be explored. To quantify the water spine of DNA minor groove, we have calculated the number of water molecules within 3.5 Å of N3, N2, H21, H22, O2 (atom definitions can be seen in Fig 1(b) and 1(c)) atoms of A, T, G and C with the increasing crowding concentration, as shown in Table 5. Since EG (crowder) molecule has two −OH groups, there will be a continuous competition between water and crowder to make hydrogen bonds with minor groove DNA atoms of AT and GC base pairs. We found that the average number of water molecules in the minor groove water spine decreases as the crowding concentration was increased. And, the average number of crowder molecules intruding into the minor groove water spine increases with increasing crowding concentration. As can be seen from Table 4, a single EG molecule replaces almost two water molecules from DNA solvation shell in the crowded environment, but in the case of minor groove water spine, almost 1.5–1.6 (Table 5) water molecules are being replaced by a single crowder molecule. This is possibly because of the narrow and deep structure of minor groove of B-DNA which makes it relatively difficult for crowder molecules to displace strongly bonded water molecules from the minor groove of DNA.

**Table 5:** The average number of water molecules replaced per EG molecule from the water spine of DNA minor groove.

System	Average number of water in minor groove water spine	Average number of crowder in minor groove water spine	Number of water replaced per EG molecule in first solvation shell
0%	70.03 ± 3.98	-	-
10%	52.92 ± 5.53	11.18 ± 2.70	1.53
20%	44.52 ± 5.68	15.89 ± 2.97	1.60
30%	39.55 ± 4.68	18.69 ± 2.62	1.63

**Fig 14:**
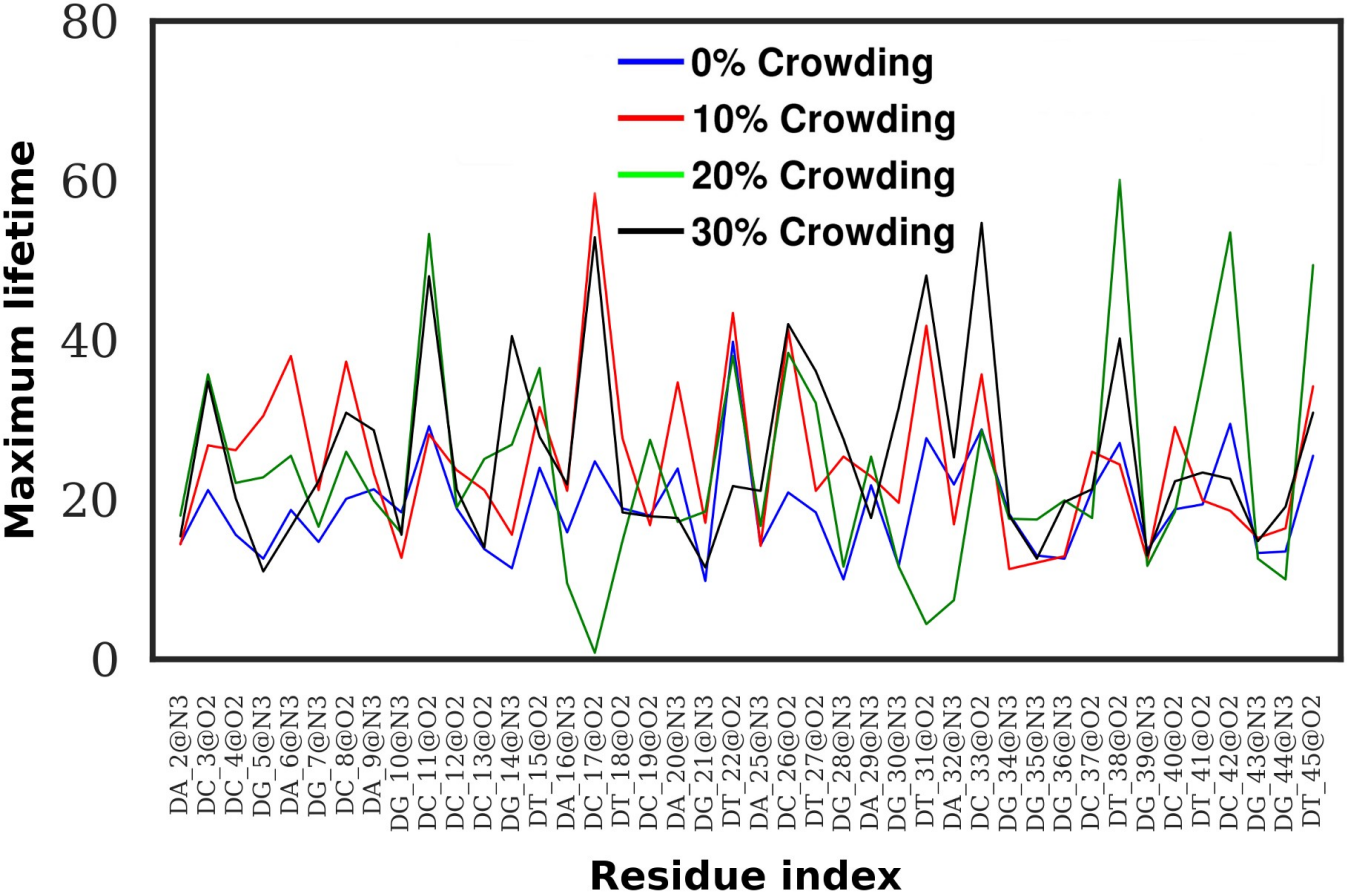
Maximum lifetime (in picosecond) of hydrogen bonds between DNA minor groove atoms and water at 0% (blue), 10% (red), 20% (green) and 30% (black) crowding concentration.

We further calculated the hydrogen bond lifetime of each hydrogen bond between the minor groove atoms and water molecules. Fig 14 shows the maximum lifetime of hydrogen bond formed by water with the minor groove atoms of DNA. It can be seen from the figure that except for two regions, the maximum lifetime of hydrogen bonds is higher in the crowded systems as compared to the system at 0% crowding. The average maximum lifetime (in picoseconds) was found to be 19.20 ± 6.32, 24.80 ± 10.34, 23.56 ± 13.43, and 25.50 ± 11.48 for 0%, 10%, 20%, and 30% crowding concentrations, respectively. It should also be noticed that the increase in the hydrogen bond lifetime is not monotonous with the increase in the crowding concentration.

### 3. Solvent-solvent properties

#### 3.1 Radial distribution functions and coordination number of water-water, water-EG, and EG-EG

**Fig 15:**
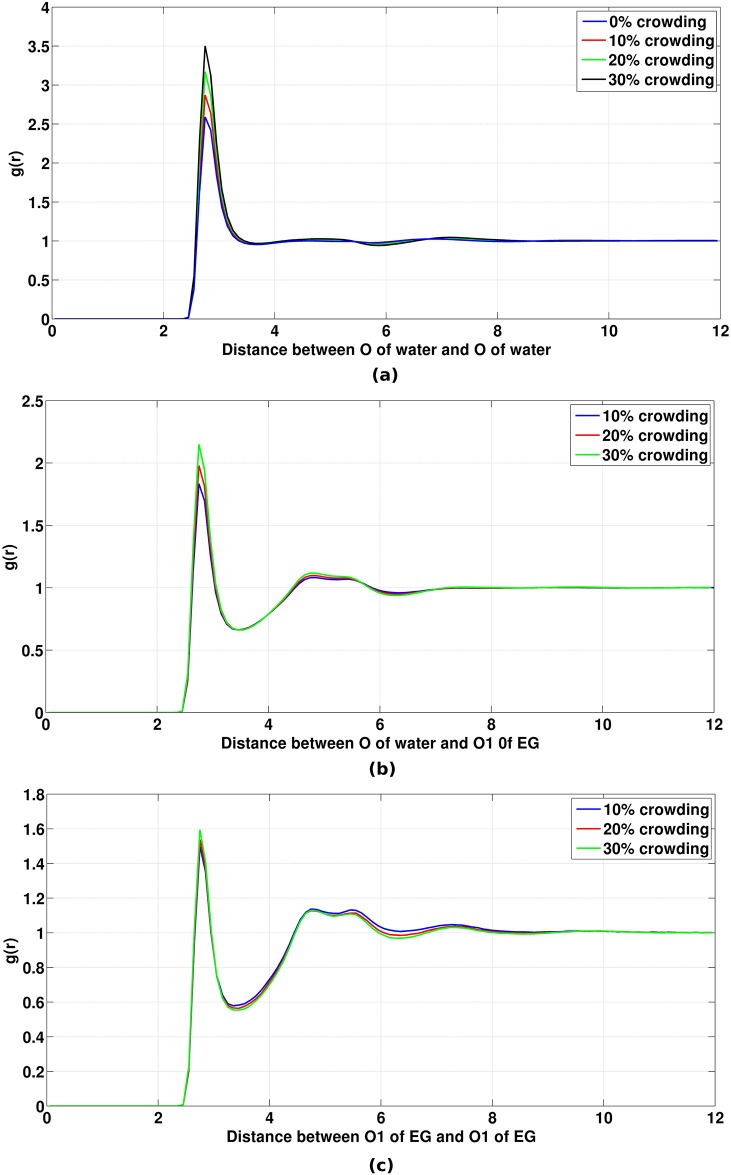
Pair correlation function of (a) oxygen of water and oxygen of water, (b) oxygen of crowder and oxygen of water (c) oxygen of crowder and oxygen of crowder. All distances are in Å.

In this section, we have tried to understand that how the structure of the solvent changes as crowding increases in the system. For this, PCFs of water-water, crowder-water and crowder-crowder were calculated at different crowding concentrations (Fig 15). Coordination numbers of first solvation shell of water around water, water around crowder and crowder around crowder were also calculated from the corresponding PCFs in different crowding concentrations which is shown in Table 6. We see that the first peak heights of all PCFs increase as crowding increases in the system. However, as can be seen from Table 6 that the coordination number of water around water and water around crowder decrease with the increase of crowder concentration. The coordination number of crowder around crowder increases with the increase of crowder concentration. This is in accord with the findings reported in the previous sections.

**Table 6:** The coordination number of oxygen of water around oxygen of water, oxygen of water around oxygen of crowder and oxygen of crowder around oxygen of crowder.

System	O of water around O of water	O of water around O of crowder	O of crowder around O of crowder
0%	6.04	-	-
10%	5.34	6.94	0.72
20%	4.70	6.00	1.32
30%	4.08	5.14	1.92

**Note**: The coordination number has been calculated by integrating g(r) till the first minimum after the first peak.

#### 3.2 Hydrogen bonds between water-water, water-EG, and EG-EG at different crowding concentrations in the whole system

Oxygen of water has a natural tendency to form hydrogen bond with hydrogen attached to an electronegative atom and hydrogen of water has the same tendency to form hydrogen bond with other electronegative atoms. Ethylene glycol on the other hand also has two −OH groups which can form hydrogen bonds with water and DNA. Water and ethylene glycol both can be hydrogen bond donor or acceptor in the formation of hydrogen bond. There could be four types of bonds involving water and EG, namely water-water (WW), water(donor)-EG(acceptor) (WE), EG(donor)-water(acceptor) (EW), and EG-EG (EE).

**Fig 16:**
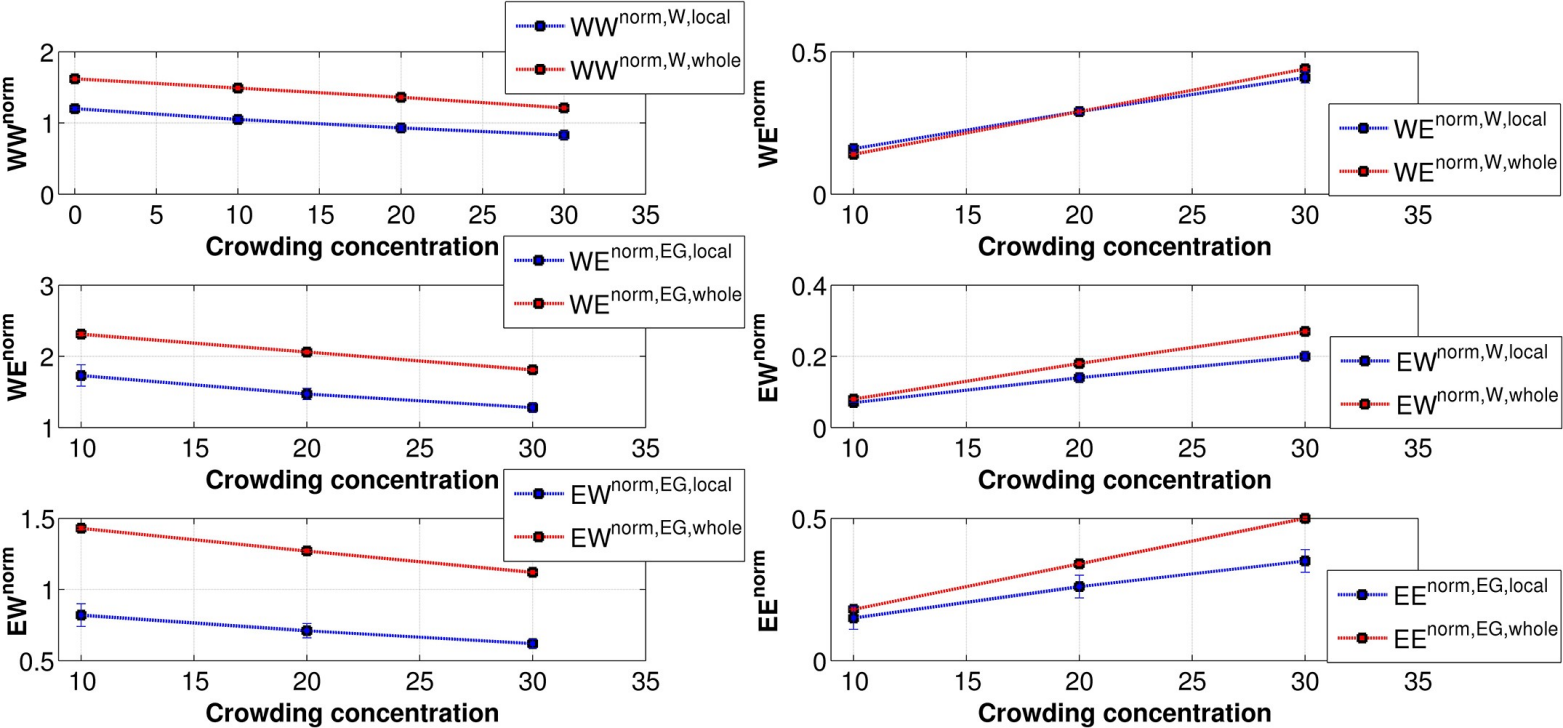
The average number of hydrogen bonds per species in the local vicinity of DNA (within 7 Å from the surface of DNA) and in the whole system. WW^norm^, WE^norm^, EW^norm^, and EE^norm^ represent the normalized number of hydrogen bonds between water-water, water-EG, EG-water, and EG-EG, respectively. In the legends, superscript {norm, W, local} represents normalization by total number of water molecules in the local region (within 7 Å from the surface of DNA), {norm, EG, local} represents normalization by total number of EG molecules in the local region, {norm, W, whole} represents normalization by total number of water molecules in the complete system and {norm, EG, whole} represents normalization by total number of EG in the complete system.

Upon examination of WW H-bonds (Fig 16), we see that number of H-bonds per water in the local region (within 7 Å from the surface of DNA) is less than that in bulk for all concentrations of crowder. Even when there is no crowder, this is true. This is partly because the first layer of water is forming H-bond with DNA and hence form lesser H-bonds with other waters. For the EE H-bonds at 10% crowding concentration, the number of H-bonds per EG is essentially same in the vicinity of DNA and in the bulk. However, at higher crowder concentrations, the EG in bulk make more H-bonds with other EGs. The trend monotonically increases with the increase in crowder concentration. This suggests that there is an optimum number of EGs, which can be accommodated near DNA by replacing water. From the number of H-bonds per EG, it appears that between two EGs there are water molecules. These are also reflected in the PCF of EG-EG (Fig 15(c)) as the first peak height is less than both water-water and water-EG PCFs. However, replacing waters become more difficult with an increase of crowder concentration as discussed previously.

For WE and EW H-bonds, the number of bonds per water is essentially same for local region and bulk. However, the number of H-bonds per EG is much less in the local region. This is most likely because the number of hydrogen bonds involving one EG is more in bulk. The increase in the number of bonds comes from the increase in the number of water molecules around EG in bulk.

The total number of hydrogen bonds for WW, WE, EW, EE for both bulk and 7 Å from DNA is given in Tables B and C in S1 File. Hydrogen bond calculations for the complete system were found to be qualitatively consistent with the previously published works [72–74].

### 4. Localized thermodynamics of the crowded system

PCFs calculated among different species in the last sections give idea about their average interaction. −*kTlng*(*r*) (*k* and *T* are Boltzmann constant and absolute temperature, respectively) gives the potential of mean force (PMF) on the variable chosen (in our case it was distance between different pair of species) averaged over all other degrees of freedom of the system. In the current section, we looked at the localized properties of water near DNA. This gives another way to see the complex interactions happening in our system.

#### 4.1 Free energy per water molecule in the first solvation shell of DNA

We have used GIST, as implemented in the cpptraj module of Amber 14, to study the thermodynamic properties of water molecules present in the first solvation shell of 23 base pair DNA duplex. GIST grid size was taken as 0.5 Å and the local region (R) was defined in such a way that it covers the whole DNA with a margin of 4 Å from the surface of DNA. The box size for GIST calculation around DNA has dimensions 28 Å × 28 Å × 84 Å which represents the first solvation shell around DNA. As GIST does not allow treating other molecules except water as part of solvent, crowder and salt ions were removed from the trajectories before the GIST calculation. Bulk number density of water *ρ*^0^ (required for calculating solvation entropy term) and mean water-water interaction energy $E_{ww}^{0}$ (required for referencing water-water interaction energy), which are required for the GIST calculation, were obtained from TIP3P water box simulation at 300 K as 0.0334 molecules/Å^3^ and -9.565 kcal/mol, respectively.

We analyzed the translational and orientational entropy of water, water-DNA interaction potential and water-water interaction potential calculated *via* GIST for their convergence as a function of simulation time (Figure Y in S1 File). We found that the translational entropy, solute-solvent interaction energy *ΔE*_*sw*_ and unreferenced (water-water interaction potential is not referenced to bulk water) water-water interaction energy *E*_*ww*_ got converged well, but we could not get convergence for orientational entropy $\Delta S_{sw}^{orient}$ for the performed simulation length. One of the reasons for not getting converged orientational entropy could be that many important orientational degrees of freedom of water molecules could not be explored enough during the simulation length. The orientational entropy of water did not converge by extending the simulation till 150 ns which have also been reported by Nakano *et al*. [28]. It was found that the entropy values are one order of magnitude less than the enthalpy values as shown in Table 7. Hence, the lack of convergence of orientational entropy is not going to affect the conclusions made in the current section.

**Table 7:** Normalized thermodynamic quantities of water molecules calculated using GIST for the region ‘R’.

System	*T*Δ*S*^*trans*,*R*,*norm*^	$\Delta E_{sw}^{R,norm}$	$\Delta E_{ww}^{R,norm}$	$\Delta G^{R,norm}$
0%	-23.13 (± 3.62) × 10^−2^	-407.60 (± 1.19) × 10^−2^	254.69 (± 0.98) × 10^−2^	-129.78 (± 1.87) × 10^−2^
10%	-7.75 (± 0.34) × 10^−2^	-422.46 (± 4.76) × 10^−2^	334.26 (± 1.71) × 10^−2^	-80.45 (± 5.68) × 10^−2^
20%	3.19 (± 0.01) × 10^−2^	-430.85 (± 1.02) × 10^−2^	397.61 (± 2.56) × 10^−2^	-36.43 (± 3.61) × 10^−2^
30%	13.09 (± 0.15) × 10^−2^	-448.75 (± 8.22) × 10^−2^	463.86 (± 5.66) × 10^−2^	2.02 (± 13.40) × 10^−2^

**Note**: All values in kcal/mol/water.

Region ‘R’ represents the first solvation shell around DNA.

**Fig 17:**
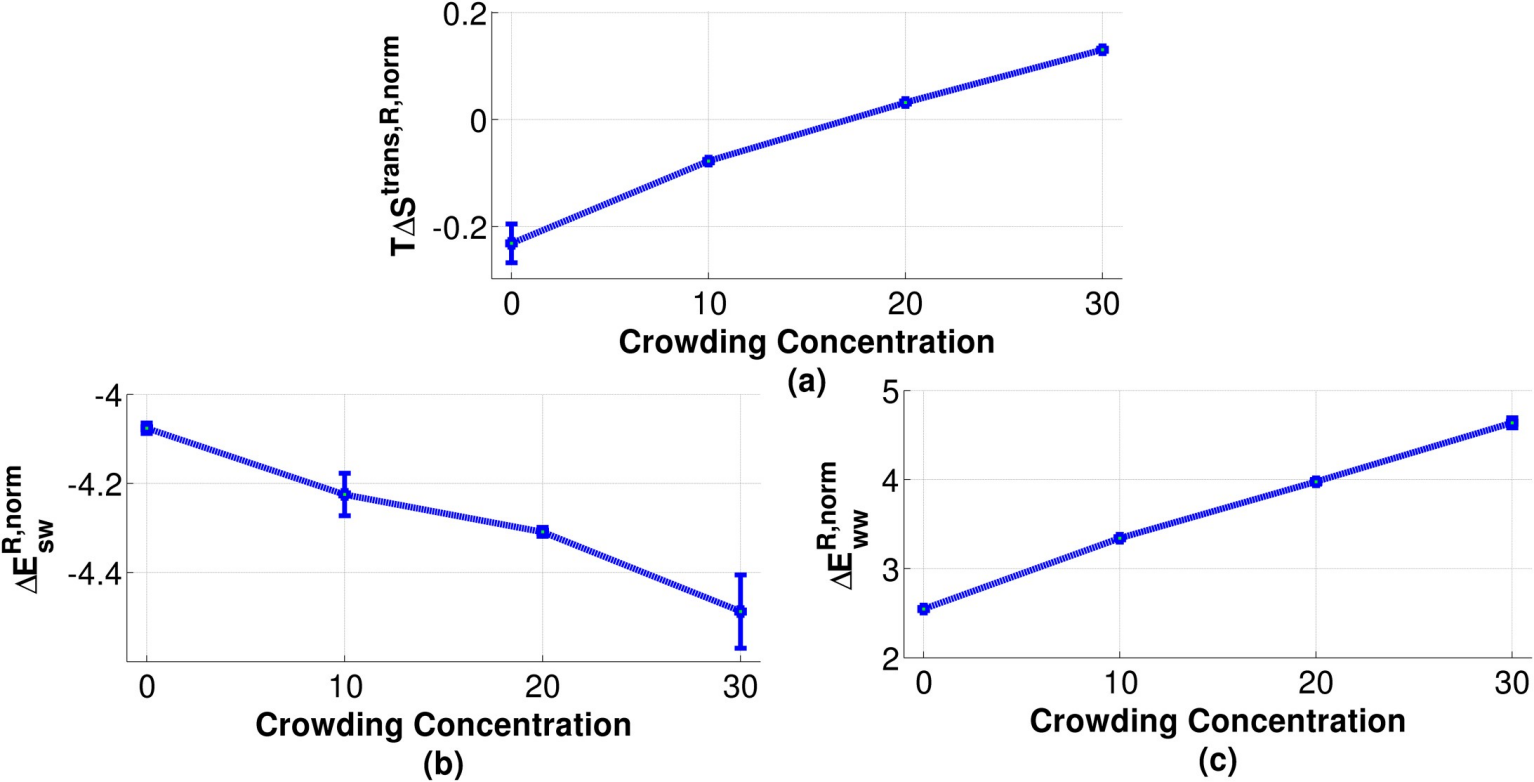
Thermodynamic properties calculated from GIST in kcal/mol. (a) Referenced normalized translational entropy (b) referenced normalized water-DNA interaction potential with and (c) referenced normalized water-water interaction potential with respect to bulk water at different crowding concentration.

Since orientational entropy per water has not been found to converge in any of the systems we have not included the orientational entropy for free energy change per water calculations. We can still say the following: As delineated in Fig 17(a) it can be said that translational entropy per water molecule increases with increasing crowding concentration. This suggests that water molecules can explore more translational degrees of freedom as crowding increases in the system which might be because of a lesser number of water molecules in the first solvation shell of DNA.

The solute-solvent interaction potential per water has been illustrated in Fig 17(b). It has been found that with increasing crowding concentration $\Delta E_{sw}^{R,norm}$ continuously decreases which is consistent with the previous published results [28]. This suggests that with increasing crowding concentration weakly interacting water molecules are being replaced by crowder molecules. As a result only strongly bounded water molecules are being able to interact with DNA, thus lowering solute-solvent interaction potential as crowding concentration increases. From Fig 17(c) it can be seen that with increasing crowding concentration $\Delta E_{ww}^{R,norm}$ per water increases. This is because of intrusion by crowder molecules in region R disrupting water-water interactions.

**Fig 18:**
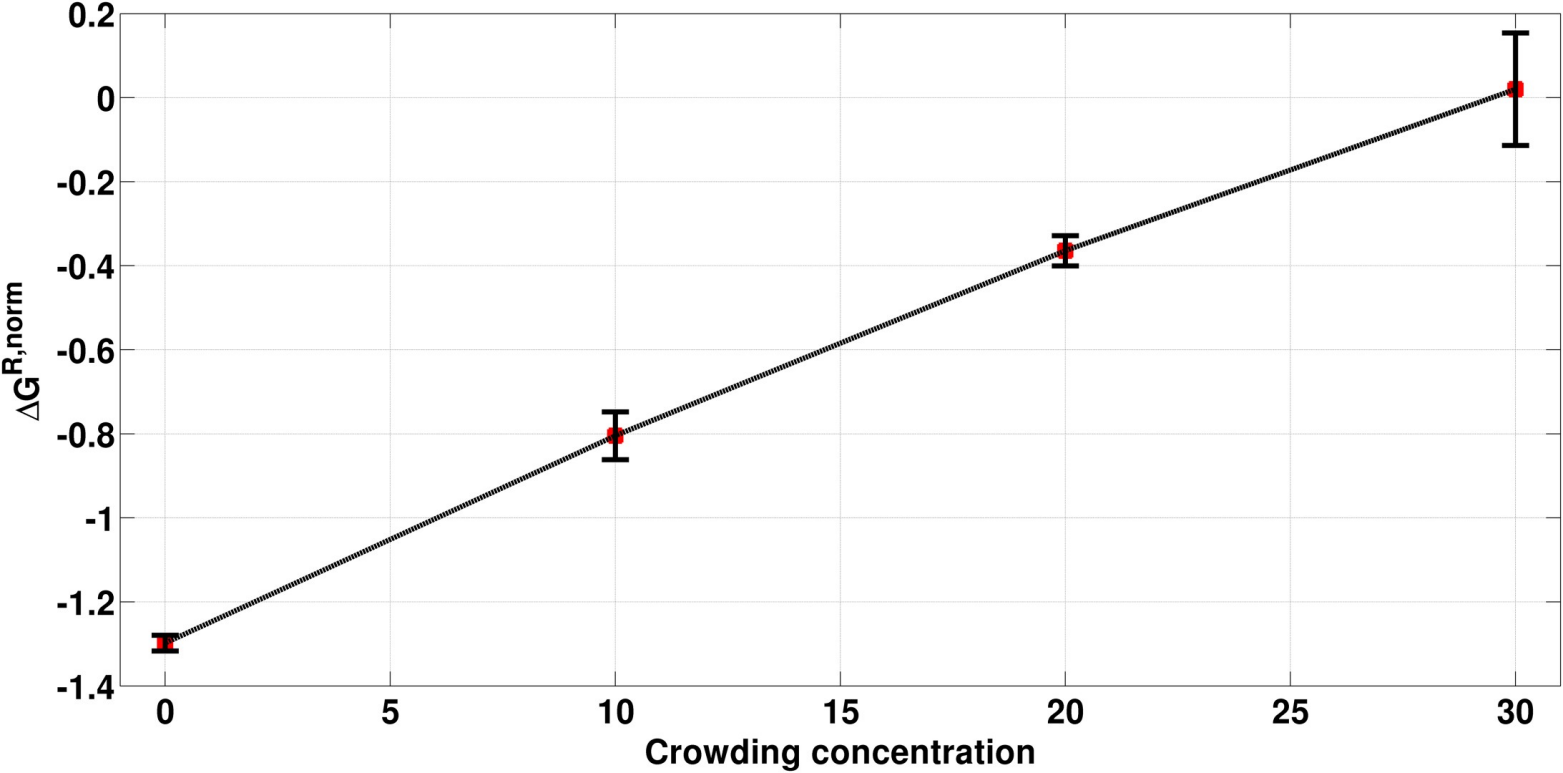
Free energy change per water molecule in kcal/mol with reference to bulk water at different crowding concentration (given in percentage).

As delineated in Fig 18, the free energy per water molecule increases with increasing crowding concentration. It is to be noted that the enthalpy interaction with crowders is not taken into account. If we look at the total number of H-bonds in the local region (within 7 Å from DNA surface, Table C in S1 File), it can be concluded that the numbers are increasing for water-crowder case. Hence, it is likely that the loss of stability per water might be, at least, partially compensated by the favorable water-EG H-bonds.

#### 4.2 Binding free energy per crowder molecule to DNA

As GIST method currently works for water only, to calculate the approximate binding free energy per crowder molecule, we have used molecular mechanics–three-Dimensional Reference Interaction Site Model (MM-3DRISM). The following formula has been used to calculate approximate binding free energy per crowder molecule to DNA:

$$\Delta G_{Binding}^{DNA-EG,norm}=\langle \frac{\Delta G_{Binding}^{DNA-EG}}{N_{C}}\rangle$$

where, *N*_*C*_ is the number of crowder molecules lying within the margin of 15 Å from the surface of DNA (this is because DNA-crowder correlations get lost beyond 15 Å distance which is also visible in g(r) of crowder around DNA (Fig 5(b)), the angular brackets represent average taken over 50 snapshots of the MD simulation and

$$\Delta G_{Binding}^{DNA-EG}=(E_{}^{DNA-EG}+\Delta G_{Solvation}^{DNA-EG})-(E_{}^{DNA}+\Delta G_{Solvation}^{DNA})-(E_{}^{EG}+\Delta G_{Solvation}^{EG})$$

where, *E* represents the solute potential energy calculated using MM forcefield, $\Delta G_{Solvation}^{DNA-EG}$ represents solvation free energy of DNA-EG complex, $\Delta G_{Solvation}^{DNA}$ represents solvation free energy of DNA and $\Delta G_{Solvation}^{EG}$ represents solvation free energy of crowder calculated using 3DRISM.

The entropy of both DNA and all the crowders was not considered. As discussed in result section 1, that the DNA structural fluctuation is not much affected by EG concentration, neglect of entropy may not lead to large error. However, we cannot comment on the entropy of EG at different concentrations of EG. We expect that as entropy terms have much smaller magnitude, the qualitative trend of the calculated values using RISM may be reliable even without the EG entropy.

The binding free energy per crowder at 10%, 20%, and 30% crowding concentrations was found to be -0.57 ± 0.11, -0.56 ± 0.06 and -0.51 ± 0.06 kcal/mol. Ignoring the marginal increase in binding free energy per crowder, we can say that the stability of a crowder, on average, does not change significantly with the increase in crowder concentration.

## Discussion

One of the important objectives of equilibrium statistical mechanics is to know the equilibrium distribution of different components in a multi-component system. This could be an arrangement of counterions around macroions, the arrangement of water around hydrophilic and hydrophobic solutes, *etc*. The explanation of this kind of observation from the microscopic picture was explored in a large number of works [75–78]. However, as far as our knowledge goes, there were not many investigations, where distribution and thermodynamics of water, ions, and crowder around DNA have been investigated. We wanted to understand that how the distribution of different species changes upon changing the concentration of one species, namely the crowder, ethylene glycol molecule. And, what are the underlying interactions causing the changes is something we are trying to understand.

At first, we give some connection of the observations that came from the current work with the observations obtained in previous works. The effect of co-solute on the structural stability and dynamics of DNA was investigated in various previous works [27,79–84]. The major difference between those works and the current work is that in the present work both the local and global effects of crowding were investigated. It was found that EG intrudes into the solvation shell of DNA by replacing water, but it becomes more difficult as the concentration of EG increases. This means some waters are tightly bound to DNA and replacing those is not favored free energetically. It was found that the coordination number of water molecules decreases while that of crowder molecules increases around DNA with increasing the crowding concentration in the system. This is in accordance with the previous MD simulation and observations obtained from near-IR spectroscopy [72,73] for the pure water-EG mixture. Further, we observed that the loss in water coordination number was being compensated by gain in crowder coordination number, keeping total number of hydrogen bonds coming from water and crowder molecules in the vicinity of DNA almost constant. Among the various possibilities of H-bond, water-water H-bond was found to be maximum followed by the water-EG H-bonds and finally EG-EG H-bond for bulk solvent. This is in accordance with the earlier findings reported by Weng *et al*. [73] for water-EG mixtures. However, we have found that the number of EG-EG H-bonds is not negligible (reference [72] found that number is essentially negligible). This may be because of the changed electrostatics (due to the presence of DNA) of the system. Both mean residence time of waters in first solvation shell of DNA and H-bond lifetime in the minor groove of DNA gave insight to the complex dynamics of waters in different environment.

From the localized thermodynamics part, free energy per water in the first solvation shell increases with the increase in EG concentration. Entropy plays a less important role in the localized free energy of water. However, the free energy per crowder (within 15 Å from DNA) remains similar with the increase in EG concentration. It is to be noted that the localized free energy calculations have approximations. Hence, the values should not be taken quantitatively. However, the qualitative picture emerges out of these calculation gives insight into the nature of the first solvation shell.

The observations from this study can be summarized as—(a) EG disturbs the H-bonding network and electrostatics both near and far from DNA, (b) the number of H-bonds with DNA by the solvents remains essentially same (however, their nature changes as EG intrudes in the first solvation shell of DNA), (c) the pattern of H-bonding between water and EG near DNA differs from that of the bulk, (d) DNA dynamics and structural fluctuations are not significant, primarily because of the short length of the duplex, (e) approximate localized free energy per water and per crowder shows monotonic increase per water as EG concentration is increased (however, for crowder, it is essentially same), (f) MRT of water in the first solvation shell of DNA increases with the crowding concentration.

One important question is whether our observations are entropy driven and/or enthalpy driven? Essentially peaks of all PCFs are not broadened by the increase of crowder concentration. This indicates that the primary interaction is enthalpic. Entropy cost of arranging EG molecules near DNA at lower concentration is more than that at higher concentration [85]. However, even at 10% EG concentration, DNA-EG PCF shows a sharp first peak, indicating entropy cost has been compensated by the enthalpic effect (also by the entropy gain from the release of waters near DNA). DNA-Na^+^ PCF shows more fine structure at 30% EG than lower concentrations. It indicates strong macroion (here, DNA is the macroion)-counterion interaction in the presence of a binary solvent. Previous work has shown that both enthalpic and entropic effects are present in macroion-counterion interaction [86]. This is also likely to be happening here. As far as the PCFs with the ions are concerned, WAT-Cl^-^ (EG-Cl^-^) PCF is broader than WAT-Na^+^ PCF (EG-Na^+^). As Cl^-^ is far from DNA, it is not as tightly bound with either water or EG. Same is the reason for broadening of the second peak in the PCF of Na^+^ and Cl^-^. Hence, from the series of PCFs calculated, we can conclude that the first solvation shell effects are mostly governed by enthalpy. The same conclusion also comes from GIST calculation, where a different methodology was used to extract first solvation shell properties. We also comment that electrostatics in our system cannot be rationalized by a simple scaling of dielectric constant, because there is considerable heterogeneity of charge density near DNA, at least up to second solvation shell. An average effect of this is unlikely to be captured by a single dielectric constant.

Finally, we are left with a pertinent question regarding our observations and its dependence on the model used. The main drawback of our model is that it is non-polarizable, as typically forcefields of biomolecular simulations are. Hence, the charge density of one species does not change in response to the presence of another charged species. It is likely that the first solvation shell strong interactions will remain strong even with a polarizable potential. The main change may happen in the region a bit far from DNA. It is to be noted that H-bonding can be highly cooperative. However, in the non-polarizable model used in the current work, the cooperativeness is not properly captured. This may change some of the conclusions made in the current work.

## Conclusion

This work aimed to understand the effect of crowding on structure and thermodynamics of DNA solvation at the molecular level in an EG plus water solvent. Our results show that there is an interesting and non-trivial competition involving EG, water and salt ions, which is mainly governed by complex electrostatics and hydrogen bonding. Our results mostly match with experiment and previous simulations done with pure EG plus water mixture for hydrogen bonding pattern. Our findings suggest doing experiments that can capture the structural and thermodynamics of localized region near DNA (such as using two-dimensional near Infra-red (2D-NIR) techniques). This work can be extended (a) using polarizable forcefield, which should capture the H-bonding more quantitatively, and (b) by using it to study the effect of DNA sequence.

## Supporting information

S1 File This file contains all the supplementary text, figures and tables. (DOCX)
