# Supplementary material for: Microscopic picture of water-ethylene glycol interaction near a model DNA by computer simulation: Concentration dependence, structure, and localized thermodynamics
Source: PLoS One. 2018 Nov 14;13(11):e0206359. doi: 10.1371/journal.pone.0206359 (PMC6235303; doi:10.1371/journal.pone.0206359)
Supplement: S1 File — (DOCX) [file pone.0206359.s001.docx]

**Microscopic picture of water-ethylene glycol interaction near a model DNA by computer simulation: concentration dependence, structure, and localized thermodynamics**

Atul Kumar Jaiswal, Rakesh Srivastava, Preeti Pandey, Pradipta Bandyopadhyay*

School of Computational and Integrative Sciences, Jawaharlal Nehru University, New Delhi, INDIA

Atul KumarJaiswal - Email: [atulj91@gmail.com](mailto:atulj91@gmail.com)

Rakesh Srivastava - Email: [allahabad.21@gmail.com](mailto:allahabad.21@gmail.com)

Preeti Pandey - Email: [preetipandey2806@gmail.com](mailto:preetipandey2806@gmail.com)

Pradipta Bandyopadhyay - Email: [praban07@gmail.com](mailto:praban07@gmail.com)

**Supplementary Material**

**1: Relative comparison of ff99bsc0 and ff99parmbsc1 forcefields for DNA**

We have used an older version of DNA forcefield (ff99bsc0) for our simulations; however, currently a newer forcefield for DNA (ff99parmbsc1) [1] is available. Hence, to understand that whether the quantities computed in this study are forcefield dependent or not, we compared both the structural and thermodynamic properties of DNA plus water system using both the forcefields. We examined the differences in various DNA structure and solvent properties like RMSD, RMSF, end to end distance, bending angle of DNA and pair correlation function (PCF) of water around DNA backbone. From Figure A(a), it can be seen that the RMSD of DNA with respect to initial structure shows higher fluctuation using ff99bsc0 as compared to ff99parmbsc1. The average RMSD obtained using ff99bsc0 and ff99parmbsc1 were 5.61 ± 0.58 and 3.15 ± 0.70 Å, respectively. The RMSF values (Figure A(b)) using ff99bsc0 were observed to be slightly higher than ff99parmbsc1. The calculated mean and standard deviation of end to end distance (Figure B) (calculated by measuring the distance between center of mass of first (base-pair 1 and 46) to the last base-pair (base-pair 23 and 24)) was found to be 70.33 ± 1.94 Å (from ff99bsc0) and 71.68 ± 1.38 Å (from ff99parmbsc1); which is essentially similar. We also examined the differences in the DNA bending angle (Figure C) calculated using both the forcefields. The bending angle was calculated as the absolute angle between center of mass of first base pair (1 and 46), twelfth base-pair (12 and 35) and last base pair (23 and 24). The calculated mean and standard deviation of DNA bending angle was found to be 164.09 ± 8.64 degree (from ff99bsc0) and 166.66 ± 7.41 degrees (from ff99parmbsc1). Both, the end to end distance and DNA bending angle did not show any significant difference using the two forcefields. Another major property which we calculated was pair correlation function of water around DNA (Figure D), which also did not show any significant difference in their values. Combining all these results (Figures A, B, C and D) together we can say that the choice of forcefield does not alter our results except RMSD of the DNA. As the main emphasis of this paper was the water (and crowder)-DNA interaction in terms of their PCF (which is related to the potential of mean force), we believe our results will be essentially insensitive to the choice of different (recent) AMBER forcefields.

**Supplementary Tables:**

**Table A. RESP Charge on EG atoms.**

| **Atom** | **Atom type** | **Charge** |
| --- | --- | --- |
| O1 | oh | -0.6947 |
| H1 | ho | 0.4192 |
| C2 | c3 | 0.2177 |
| H21 | hc | 0.0289 |
| H22 | hc | 0.0289 |
| C3 | c3 | 0.2177 |
| H31 | hc | 0.0289 |
| H32 | hc | 0.0289 |
| O4 | oh | -0.6947 |
| H4 | ho | 0.4192 |

**Table B. Total number of hydrogen bonds for WW, WE, EW and EE for the complete system.**

| **System** | **WW** | **WE** | **EW** | **EE** |
| --- | --- | --- | --- | --- |
| 0% | .57 x 10^5^ ± .98 x 10^2^ | -- | -- | -- |
| 10% | .47 x 10^5^ ± .99 x 10^2^ | .46 x 10^4^ ± .43 x 10^2^ | .28 x 10^4^ ± .30 x 10^2^ | .37 x 10^3^ ± .19 x 10^2^ |
| 20% | .38 x 10^5^ ± .93 x 10^2^ | .82 x 10^4^ ± .60 x 10^2^ | .51 x 10^4^ ± .44 x 10^2^ | .14 x 10^4^ ± .36 x 10^2^ |
| 30% | .30 x 10^5^ ± .96 x 10^2^ | .11 x 10^5^ ± .68 x 10^2^ | .68 x 10^4^ ± .53 x 10^2^ | .30 x 10^4^ ± .51 x 10^2^ |

**Table C. Total number of hydrogen bonds for WW, WE, EW and EE within 7 Å from DNA.**

| **System** | **WW** | **WE** | **EW** | **EE** |
| --- | --- | --- | --- | --- |
| 0% | .23 x 10^4^ ± .41 x 10^2^ |  |  |  |
| 10% | .16 x 10^4^ ± .62 x 10^2^ | .25 x 10^3^ ± .22 x 10^2^ | .12 x 10^3^ ± .11 x 10^2^ | .22 x 10^2^ ± .06 x 10^2^ |
| 20% | .12 x 10^4^ ± .59 x 10^2^ | .38 x 10^3^ ± .22 x 10^2^ | .18 x 10^3^ ± .13 x 10^2^ | .69 x 10^2^ ± .11 x 10^2^ |
| 30% | .90 x 10^3^ ± .57 x 10^2^ | .44 x 10^3^ ± .21 x 10^2^ | .21 x 10^3^ ± .13 x 10^2^ | .12 x 10^3^ ± .15 x 10^2^ |

**Supplementary Figures:**

| **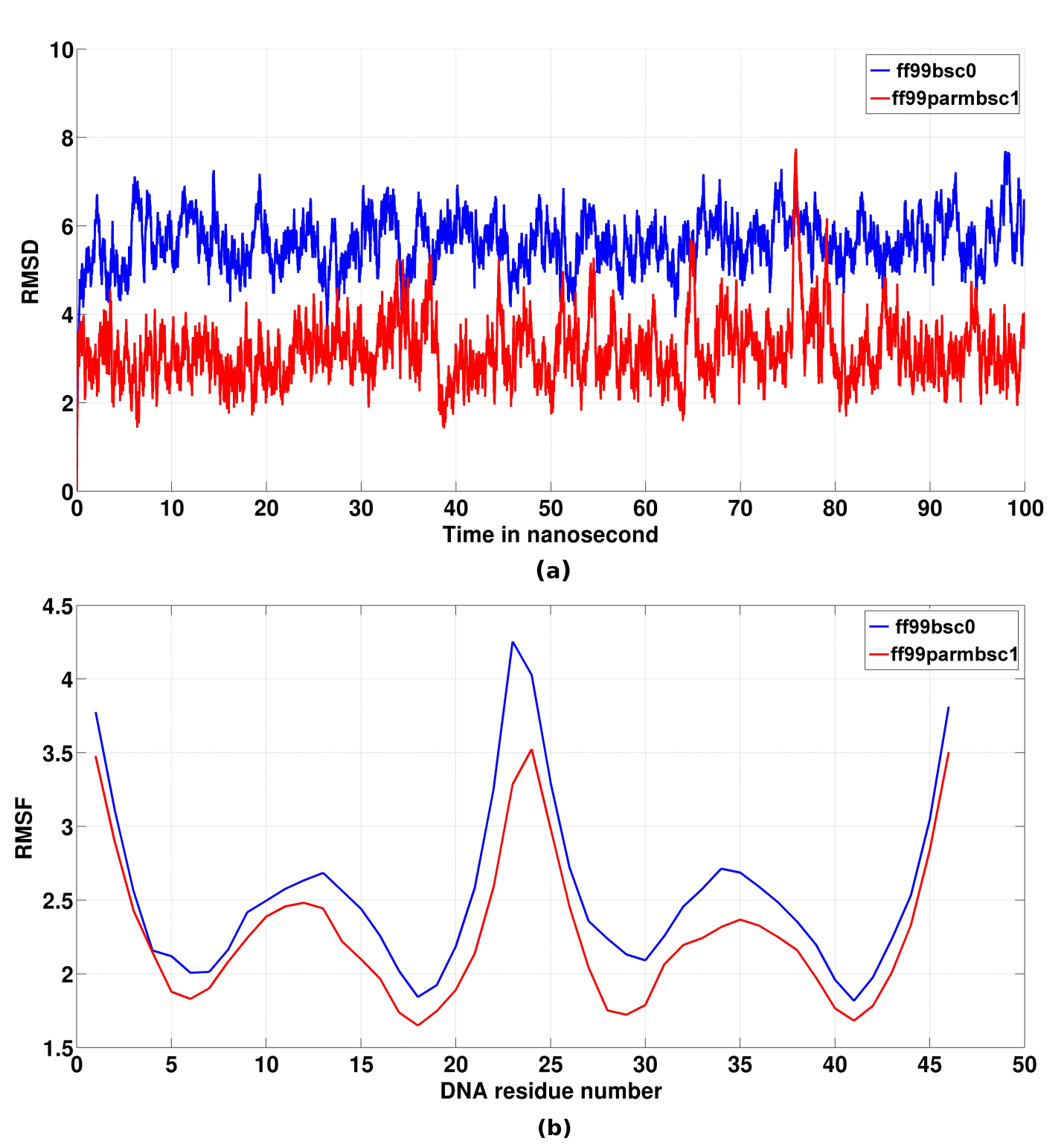** |
| --- |

**Fig A: Comparison of RMSD and RMSF (in Å) of DNA structure using ff99bsc0 and ff99parmbsc1 forcefields for DNA plus water system.**

| **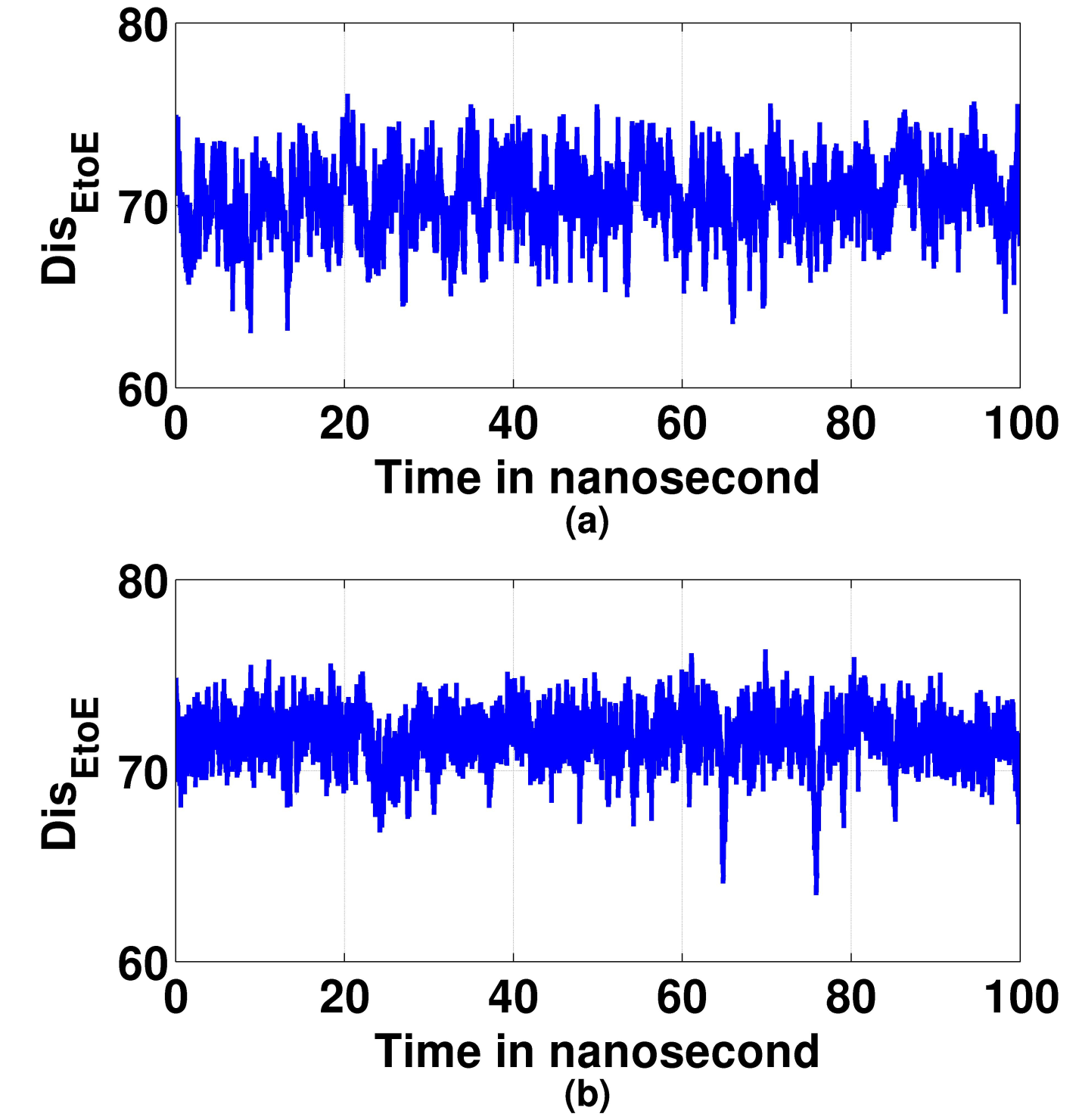** |
| --- |

**Fig B: Comparison of end to end distance (Dis_EtoE_ in Å) of DNA using ff99bsc0 (shown in (a)) and ff99parmbsc1 (shown in (b)) forcefields for DNA plus water system.** Dis_EtoE_ is distance between center of mass of 1^st^ base pair to center of mass of last base pair of DNA.

| **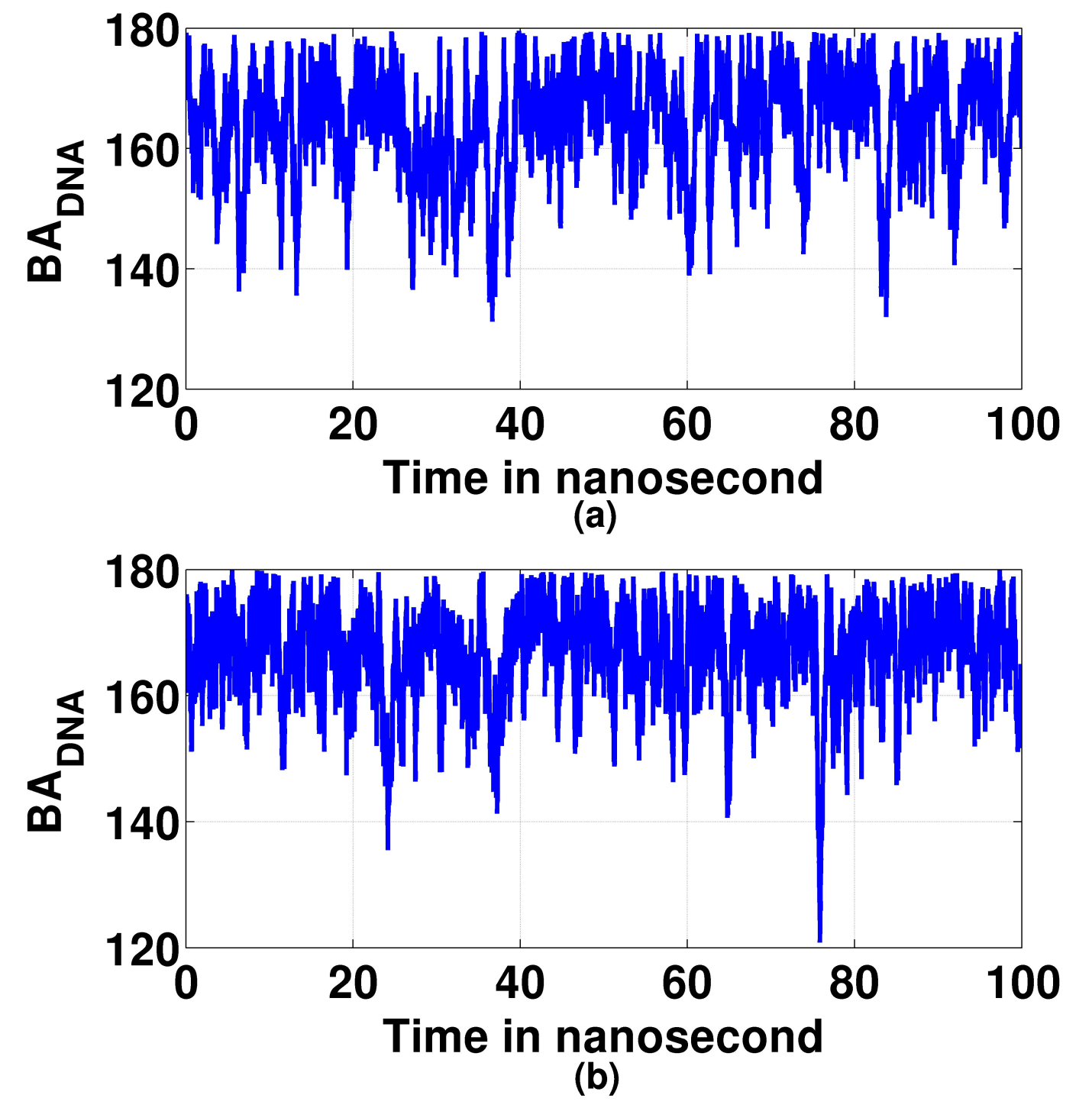** |
| --- |

**Fig C: Comparison of bending angle of DNA (BA_DNA_ in degree) using ff99bsc0 (shown in (a)) and ff99parmbsc1 (shown in (b)) forcefields for DNA plus water system.** BA_DNA_ is absolute angle between center of mass of 1^st^ base pair (1 and 46), center of mass of middle base pair (12 and 35) and center of mass of last base pair (23 and 24) of DNA.

**
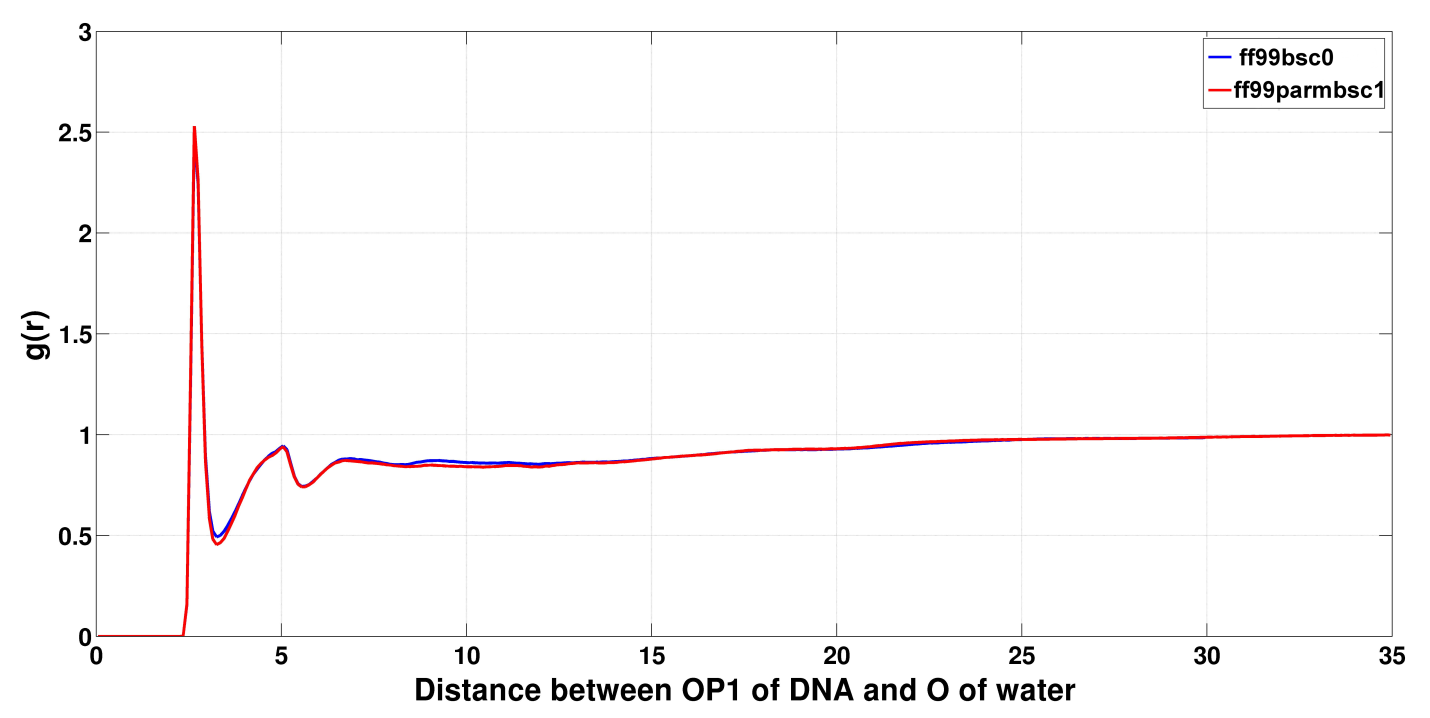
**

**Fig D: Comparison of radial distribution function of oxygen of water around OP1 atom of DNA backbone using ff99bsc0 and ff99parmbsc1 forcefields for DNA plus water system.**

**
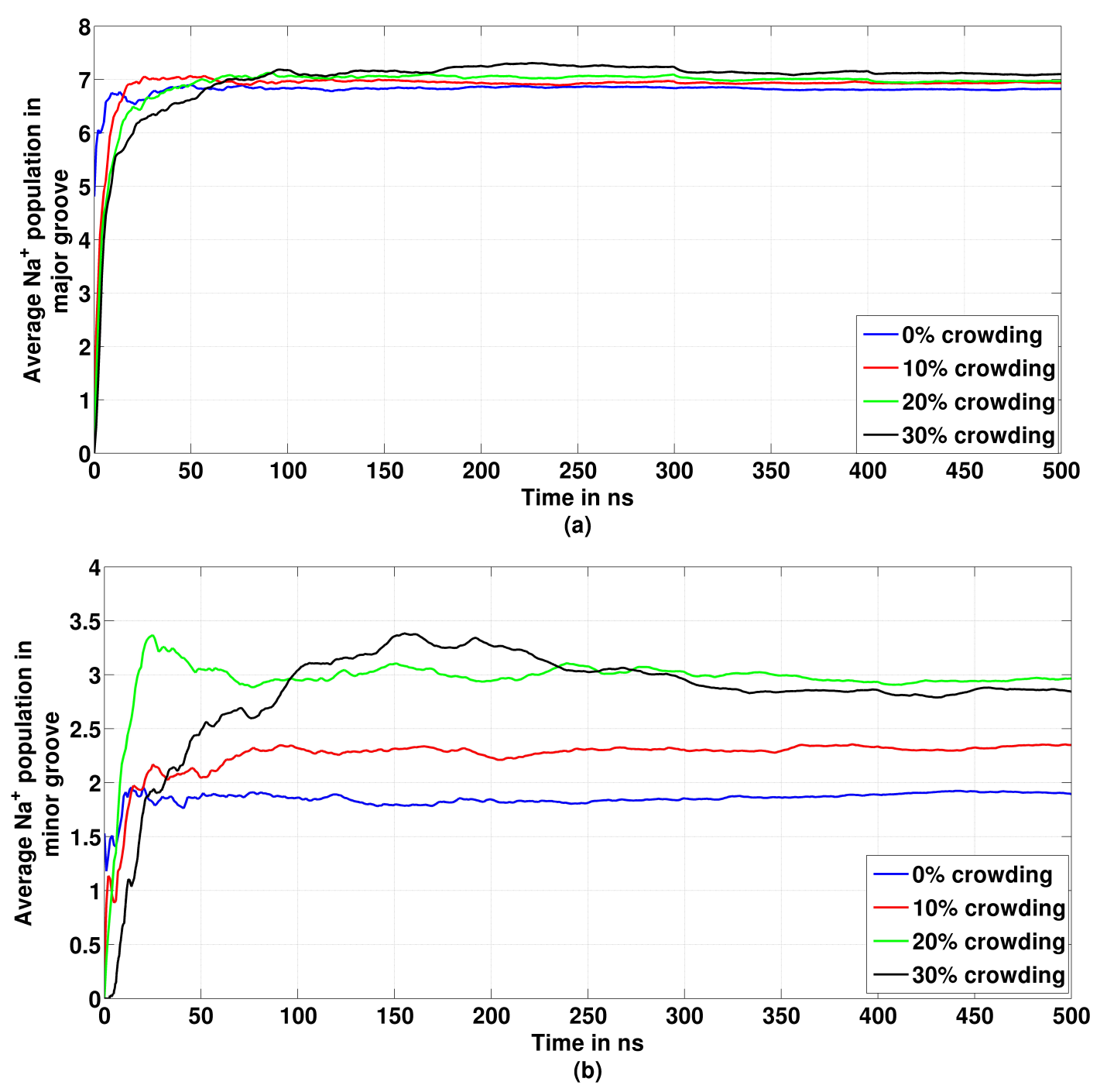
**

**Fig E: Time-averaged population of Na^+^ around DNA at different crowding concentrations in (a) major groove and (b) minor groove of DNA.**(R = 10.25 Å, A was taken as 33 to 147 degrees for minor groove and rest for major groove and the value of D was taken from 2 to 22).

**2: GRID Description:**

To calculate­ the density (in 3D) of water and EG around the DNA we have used grid command of cpptraj [2]. For this calculation, a 3D region R is defined around the desired section of DNA as N_x_* Δx* N_y_*Δy*N_z_ *Δz Å^3^. Where N_x_, N_y_ and N_z_ represents the number of bins or voxels along X, Y and Z-axis. The bin spacing = Δx = Δy = Δz = 0.5 Å was used for the calculation. The 3D-density of a molecule, M, at grid bin k can be written as:

$$g\left( k \right)= \frac{n_{k}}{N_{f}V_{k}\rho^{o}}$$

Where, $n_{k}$ is the total number of M at bin *k*, $N_{f}$ is the total number of frames for analysis, *V_k_* represents the volume of bin k and $\rho^{o}$ is the bulk density of molecule M.

The convergence of water can be seen in Figures F-I for 0%, 10%, 20% and 30% crowding concentrations respectively. The convergence of EG can be seen in Figures J-L for 10%, 20% and 30% crowding concentrations respectively. The local number density of water (Figure F-I) and EG (Figures J-L) molecules are shown for two regions of DNA (both the regions were selected on the basis of minimum fluctuation as it can be seen in RMSF plot). As it can be seen from Figures F-I there is slight or no change in isosurface density values at 100ns, 200ns and 300ns for both the regions of DNA at all the crowding concentrations. This ensures the convergence of water around both the regions of DNA. The EG molecules also show convergence around both the regions of DNA in between 200 to 300 ns as there is slight or no change of EG isosurface densities around these regions.


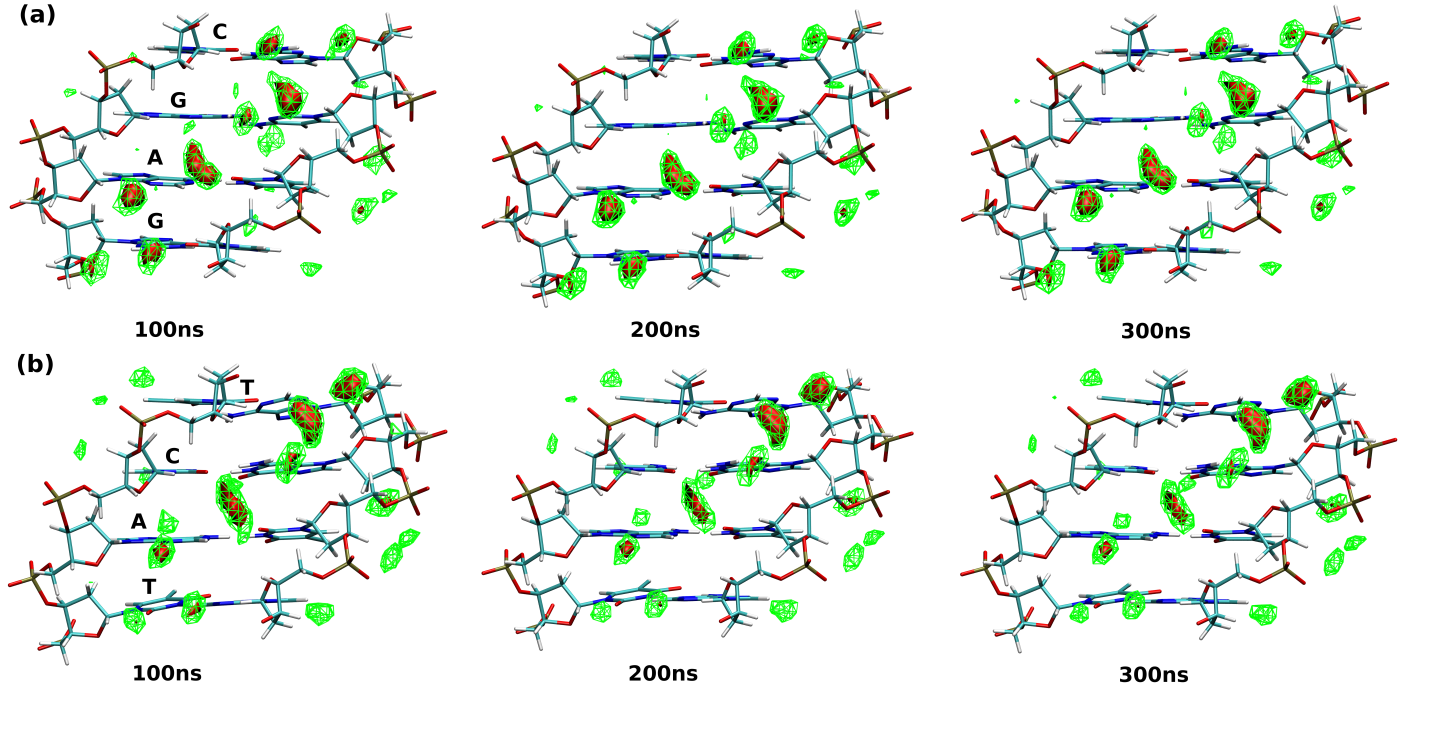


**Fig F: Convergence of water around local regions of DNA at 0% crowding concentration.** Panel (a) and (b) show the convergence of water around GAGC and TACT, respectively. Two isosurface density values are shown, 7 (wireframe green) and 9 (solid red).

| **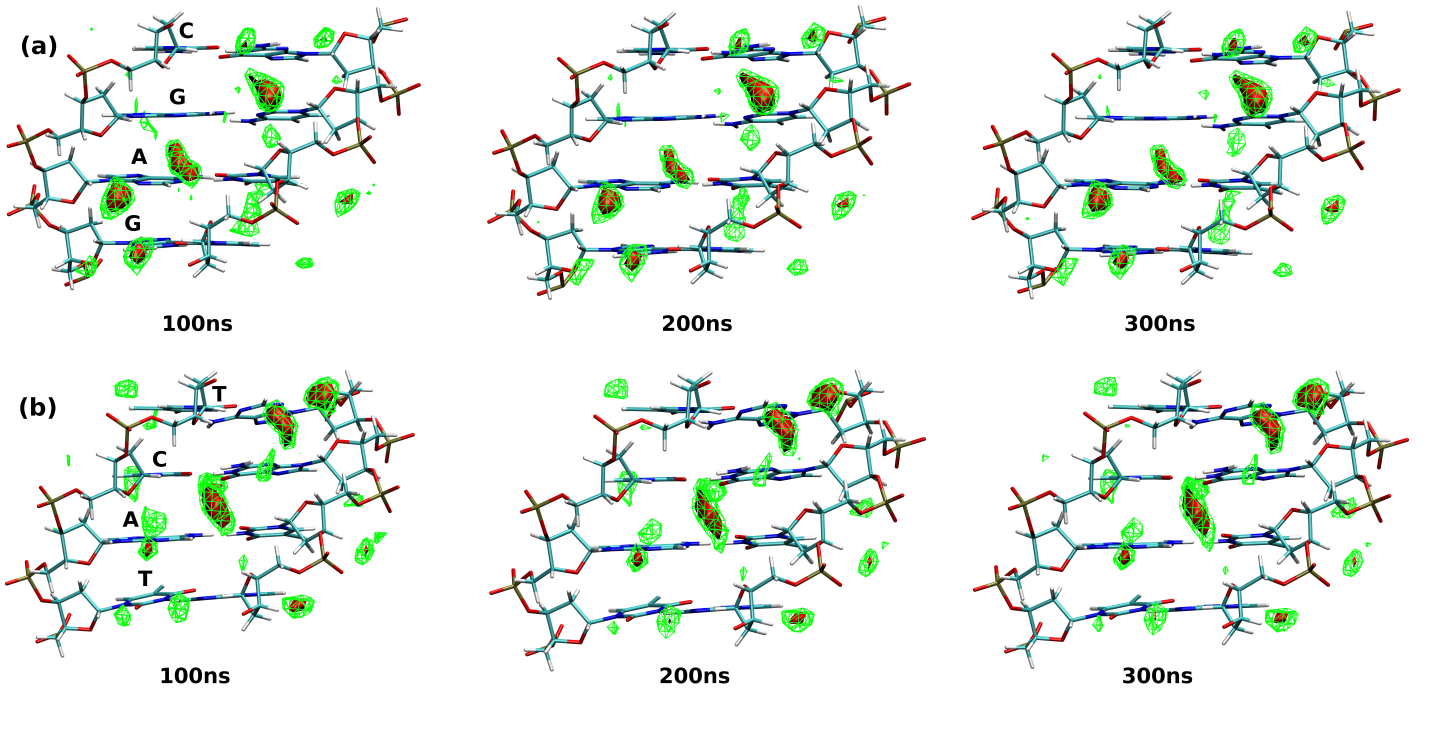** |
| --- |

**Fig G: Convergence of water around local regions of DNA at 10% crowding concentration.** Panel (a) and (b) show the convergence of water around GAGC and TACT, respectively. Two isosurface density values are shown, 7 (wireframe green) and 9 (solid red).

| **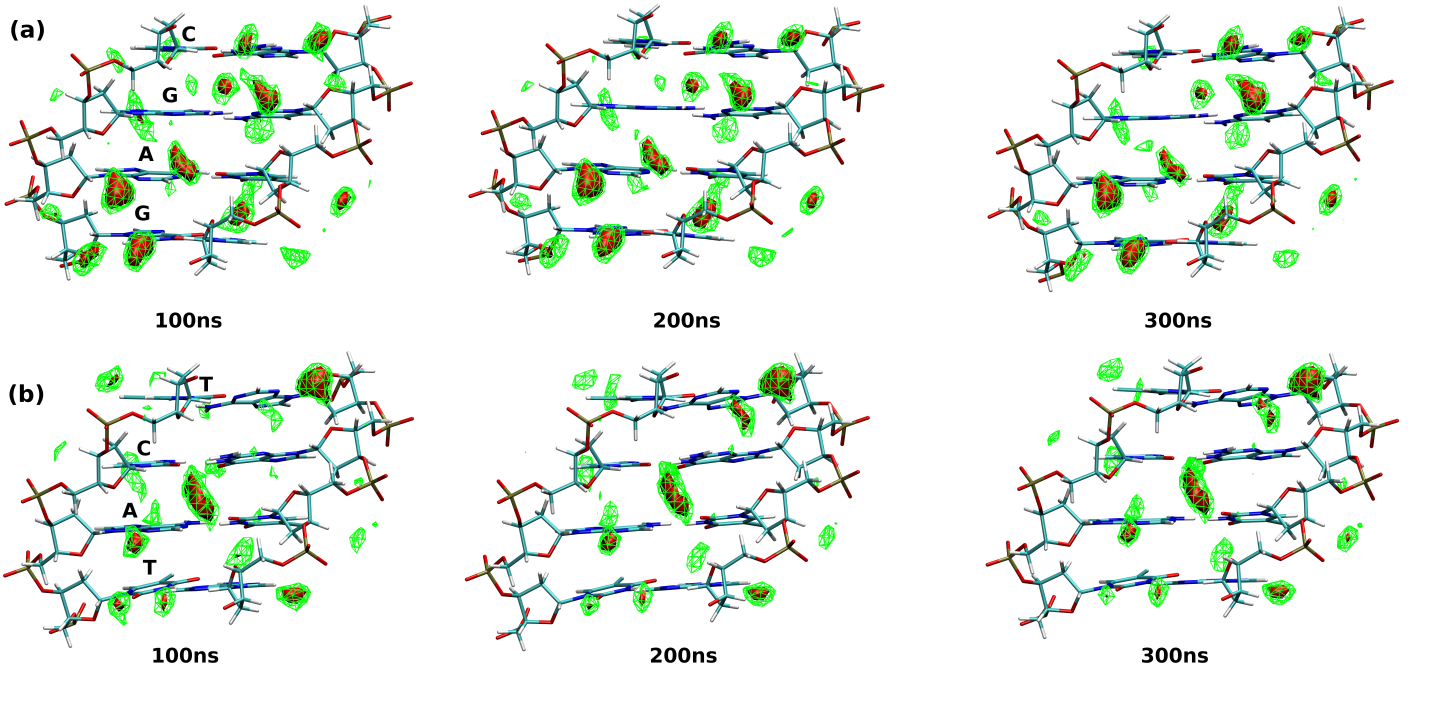** |
| --- |

**Fig H: Convergence of water around local regions of DNA at 20% crowding concentration.** Panel (a) and (b) show the convergence of water around GAGC and TACT, respectively. Two isosurface density values are shown, 7 (wireframe green) and 9 (solid red).

| 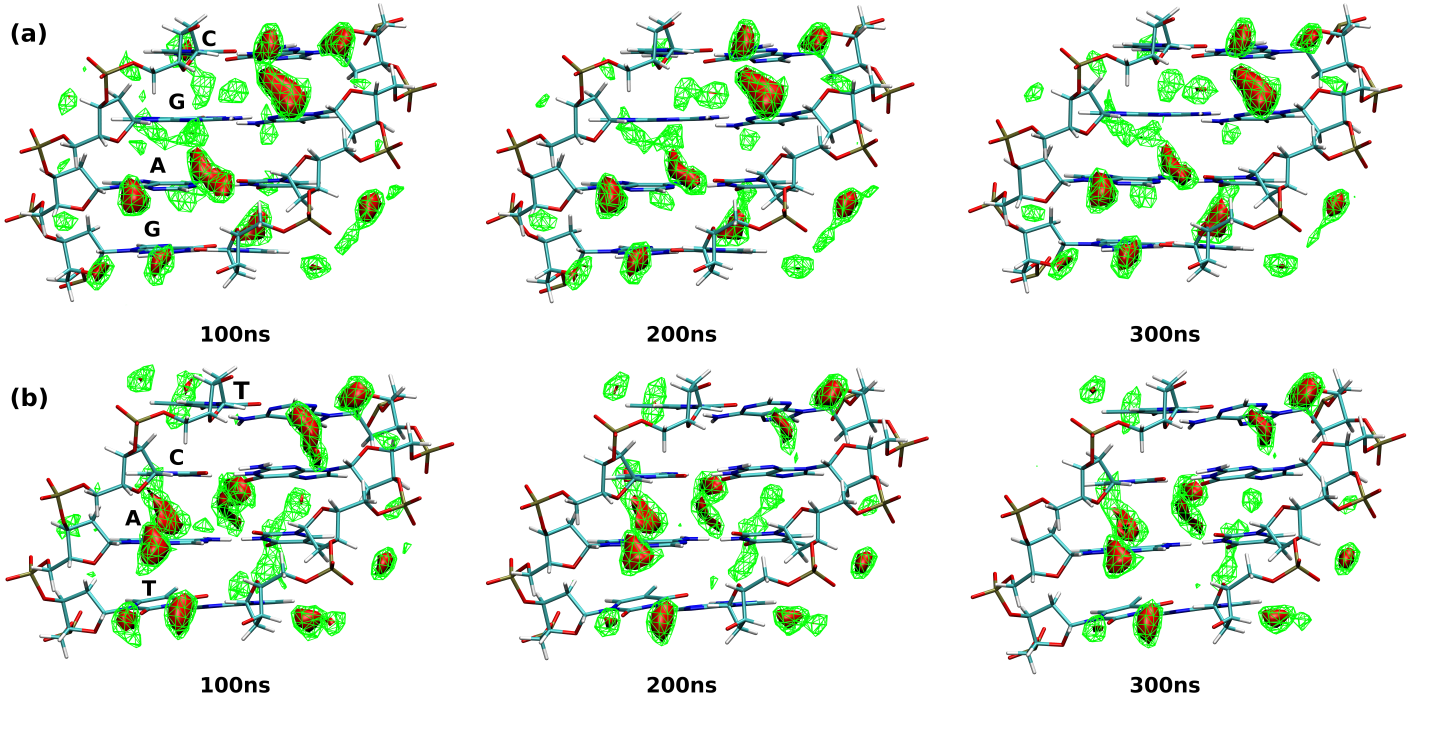 |
| --- |

**Fig I: Convergence of water around local regions of DNA at 30% crowding concentration.** Panel (a) and (b) show the convergence of water around GAGC and TACT, respectively. Two isosurface density values are shown, 7 (wireframe green) and 9 (solid red).

| 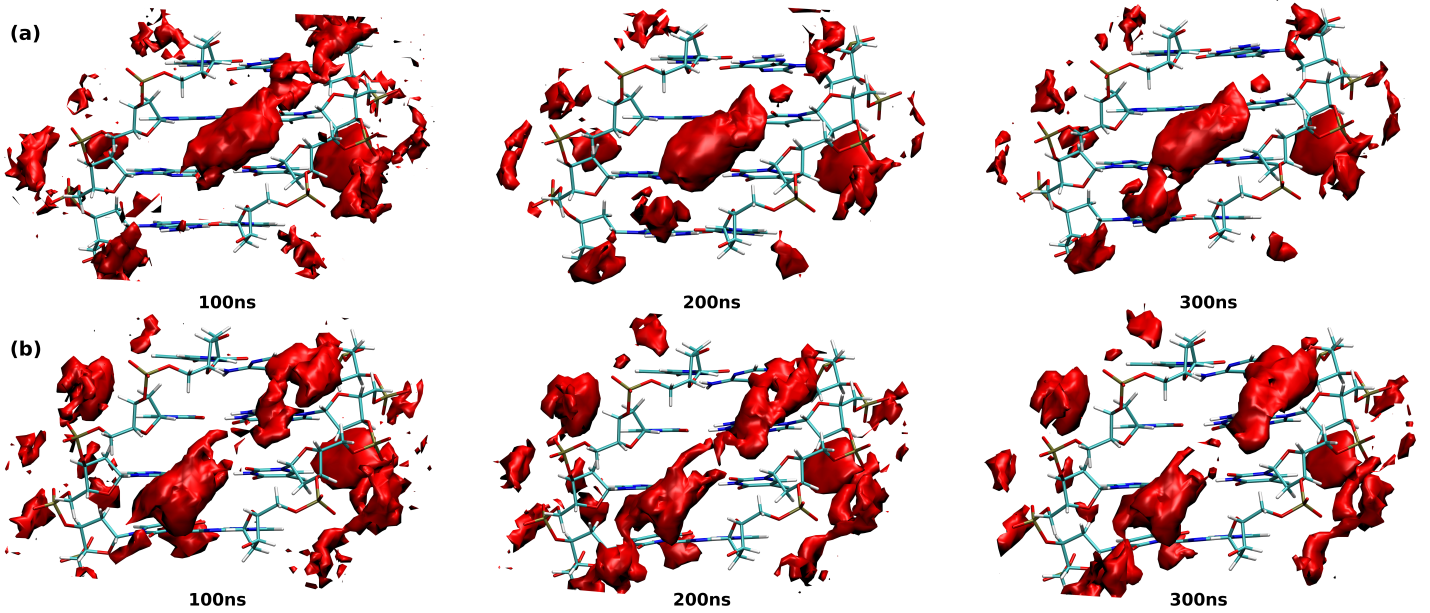 |
| --- |

**Fig J: Convergence of EG around local regions of DNA at 10% crowding concentration.** Panel (a) and (b) show the convergence of water around GAGC and TACT, respectively. Isosurface densities of 27 or more are shown.

| **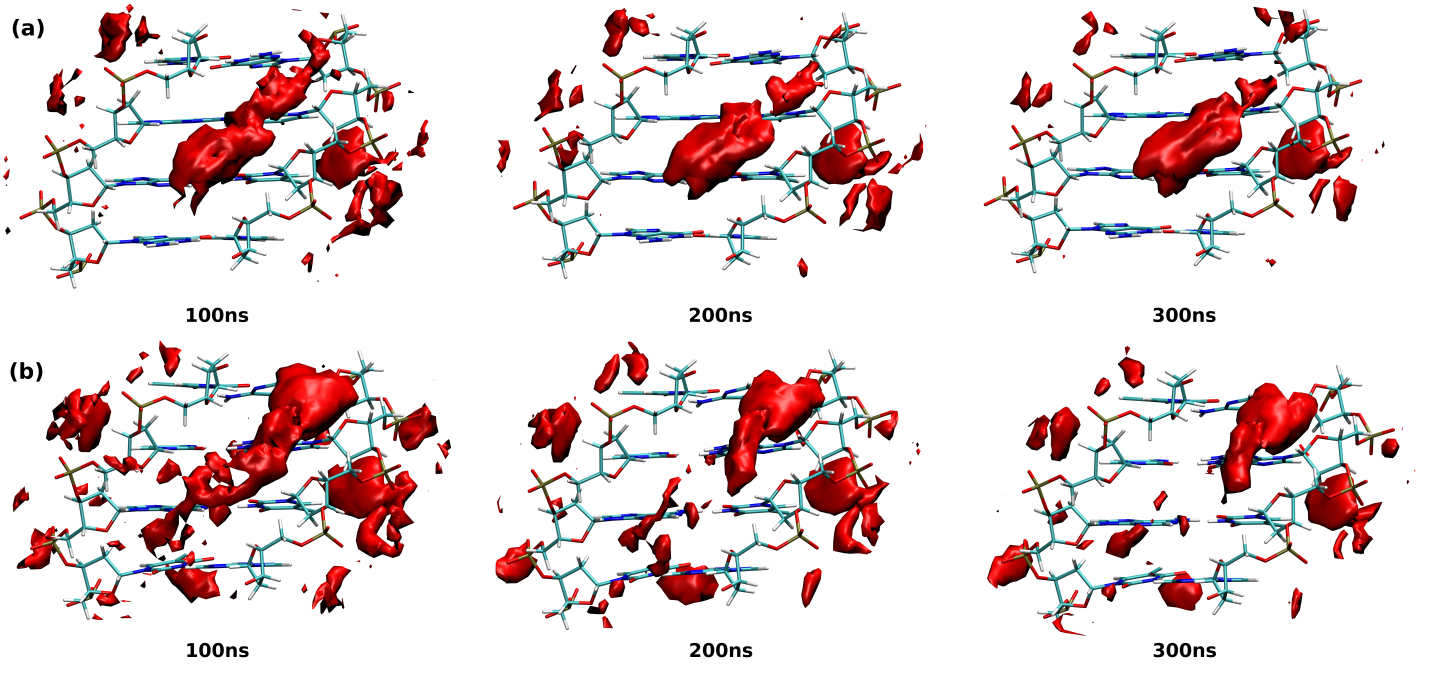** |
| --- |

**Fig K: Convergence of EG around local regions of DNA at 20% crowding concentration.** Panel (a) and (b) show the convergence of water around GAGC and TACT, respectively. Isosurface densities of 25 or more are shown.

| 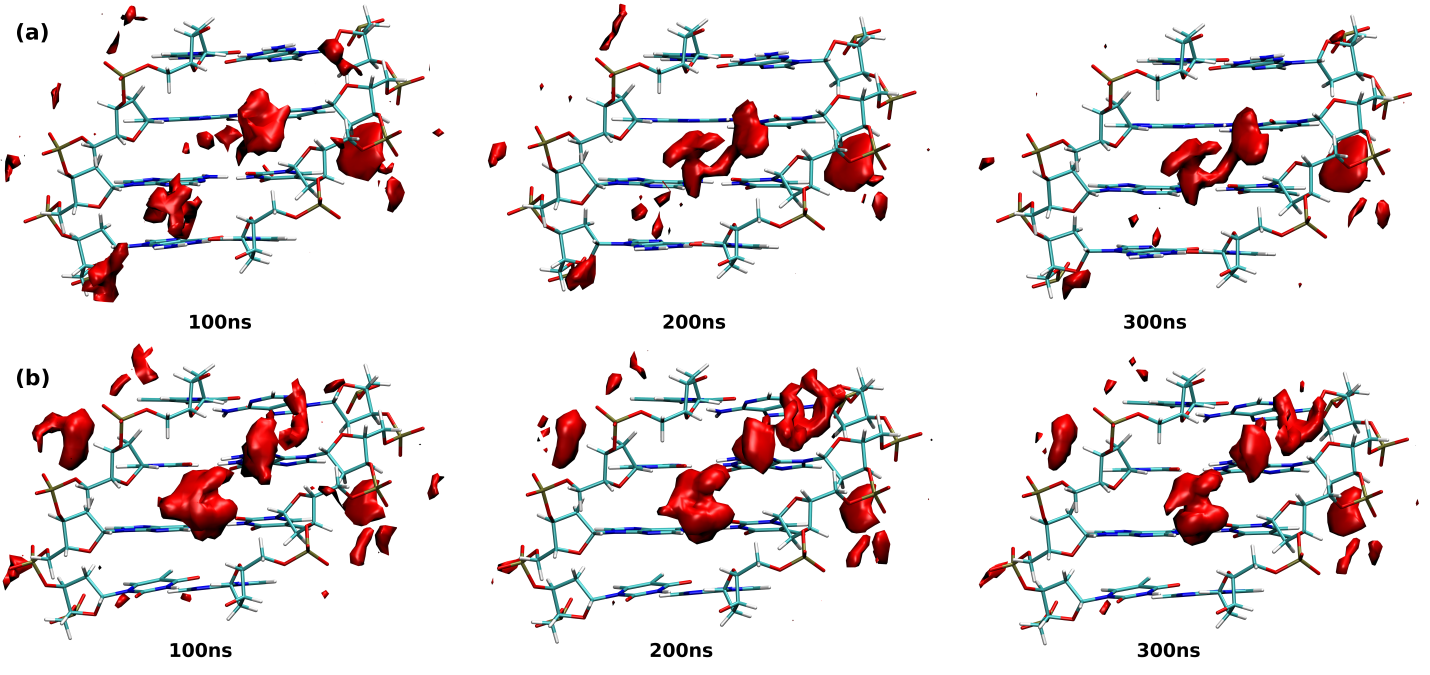 |
| --- |

**Fig L: Convergence of EG around local regions of DNA at 30% crowding concentration.** Panel (a) and (b) show the convergence of water around GAGC and TACT, respectively. Isosurface densities of 25 or more are shown.

**
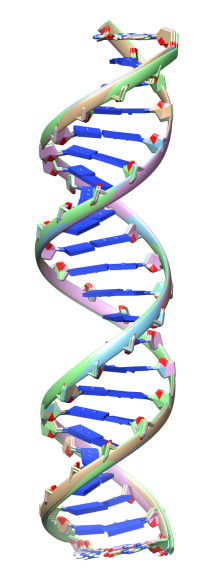
**

**Fig M: Average structure overlap of DNA at 0% (tan), 10% (blue), 20% (pink) and 30% (green) crowding concentrations, respectively.**

| **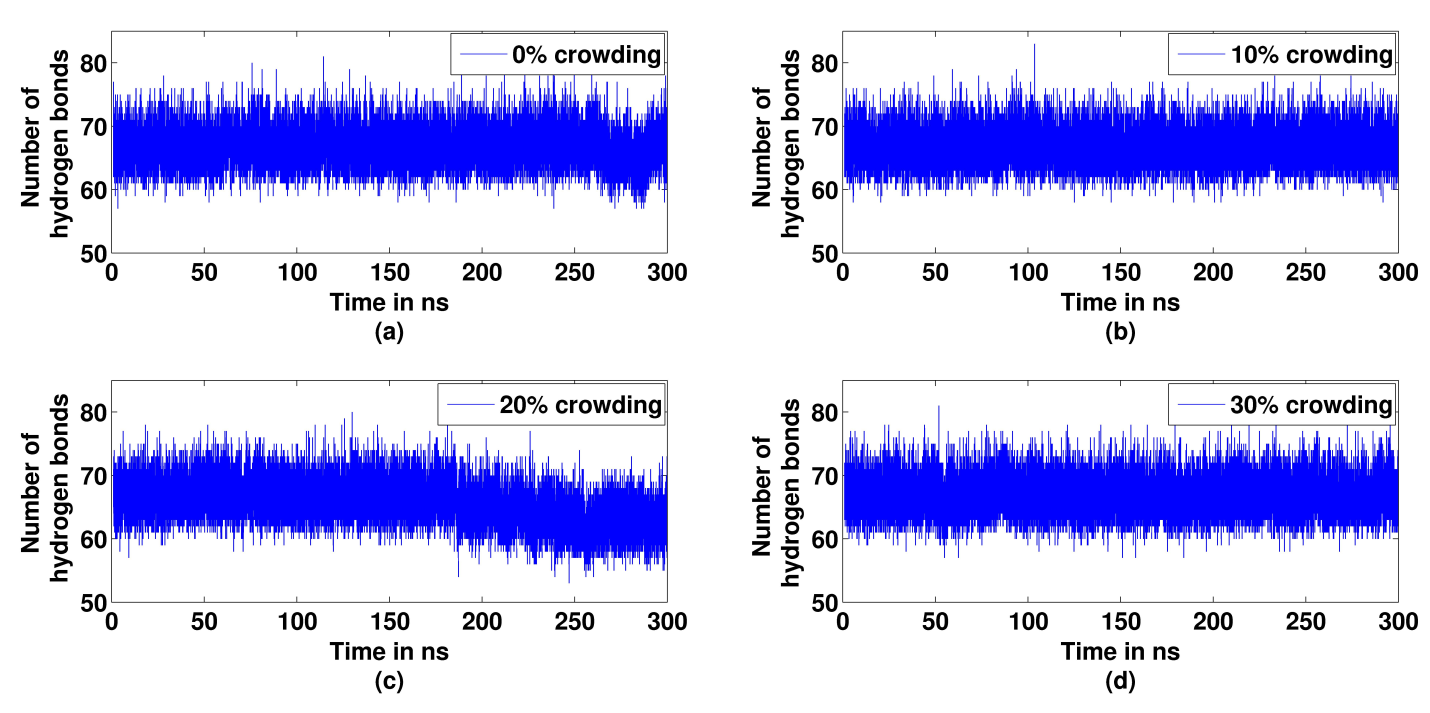** |
| --- |

**Fig N: Number of intramolecular hydrogen bonds of DNA at (a) 0%, (b) 10%, (c) 20% and, (d) 30% crowding concentrations, respectively.**

| **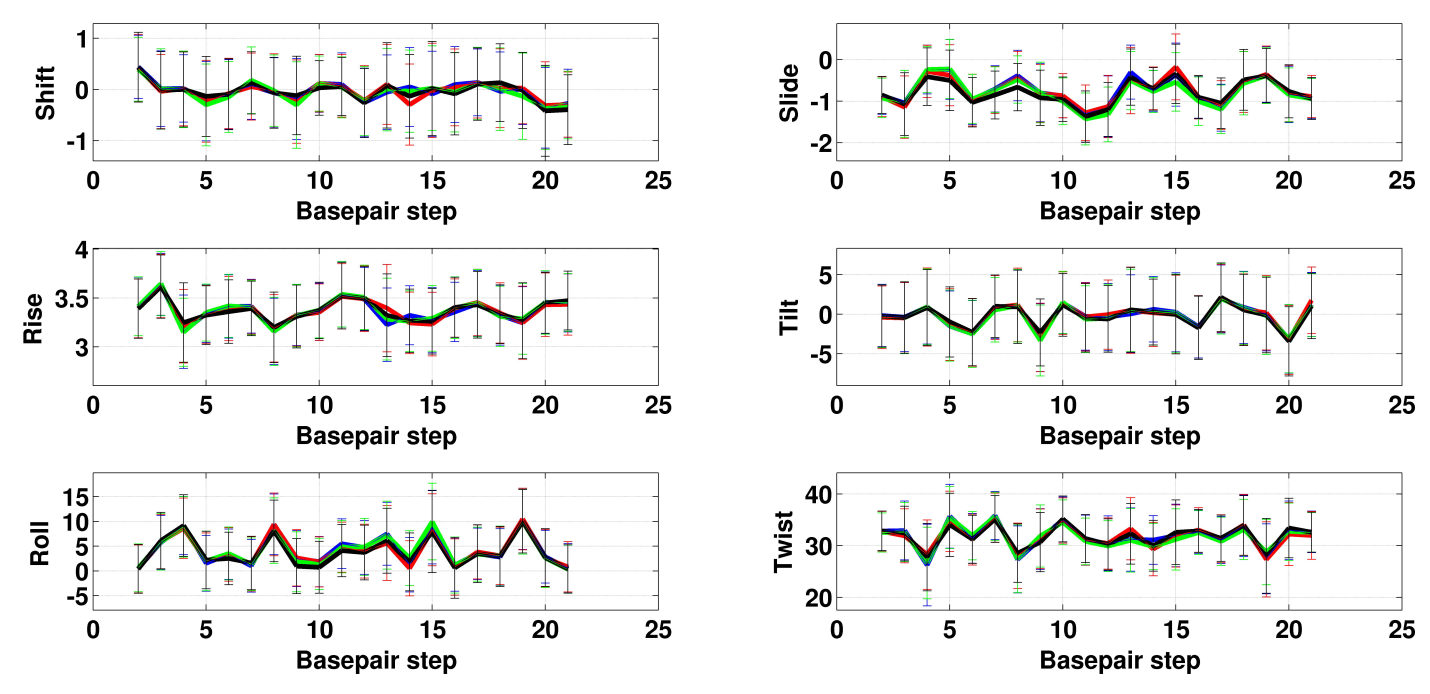** |
| --- |

**Fig O: Base-pair step parameters of DNA. Blue, red, green and black colors represent parameter values at 0%, 10%, 20% and 30% crowding environment.**

| **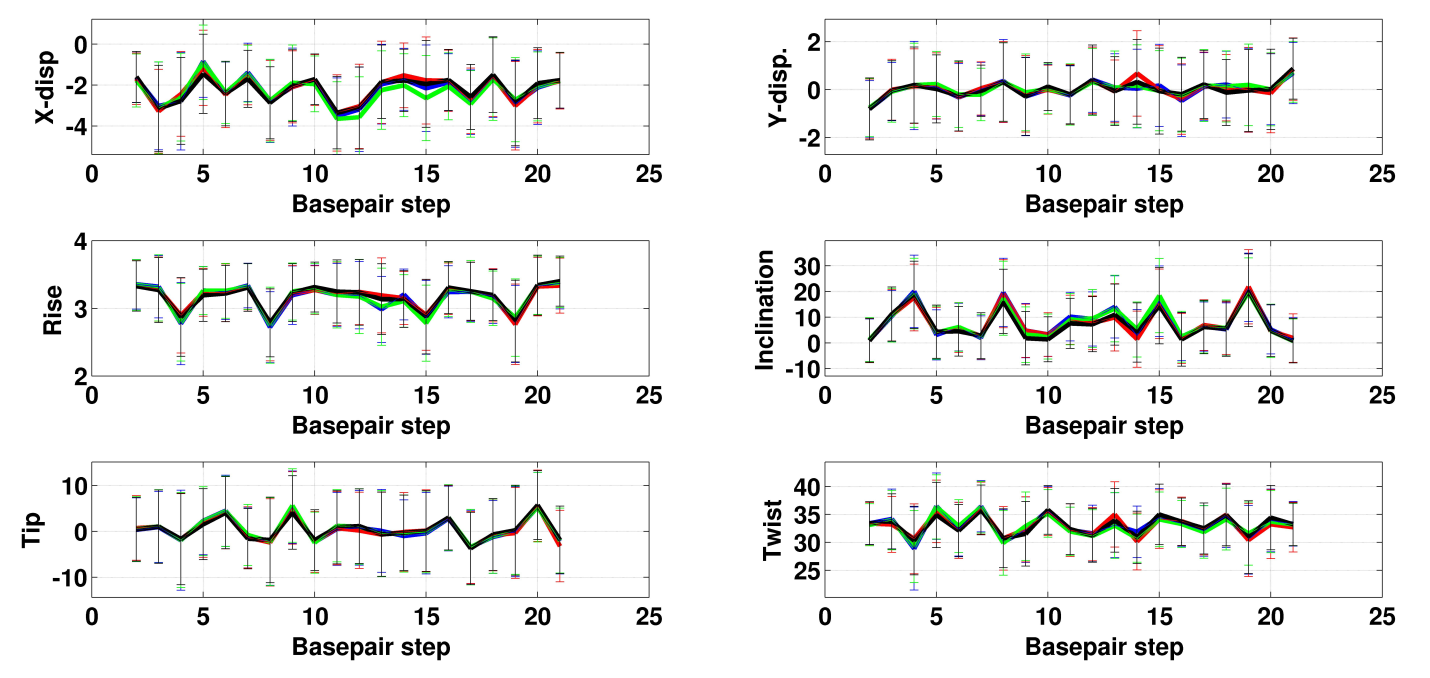** |
| --- |

**Fig P: Base-pair helix parameters of DNA. Blue, red, green and black colors represent parameter values at 0%, 10%, 20% and 30% crowding environment.**

| **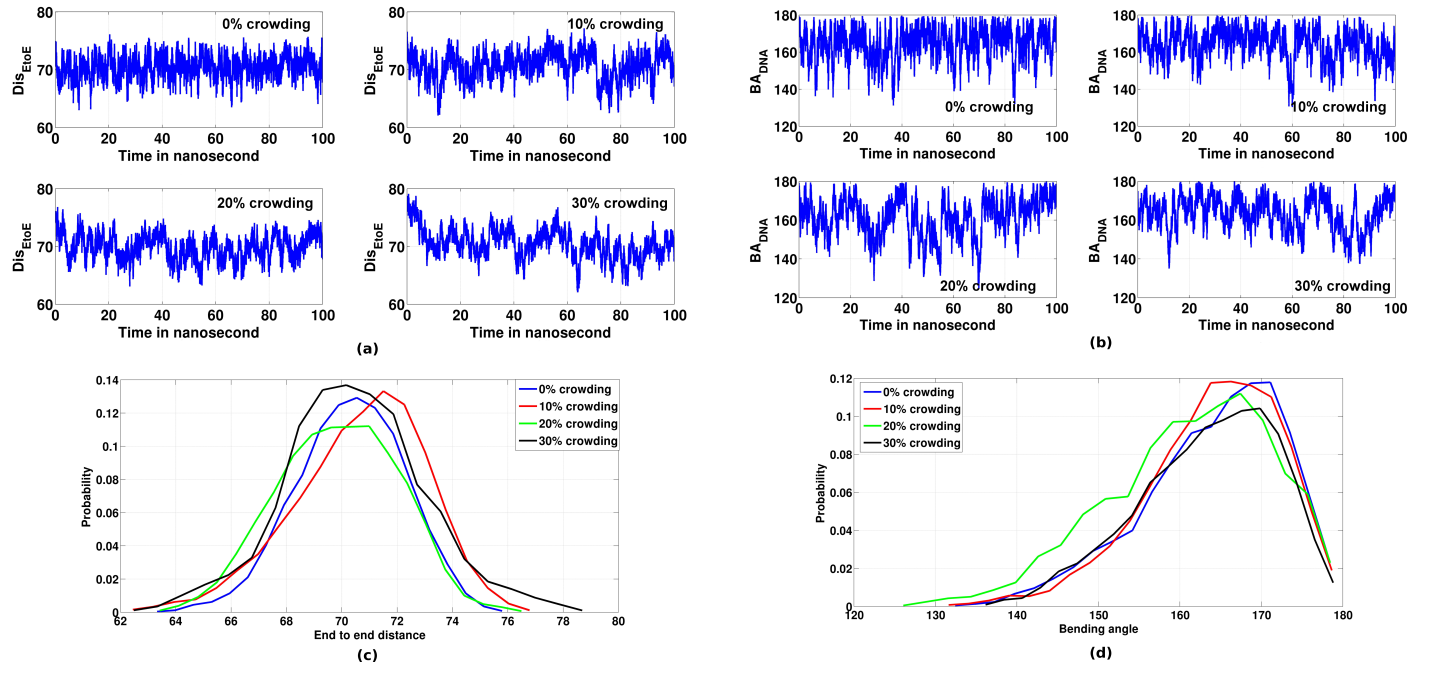** |
| --- |

**Fig Q: DNA structural fluctuation calculated in terms of (a) DNA end to end distance in** Å, **(b) DNA bending angle in degree, (c) probability density function (PDF) of DNA end to end distance and (d) PDF of DNA bending angle.**

**3: PCA similarity Index:**

To understand the effect of crowding on the dynamics of DNA, we have performed principal component analysis and calculated two similarity indices, γ and ζ (described in the article by Perez *et al.,*[3]) between the first ten eigenvectors for each pair of crowding systems. In the calculation of first similarity index γ, it is implicitly assumed that all the ten eigenvectors contribute equally to the dynamical behavior of the DNA. The absolute similarity index, γ, for two DNA trajectories A and B can be calculated using the following formula:


$$\gamma_{AB}= \frac{1}{n}\sum_{j=1}^{n} \sum_{i=1}^{n} \left( \nu_{i}^{A}\nu_{j}^{B} \right)^{2}$$

where, n is the minimum number of eigenvectors needed to define the set of important eigenvectors, $\left\{ \mu_{i} \right\}^{A}$ and $\left\{ \mu_{i} \right\}^{B}$corresponding to two trajectories A and B respectively. These $\left\{ \mu_{i} \right\}^{A}$ and $\left\{ \mu_{i} \right\}^{B}$capture the essential dynamics of DNA in the two trajectories. For our system, we have taken n = 10, which essentially capture 80% to 90% of the DNA dynamics [3]. Further, $\nu_{i}^{X}$ represents ith-unitary eigenvector of trajectory X (*i.e.* $\nu_{i}^{X}={\mu_{i}^{X}}/\left| \mu_{i}^{X} \right|$). The value of $\gamma$ varies between 0 (completely different movements) and 1 (identical movements).

The calculation of second similarity index $\zeta$ does not involve the assumption of equal contribution of eigenvectors to the variance of DNA trajectory. It considers the relative importance of different eigenvectors towards the dynamics of a DNA trajectory by including the corresponding eigenvalues. $\zeta$ can be calculated for two DNA trajectories using following formula:

$$\zeta_{AB}= \frac{2\sum_{i=1}^{z} \sum_{j=1}^{z} \left\{ \left( \nu_{i}^{A}\nu_{j}^{B} \right)\frac{exp\left[ -\frac{\left( \Delta x \right)^{2}}{\lambda_{i}^{A}}-\frac{\left( \Delta x \right)^{2}}{\lambda_{j}^{B}} \right]}{\sum_{i=1}^{z} exp\left[ -\frac{\left( \Delta x \right)^{2}}{\lambda_{i}^{A}} \right]\sum_{j=1}^{z} exp\left[ -\frac{\left( \Delta x \right)^{2}}{\lambda_{j}^{B}} \right]} \right\}^{2}}{\sum_{i=1}^{z} \left\{ \frac{exp\left[ -2\frac{\left( \Delta x \right)^{2}}{\lambda_{i}^{A}} \right]}{\left( \sum_{i=1}^{z} exp\left[ \frac{\left( \Delta x \right)^{2}}{\lambda_{i}^{A}} \right] \right)^{2}} \right\}^{2}+ \sum_{j=1}^{z} \left\{ \frac{exp\left[ -2\frac{\left( \Delta x \right)^{2}}{\lambda_{j}^{B}} \right]}{\left( \sum_{j=1}^{z} exp\left[ \frac{\left( \Delta x \right)^{2}}{\lambda_{j}^{B}} \right] \right)^{2}} \right\}^{2}}$$

Where, $\lambda_{i}$is the eigenvalue (in Å^2^) corresponding to the eigenvector$\mu_{i}$, $\nu_{i}$ represents the unitary vector of $\mu_{i}$ and *Δx* is the common displacement along different eigenvectors. Higher values of *Δx* imply that only first few modes will be important in comparison while smaller values of *Δx* imply that higher modes also play role in comparison. In this calculation we have took *Δx* = 0.1Å.

| **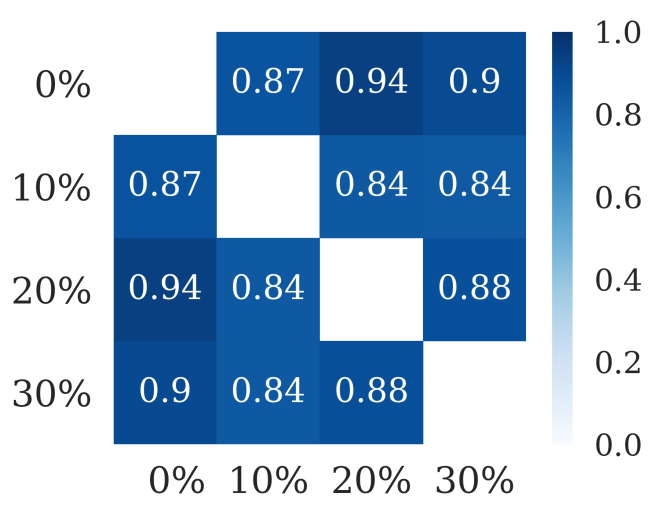** |
| --- |

**Fig R: PCA similarity index for first 10 major modes of motions of DNA at different crowding concentrations.** The upper and lower triangular matrices represent the similarity indices$\gamma$and$\zeta$, respectively.

| \| 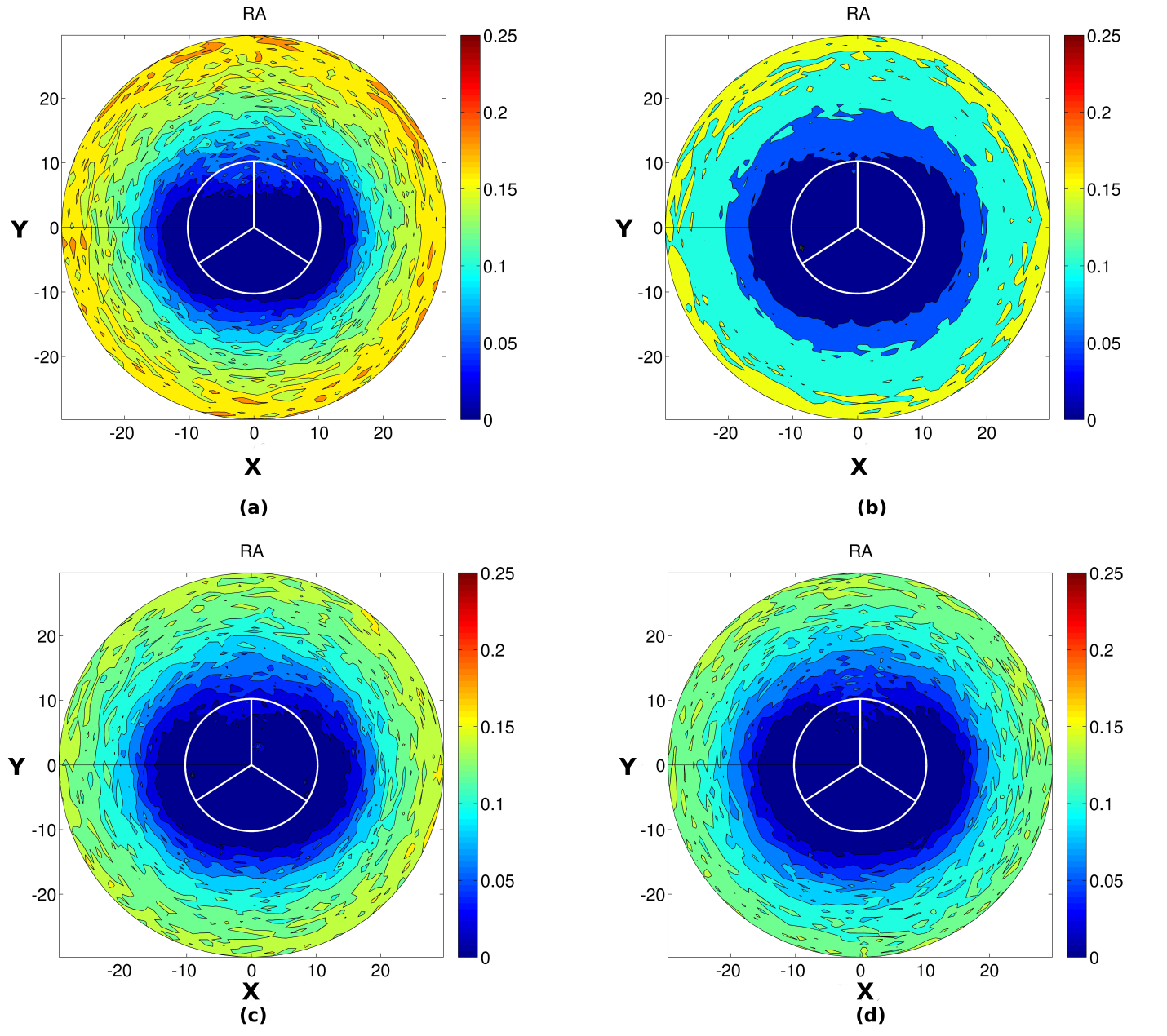 \| \| --- \| |
| --- | --- |

**Fig S: Average Cl^-^ distribution calculated using CHC after transforming the R and A to cartesian coordinates (both X and Y are in Å)(a) 0% crowding (b) 10% crowding (c) 20% crowding (d) 30% crowding.** The color scale blue to red represents increasing molarities. The upper semicircle represents the major groove and lower represents the minor groove of DNA. The vertical radial vector indicates the center of the major groove.

| 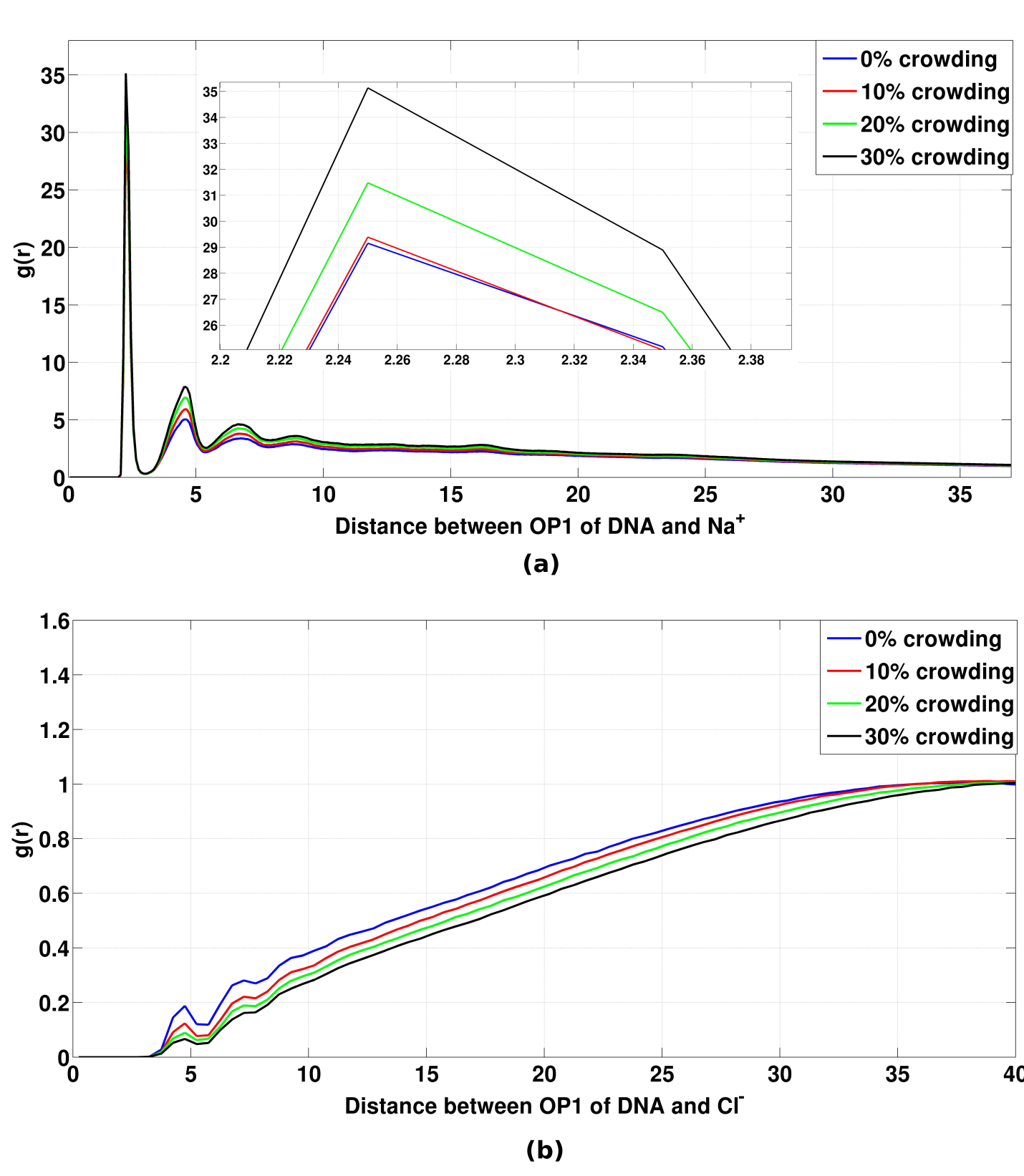 |
| --- |

|  |
| --- |

**Fig T: Pair correlation functions (g(r)) of ions around DNA at different crowding concentration.** (a) g(r) of Na^+^ ion around OP1 atom of DNA. The x-axis represents the distance (in Å) between Na^+^ and OP1, and Y-axis represents g(r). (b) g(r) of Cl^-^ ion around OP1 atom of DNA. X-axis represents the distance between Cl^-^ and OP1 of DNA and Y-axis represents g(r).

|  |
| --- |

| 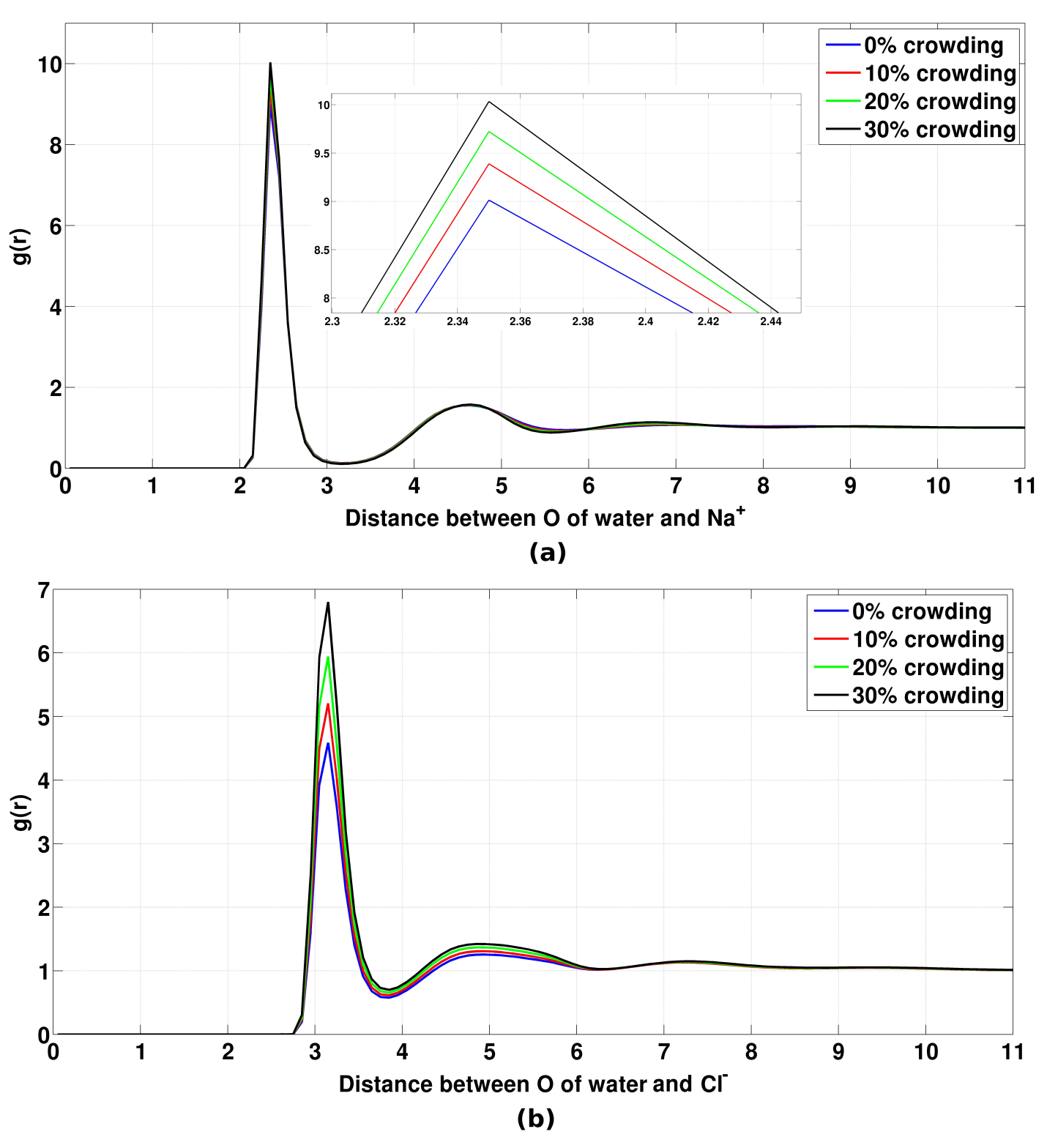 |
| --- |

**Fig U: Pair correlation functions (g(r)) of ions around water.** (a) g(r) of Na^+^ ion around oxygen of water. The x-axis represents the distance (in Å) between Na^+^ and oxygen of water and Y-axis represents g(r). (b) g(r) of Cl^-^ ion around oxygen of water. The x-axis represents the distance between Cl^-^ and oxygen of water and Y-axis represents g(r).

| 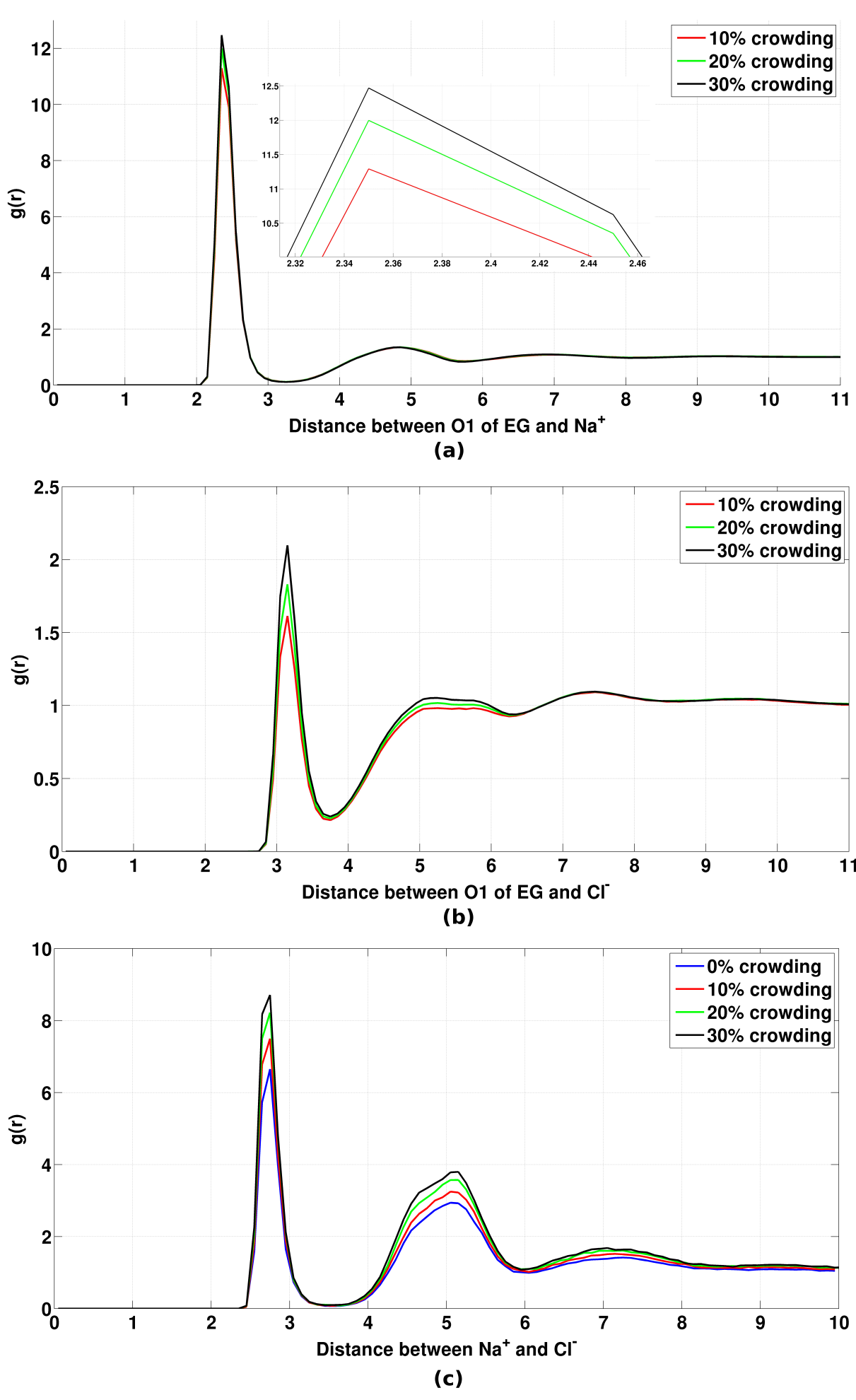 |
| --- |

**Fig V: Pair correlation function of (a) Na^+^ around oxygen of EG, (b) Cl^-^ around oxygen of EG, and (c) Cl^-^ ion around Na^+^ at different crowding concentrations.**

| **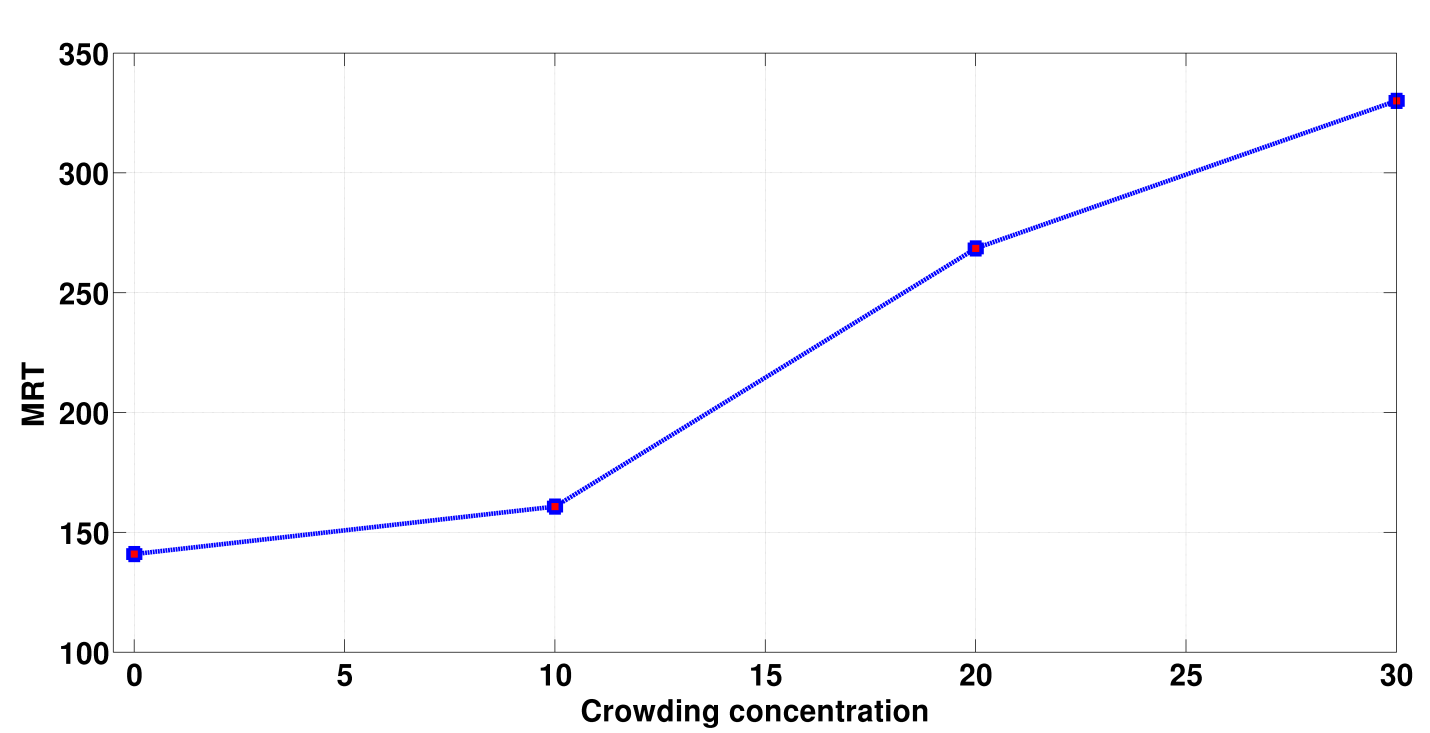** |
| --- |

**Fig W: Mean Residence Time (in picosecond) of water molecules in the first hydration shell of DNA at different crowding concentrations.**

| 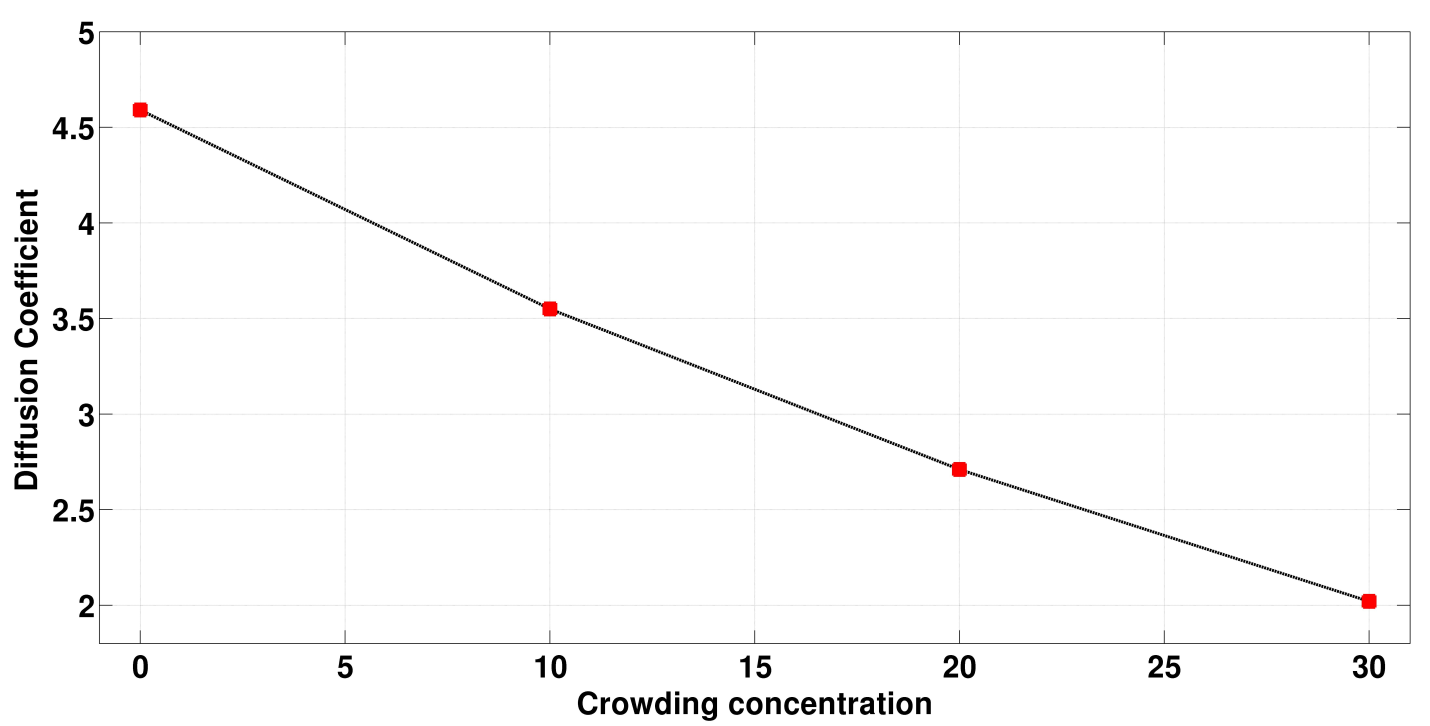 |
| --- |

**Fig X: Diffusion coefficient (**× **10^-5^ cm^2^/s) of water at 0%, 10%, 20% and 30% crowding concentrations.**

| **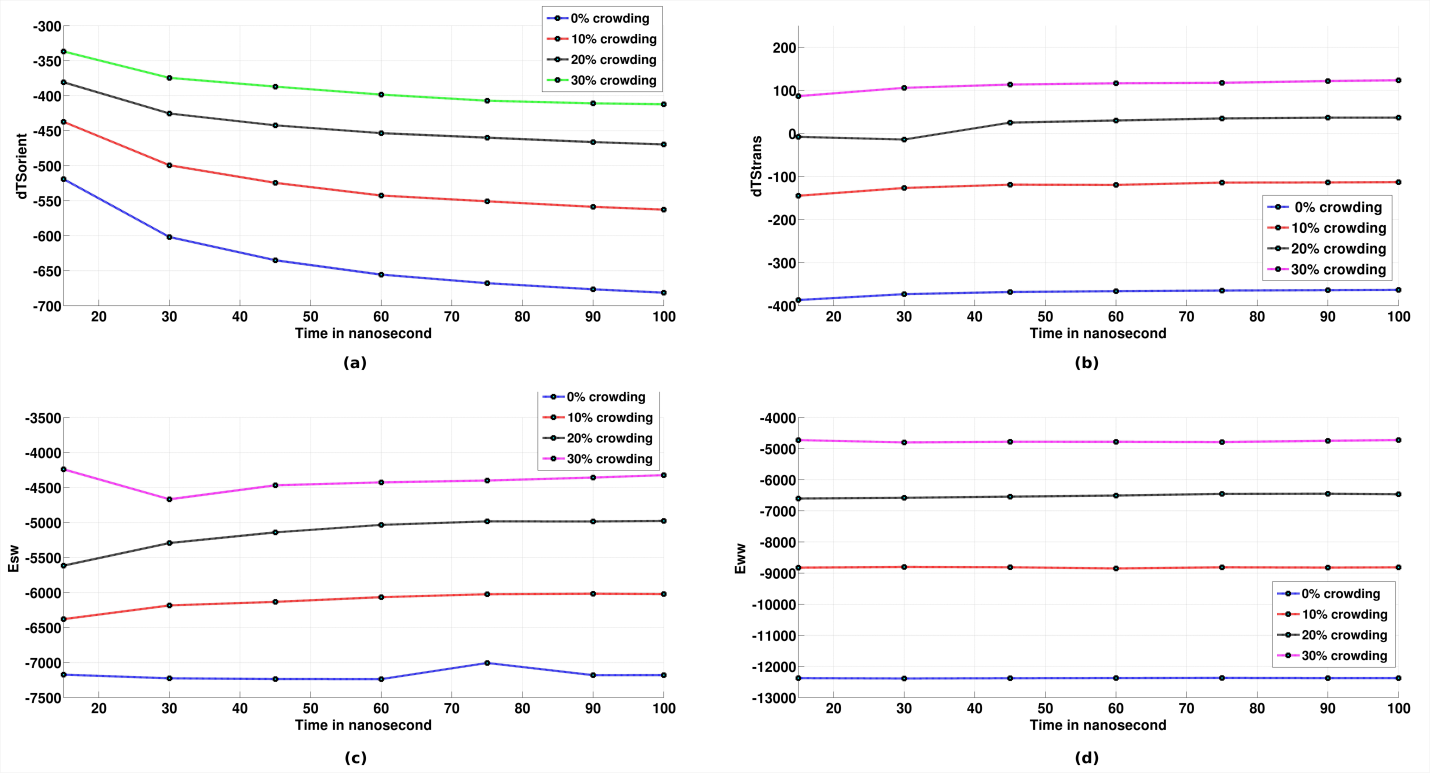** |
| --- |

**Fig Y: Convergence of (a) orientational entropy, (b) translational entropy, (c) DNA-water interaction potential, (d) water-water interaction potential in kcal/mol at different crowding concentrations.**

**References**

1. Ivani I, Dans PD, Noy A, Pérez A, Faustino I, Hospital A, et al. Parmbsc1: a refined force field for DNA simulations. Nat Methods. Nature Publishing Group; 2016;13: 55–58. doi:10.1038/nmeth.3658

2. Roe DR, Cheatham TE. PTRAJ and CPPTRAJ: Software for Processing and Analysis of Molecular Dynamics Trajectory Data. J Chem Theory Comput. 2013;9: 3084–3095. doi:10.1021/ct400341p

3. Pérez A, Blas JR, Rueda M, López-Bes JM, de la Cruz X, Orozco M. Exploring the Essential Dynamics of B-DNA. J Chem Theory Comput. 2005;1: 790–800. doi:10.1021/ct050051s
